# Rac1 GTPase Regulates the βTrCP-Mediated Proteolysis of YAP Independently of the LATS1/2 Kinases

**DOI:** 10.3390/cancers16213605

**Published:** 2024-10-25

**Authors:** Chitra Palanivel, Tabbatha N. Somers, Bailey M. Gabler, Yuanhong Chen, Yongji Zeng, Jesse L. Cox, Parthasarathy Seshacharyulu, Jixin Dong, Ying Yan, Surinder K. Batra, Michel M. Ouellette

**Affiliations:** 1 Department Internal Medicine, University of Nebraska Medical Center, Omaha, NE 68198, USAtsomers@unmc.edu (T.N.S.); bmwobig15@gmail.com (B.M.G.); 2 Department of Radiation Oncology, University of Nebraska Medical Center, Omaha, NE 68198, USA; yyan@unmc.edu; 3 Eppley Institute for Research in Cancer, University of Nebraska Medical Center, Omaha, NE 68198, USA; cheny@unmc.edu (Y.C.); yongji.zeng@bcm.edu (Y.Z.); dongj@unmc.edu (J.D.); 4 Department of Pathology, Microbiology and Immunology, University of Nebraska Medical Center, Omaha, NE 68198, USA; jcox@unmc.edu; 5 Department of Biochemistry and Molecular Biology, University of Nebraska Medical Center, Omaha, NE 68198, USA; p.seshacharyulu@unmc.edu (P.S.); sbatra@unmc.edu (S.K.B.)

**Keywords:** YAP, Rac1, pancreatic cancer, ubiquitin ligases, βTrCP, Ras, Hippo, GTPase

## Abstract

**Background:** Oncogenic mutations in the *KRAS* gene are detected in >90% of pancreatic cancers (PC). In genetically engineered mouse models of PC, oncogenic *KRAS* drives the formation of precursor lesions and their progression to invasive PC. The Yes-associated Protein (YAP) is a transcriptional coactivator required for transformation by the RAS oncogenes and the development of PC. In Ras-driven tumors, YAP can also substitute for oncogenic KRAS to drive tumor survival after the repression of the oncogene. Ras oncoproteins exert their transforming properties through their downstream effectors, including the PI3K kinase, Rac1 GTPase, and MAPK pathways. **Methods:** To identify Ras effectors that regulate YAP, YAP levels were measured in PC cells exposed to inhibitors of oncogenic K-Ras and its effectors. **Results:** In PC cells, the inhibition of Rac1 leads to a time-dependent decline in YAP protein, which could be blocked by proteosome inhibitor MG132. This YAP degradation after Rac1 inhibition was observed in a range of cell lines using different Rac1 inhibitors, Rac1 siRNA, or expression of dominant negative Rac1^T17N^ mutant. Several E3 ubiquitin ligases, including SCF^βTrCP^, regulate YAP protein stability. To be recognized by this ligase, the βTrCP degron of YAP (amino acid 383–388) requires its phosphorylation by casein kinase 1 at Ser384 and Ser387, but these events must first be primed by the phosphorylation of Ser381 by LATS1/2. Using Flag-tagged mutants of YAP, we show that YAP degradation after Rac1 inhibition requires the integrity of this degron and is blocked by the silencing of βTrCP1/2 and by the inhibition of casein kinase 1. Unexpectedly, YAP degradation after Rac1 inhibition was still observed after the silencing of LATS1/2 or in cells carrying a LATS1/2 double knockout. **Conclusions:** These results reveal Rac1 as an oncogenic KRAS effector that contributes to YAP stabilization in PC cells. They also show that this regulation of YAP by Rac1 requires the SCF^βTrCP^ ligase but occurs independently of the LATS1/2 kinases.

## Simple Summary

The Yes-associated protein (YAP) is part of a system that regulates cell proliferation in response to changes in cell–cell and cell–matrix interactions. In malignant tumors, that system is almost always disrupted in ways that promote the stability of YAP. Mutations in genes encoding Ras proteins are found in 30% of all human cancers, but the mechanisms by which these mutated proteins promote cancer are still incompletely understood. Here, we investigated the regulation of YAP stability by the Ras oncogenes and found that while Ras does not directly regulate YAP, the stability of YAP is directly controlled by one of Ras’s own effectors, the Rac1 GTPase, a small regulatory protein involved in controlling cell shape and migration. This regulation of YAP by Rac1 could potentially play an important role in the development of therapeutic resistance to Ras inhibitors, which we know can be promoted by the overexpression of YAP.

## 1. Introduction

Over 30% of all human tumors harbor activating mutations in an *RAS* gene, predominantly *HRAS*, *KRAS*, or *NRAS*. In pancreatic cancer (PC), activating mutations in KRAS are detected in up to 90% of cases, which makes it the prototypical Ras-driven cancer [1,2,3,4,5]. PC is an insidious disease that develops silently and is often detected when it is already too late after the disease has already spread to distant tissues. Surgical resection is the only curative treatment, but less than 20% of patients are eligible as most patients present with tumors that have already spread beyond the pancreas. In mouse models of Ras-driven PC, this propensity to invade can already be seen early in the development of the disease [6]. In these models, the pancreas-specific expression of oncogenic *KRAS* is combined with the loss of a tumor suppressor to drive the formation of pancreatic intraepithelial neoplasia (PanIN) lesions and their progression to PC [7,8,9,10,11]. Applying tag and track methods to these models revealed an early ability of pancreatic tumor cells to enter the bloodstream, even before tumors can be detected [6]. The *KRAS* oncogene itself may at least in part be responsible for this invasiveness, as the oncogene has long been studied for its impacts on cell proliferation, as well as cell migration [12,13,14]. Anchored at the plasma membrane, Ras proteins serve as molecular switches involved in transducing signals from growth factor receptors to downstream pathways in the cytoplasm and nucleus [12,15]. Ras transformation impacts the actin cytoskeleton and alters actomyosin contractility in manners that promote motility and cell migration [14]. Ras proteins can exist in either an inactive GDP-bound form or an active GTP-bound state, and the ratio between these forms is dictated by the activities of GEFs (guanine exchange factors) and GAPs (GTPase activating proteins), which are both themselves regulated by growth factor receptors [12,15,16]. Once in their GTP-bound state, Ras proteins can physically interact with their downstream effectors to alter their function. Because they fail to interact with GAPs, oncogenic Ras mutants remain in their active GTP-bound state, along with constitutive activation of their downstream effector pathways. Oncogenic Ras proteins exert their transforming activities through at least four of their effector pathways: MAPK pathway (mitogen-activated protein kinase), PI3K pathway (phosphoinositide 3-kinase), Ral-GDS (Ral guanine nucleotide dissociation stimulator), and Rac1 GTPase (Ras-related C3 botulinum toxin substrate 1). Among these effectors, Rac1 GTPase is known to play a central role in cell migration, which could partly be responsible for the increased propensity of Ras-transformed cells to migrate.

Like Ras proteins, Rac1 is a membrane-bound globular protein that belongs to the GTPase family of enzymes [17]. Like Ras, Rac1 is activated by GEFs that load it with GTP in place of the GDP. As it turns out, two of the GEFs stimulating Rac1, Tiam1, and PREX1 are themselves activated by oncogenic Ras signaling [18,19,20,21]. Tiam1 possesses a Ras-binding domain (RBD) and interacts directly with GTP-bound Ras to become active. PREX1 instead possesses a Pleckstrin Homology domain, which allows it to be activated by PIP3 molecules, such as those produced by PI3K in response to GTP-bound Ras. Once bound to GTP, Rac1 interacts with its downstream effectors, many of which are involved in cell migration and pinocytosis [22,23,24,25]. During cell migration, GTP-bound Rac1 accumulates at the leading edge of lamellipodia, where it recruits the WAVE and ARP2/3 complexes to promote actin polymerization and lamellipodia formation. Activated mutants of Rac1, even more so than Ras oncogenes, promote cell motility and migration [25,26,27]. In line with its role as a downstream effector of Ras, Rac1 is required for transformation by the Ras oncogenes [28,29], as well as for the development of Ras-driven PC in mouse models [30,31]. In these models, knocking out the *RAC1* gene in the pancreas blocks acinar to ductal metaplasia (ADM) and delays the formation of PanIN lesions [31]. In this model, ADM is an early manifestation of Ras-driven tumorigenesis. In PC cell lines, levels of Rac1 and GTP-bound Rac1 are elevated compared to normal cells and tissues [32,33]. This increased activation of Rac1 likely contributes to the propensity of Ras-driven PC cells to migrate and invade.

A second pathway contributing to the invasiveness and metastasis of PC tumor cells is the Hippo/YAP pathway, one that was first described as controlling organ size during development [34,35]. The Hippo/YAP pathway plays a central role in mechanotransduction. The pathway responds to alterations in cell–cell and cell–matrix interactions. It converts these signals to changes in the function of YAP (Yes-associated protein) and TAZ (Transcriptional coactivator with PDZ-binding motif) [34,35,36,37,38,39]. Acting as transcriptional coactivators, YAP and TAZ heterodimerize with the TEAD (transcriptional enhanced associate domain) transcription factors to control the expression of genes involved in cell division, cell cycle control, and the self-renewal of stem cells [40,41,42]. The Hippo pathway also includes the LATS1 and LATS2 kinases and their upstream activators, the MST1 and MST2 kinases. Under high cell density conditions, LATS1/2 phosphorylates YAP at multiple sites, including serines S127 and S381. Phosphorylation at S127 creates a 14-3-3 binding site that sequesters YAP in the cytoplasm to block its function. Phosphorylation at S381 instead triggers the polyubiquitination and proteasomal degradation of YAP, a process mediated by the SCF^βTrCP^ E3 ubiquitin ligase [43]. YAP recognition by this ligase requires the phosphorylation of serines S384 and S387 located in the βTrCP degron of YAP (aa 383–387). These events are mediated by casein kinase 1 (CK1) but require the prior phosphorylation of serine S381 by LATS1/2 to provide a priming site for CK1 [43]. The Hippo pathway receives signals from the plasma membrane, actin cytoskeleton, and junctional complex, and these signals converge to ultimately control the LATS1/2 kinases and the function of YAP and TAZ. In primary cells, YAP overexpression suffices to overcome contact inhibition and allow anchorage-independent growth [35,44], which are two hallmarks of Ras-transformed cells. YAP is overexpressed in a wide range of malignancies, including Ras-driven PC, where its level correlates with liver metastasis and poor prognosis [45]. Like Rac1 [28,29], YAP is required for the transformation of primary cells by the Ras oncogenes [46]. Similar to Rac1 [30,31], YAP is also required for the development of tumors in mouse models of *KRAS*-driven PC [47].

Ras-driven tumors tend to become addicted to their Ras oncogene, to the extent that its subsequent repression leads to necrosis, apoptosis, and tumor regression. In mouse models of KRAS-driven PC, only a small fraction of tumors survives the repression of the oncogene [46,48,49]. In these reemerging tumors, YAP was amplified and overexpressed and YAP was needed for tumor growth in the absence of Ras. These and other studies have raised the interesting possibility that YAP might be acting downstream of oncogenic Ras as a secondary effector, one that can take over if Ras is repressed. Kolch and others have described cross-talks between the Hippo/YAP and Ras pathways and a regulation of YAP by Ras that was complex and context-dependent [36,37,50,51,52,53,54,55]. In particular, the impacts of Ras on YAP depended on the differential activation kinetics of wild-type and oncogenic K-Ras [37,54,55,56,57,58]. The epidermal growth factor (EGF) stimulation of wild-type Ras led to the AKT phosphorylation and inhibition of MST2, resulting in YAP stabilization and activation. EGF stimulation could also induce the phosphorylation of the Ajuba family protein WTIP, causing it to inhibit LATS1/2 and further stabilize YAP. In stark contrast, the chronic expression of oncogenic KRAS led to the RASSF1A-dependent activation of the MST2-LATS1 complex, resulting in decreased YAP function. Perhaps because of this negative regulation, YAP was reportedly not induced by oncogenic Ras in a mouse model of KRAS-driven lung cancer, even though it was still required for tumor formation [53]. However, in PC tumors, the wild-type and oncogenic K-Ras proteins appear to have opposite effects on Hippo/YAP signaling pathway. In PC tumors harboring only one *KRAS* mutant allele, the loss of the remaining allele accelerates disease progression and is accompanied by an increase in YAP function [59]. In this model, expression of wild-type KRAS led to S127-phosphorylation and inhibition of YAP function [59].

In this article, to better understand the regulation of YAP by oncogenic Ras and its effectors, we have exposed PC cells to inhibitors of the oncogenic K-Ras protein and inhibitors of three of its effector pathways (MAPK, PI3K, Rac1). Our results show that while YAP levels were mostly unaffected by the inhibition of oncogenic K-Ras, YAP was rapidly degraded after the inhibition of either the Rac1 GTPase or MAPK pathway. Conversely, YAP levels were increased in normal pancreatic cells by the expression of an activated mutant of Rac1. When triggered by Rac1 inhibition, YAP degradation necessitated the SCF^βTrCP^ ligase and βTrCP degron of YAP but did not require the LATS1/2 kinases. These results highlight the role of Rac1 as an upstream regulator of YAP and point to a novel Hippo-independent regulation of YAP by Rac1. The potential mechanisms involved and the implications of these findings for PC therapy are discussed.

## 2. Materials and Methods

### 2.1. Materials

Fetal bovine serum (FBS) was purchased from Atlas Biologicals (Fort Collins, CO, USA). Gentamycin, Penicillin/Streptomycin, Dulbecco’s modified Eagle’s medium (DMEM), Hygromycin B, Zeocin, and recombinant human EGF were purchased from ThermoFisher Scientific (Waltham, MA, USA). Medium M3 (cat# M3:BaseF) was from InCell Corp. (San Antonio, TX, USA). The mammalian proteases inhibitor cocktail was from Sigma–Aldrich (Saint Louis, MO, USA), and the MBS mammalian transfection kit was from Agilent (Santa Clara, CA, USA). EHT-1864, NSC23766, U0126, LY294002, MRTX1133, Ehop-016, IPA-3, FRAX597, and IC-261 were obtained from Selleck Chemicals (Houston, TX, USA). MG132 was purchased from Enzo Life Sciences (Farmingdale, NY, USA), dissolved in DMSO, and stored at −80 °C. All other chemicals were obtained from Fisher Scientific (Pittsburgh, PA, USA).

### 2.2. Cell Lines

hTERT-HPNE cells (hereby referred to as HPNE cells) and the same expressing HPV16 E6 and E7 proteins (HPNE/E6/E7 cells) were previously developed by us [60,61,62,63]. HPNE/E6/E7 cells modified to express the SV40 small t antigen and/or oncogenic K-Ras^G12D^ were also developed by us [60,61]. The AsPC1, HPAF/CD18, L3.6pl, Panc1, Hs766t, and BxPC3 cell lines used were authenticated by STR profiling performed by Genetica, LabCorp (Burlington, NC). Except for the HPNE, HPNE/E6/E7, and BxPC3 cells, all cell lines were cultivated in DMEM media supplemented with 10% Fetal Bovine Serum (FBS) and 50 μg/mL gentamycin. BxPC3 cells were cultivated in RPMI media supplemented with 10% FBS and 50 μg/mL gentamycin. All cell lines were cultivated at 37 °C in a humidified atmosphere containing 5% CO_2_. HPNE cells and their derivatives were cultivated in medium D, as before [62].

### 2.3. Drug Treatments

In a panel of human cell lines expressing K-Ras^G12D^, MRTX1133 has been reported to inhibit ERK phosphorylation with IC_50_ values ranging from 1 to 10 nM [64]. In our experiments, we exposed CD18/HPAF cells to 50 nM MRTX1133. In cells exposed to LY294002, the drug inhibited the kinase activity of P13K with an IC_50_ of 1.5 μM, as originally reported [65]. In our experiments, we have used LY294002 at a concentration of 20 μM. In NF2-deficient meningioma, where it was first characterized, FRAX597 inhibited cell proliferation with EC_50_ values ranging from 0.4 to 2.9 μM [66]. We used FRAX597 at a concentration of 5 μM. At 20 μM, IPA-3 could reportedly block the S192/S197 phosphorylation of PAK2 in human primary schwannoma cells [67]. In our experiments, IPA-3 was used at that same concentration of 20 μM. We have also used the Rac1 inhibitors NSC23766, EHT-1864, and Ehop-016 at their respective concentrations of 100 μM, 50 μM, and 20 μM, in accordance with the literature [68,69,70,71,72]. Accordingly, MEK1/2 inhibitor U0126 was used at a concentration of 50 μM, also based on the literature [73,74]. Finally, we also have treated cells with IC-261, an inhibitor of CK1δ and CK1ε [75]. The inhibitor was used at a concentration of 10 μM, in line with the published literature [76].

### 2.4. LATS1/2-KO Hela Cells

Using CRISPR/Cas9, HeLa cells were genetically modified to knock out their LATS1 and LATS2 loci. Cells were modified using plasmid pX330A_D10A-1 × 4 expressing the D10A mutant of Cas9, which possesses nickase activity. This plasmid system allows for the simultaneous co-expression of up to 7 different guide RNAs [77]. The plasmid was further modified to add the sequence of the green fluorescent protein in frame with the Cas9 open reading frame. The system was used in conjunction with the following four pairs of guide sequences targeting LATS1 and LATS2: 5′-CACCGACGCATCATAAAGCCTTGC-3′/5′-AAACGCAAGGCTTTATGATGCGTC-3′ (LATS1 exon 2, nick 1); 5′-CACCGTCTGACTTGTCGAGGATCTT-3′/5′-AAACAAGATCCTCGACAAGTCAGAC-3′ (LATS1 exon 2, nick 2); 5′-CACCGACTGCAAGAGATTCGTGAG-3′/5′-AAACCTCACGAATCTCTTGCAGTC-3′ (LATS2 exon 2, nick 1); and 5′-CACCGTTTCCAGAATAAGTCGTGGC-3′/5′-AAACGCCACGACTTATTCTGGAAAC-3′ (LATS2 exon 2, nick 2). The final DNA construct was transiently transfected into HeLa cells and GFP-positive clones were selected 48 h post-transfection by flow cytometry-based cell sorting. Single-cell clones were subsequently screened using western blotting analysis.

### 2.5. siRNA Knockdowns

Cells were reverse transfected with siRNA using DharmaFECT 1 (cat# T-2001) or DharmaFECT 3 (T-2003) according to the manufacturer’s instructions (Dharmacon, Lafayette, CO, USA). Two days later, cells were examined for expression of the knocked-down targets (Rac1, βTrCP1, βTrCP2, LATS1, LATS2, and Skp1) and for differences in YAP levels and response to Rac1 inhibition. ON-TARGETplus siRNA was purchased from Dharmacon (Lafayette, CO, USA), including the non-targeting control pool (cat# D-001810-10) and SMARTpools against human Rac1 (cat# L-003560-00), SKP1 (cat# L-003323-00), BTRC (i.e., βTrCP1; cat# L-003463-00), FBXW11 (i.e., βTrCP2; cat# L-003490-00), LATS1 (cat# L-004632-00), or LATS2 (cat# L-003865-00).

### 2.6. Adenoviral Vectors and Adenoviral Infections

Recombinant adenovirus N17Rac1 (Ad.N17Rac1) and control adenovirus dl312 (Ad.CTR) were kindly provided by Dr. Toren Finkel (NIH, Bethesda, MD, USA). In Ad.N17Rac1, the Rac1 cDNA contains a Ser to Asp substitution at position 17 and functions as a dominant negative mutant [78]. For 24 h, log-phase HPNE and HPNE/E6/E7 cells were exposed to either the Ad.N17Rac1 or Ad.CTR viruses at 50 pfu/cell, as described previously [79]. After 16 h, cells were given fresh medium D and cultivated until the next day before being photographed and harvested for analysis.

### 2.7. Retroviral Vectors

Retroviruses made from vectors pLXSH-Flag-YAP(wt) and pLXSH-Flag-YAP(5SA) were used to infect Panc1 cells to make them express the wild-type Flag-YAP protein and its 5SA mutant, respectively. The two vectors were made by insertion of an Sal1/Bgl2-digested PCR product into the Xho1/BamH1 sites of vector pLXSH carrying Hygromycin resistance. PCR products encoding Flag-tagged YAP and its 5SA mutant were respectively amplified from vectors p2xFlag CMV2-YAP2 (a gift from Marius Sudol’s lab; purchased from Addgene cat# 19045) and pCMV2-flag YAP2 5SA (a gift from Kunliang Guan’s lab; purchased from Addgene cat# 27371) using the same forward (5′-GTACGCGTCGACAGTGAACCGTCAGAATTGATCTA-3′; Sal1 site underlined) and reverse (5′-CATGGAAGATCTCTATAACCATGTAAGAAAGCTT-3′; Bgl2 site underlined) primers. The amino acid sequence of the 5SA mutant differs from that of wild-type YAP by the presence of mutations in all of its LATS1/2 phosphorylation sites (S61A, S109A, S127A, S128A, S131A, S163A, S164A, and S381A). Retroviral vector pBabeBleo-Flag-BRAF(V600E) expressing the BRAF oncogene along with Zeocin resistance, a gift from Christopher Counter’s lab [80], was purchased from Addgene (cat# 53156; Watertown, MA, USA). Retroviral vector pLXSH-myc-Rac1(G12V) was made by site-directed mutagenesis of plasmid pLXSH-myc-Rac1(wt) encoding a myc-tagged version of human Rac1, which we describe below. The mutation was introduced using the QuikChange Site-Directed Mutagenesis Kit (Agilent, Santa Clara, CA, USA) with oligonucleotides 5′-GTGGTGGTGGGAGACGTAGCTGTAGGTAAAAC-3′ and 5′-GTTTTACCTACAGC-TACGTCTCCCACCACCAC-3′ (codon 12 underlined). Retroviral vector pLXSH-myc-Rac1(wt) was made by the insertion of a Msc1/Xho1-digested PCR product into the Hpa1/Xho1 sites of pLXSH. The PCR product encoding myc-tagged Rac1 had been amplified from vector pRK5-myc-Rac1-wt (a gift from Gary Bokoch’s lab; purchased from Addgene cat# 12985) using forward primer 5′-TGGAAGATCTGGCCACCATGGAGCA-GAAG-3′ (Msc1 site underlined) and reverse primer 5′-TGGCCTCGAGATAAACAAGTTGGGCCATGGC-3′ (Xho1 site underlined). All vectors were validated by the Sanger sequencing of their open reading frame from both directions.

### 2.8. Site-Directed Mutagenesis of pLXSH-Flag-YAP(wt)

The D383A/S384A, S351A/P352A, and S384A mutants of Flag-YAP were produced by site-directed mutagenesis of plasmid pLXSH-Flag-YAP(wt) described above. Mutations were introduced using the QuikChange Site-Directed Mutagenesis Kit following the manufacturer’s instruction (Agilent, Santa Clara, CA, USA). The following oligonucleotides were used to respectively create the D383A/S384A mutation (5′-GAGATGAGAGTACAGCCGCTGGACTAAGCATGAG-3′ and 5′-CTCATGCTTAGTCCAGCGGCTGTAC-TCTCATCTC-3′; Codons 383/384 underlined), S351A/P352A mutation (5′-CAAAATCCAGTGTCTGCTGCCGGGATGTCTCAGG-3′ and 5′-CCTGAGACATCCCGGCAGCAGACACTGGATTTTG-3′; Codons 351/352 underlined), and S384A mutation (5′-GATGAGAGTACAGACGCTGGACTAAGCATGAG-3′ and mut-S384A-R 5′-CTCATGCTTAGTCCAGCGTCTGTACTCTCATC-3′; codon 384 underlined). All vectors were validated by the Sanger sequencing of their open reading frame from both directions.

### 2.9. Retroviral Production and Transductions

Retroviral particles were produced in Phoenix-AMPHO cells (CRL-3213; ATCC, Manassas, VA, USA) transiently transfected with the different retroviral vectors using the MBS mammalian transfection kit (Agilent, Santa Clara, CA, USA). Viral supernatants were produced in medium D (to infect the HPNE lines) or in DMEM supplemented with 10% FBS (to infect Panc1 cells). Supernatants were cleared through 0.45 μm filters, supplemented with 4 ug/mL polybrene, and stored frozen at −80 °C. In 6-well plates, exponentially growing HPNE and HPNE/E6/E7 cells were trice transduced with 2 mL of viral supernatant each time from the pLXSH, pLXSH-Flag-YAP(wt), or else pBabeBleo-Flag-BRAF(V600E) vector. Infected cells were selected for viral integration using either 200 μg/mL Hygromycin B (for the pLXSH-derived vectors) or else 50 μg/mL Zeocin (for the pBabeBleo vector). After 10 days of selection, cells were monitored for differences in proliferation rates, and cells were set aside for western blot analysis and for quantification of SA-β-galactosidase activity. A similar approach was employed to establish the Panc1 cell lines expressing the different Flag-YAP proteins (wild-type, D383A/S384A, S351A/P352A, and S384A) or empty vector (pLXSH), again using Hygromycin 200 μg/mL to select for viral integration.

### 2.10. SA-β-galactosidase Activity

Cells were stained for the presence of SA-β-galactosidase activity as we have previously carried out [81].

### 2.11. Western Blot Analysis

Western blot analyses were performed as previously described [82]. Briefly, cells plated in 35 mm dishes and treated with different drugs or siRNA were recovered in 200 µL of Laemmli buffer, denatured (95 °C × 5 min), and stored at −20 °C. Samples were separated by SDS-PAGE on 4–20% polyacrylamide gels (Bio-Rad, Hercules, CA, USA). Equal volumes of samples were loaded per lane or else proteins were quantified using the RC DC^TM^ protein assay kit (Bio-Rad, Hercules, CA, USA), and equal amounts of proteins were loaded (100 µg/lane). Antibodies against GAPDH (cat# sc-47724), β-actin (cat# sc-1616), ERK (cat# sc-154-G), p-ERK(T202/Y204)(cat# sc-7383), and KPM (i.e., LATS2; cat# sc-515579) were from Santa Cruz Biotechnology (Dallas, TX, USA). Rabbit monoclonal antibodies against S127-phosphorylated YAP (clone D9W21; cat# 13008), S397-phosphorylated YAP (clone D1E7Y; cat# 13619), LATS1 (clone C66B5; cat# 3477), and p16 (clone D7C1M; cat# 80772) were from Cell Signaling Technology (Danvers, MA, USA). Also from Cell Signaling Technology were the polyclonal rabbit antibody against Skp1 (cat# 2156), mouse monoclonal antibodies against YAP (clone 1A12; cat# 12395), and the Myc tag (clone 9B11; cat# 2276). Mouse monoclonal antibody against Rac1 (clone 23A8; cat# 05-389) was from Millipore–Sigma (Saint Louis, MO, USA). The Flag-tagged proteins were detected using either the mouse monoclonal M2 antibody (cat# F1804; Millipore–Sigma, Saint Louis, MO, USA) or the rat monoclonal anti-DYKDDDDK antibody (cat# 200474; Agilent, Santa Clara, CA, USA). Secondary antibodies used were horseradish peroxidase-conjugated goat antibodies against mouse, rat, or rabbit IgG (Jackson ImmunoResearch; West Grove, PA, USA). Size markers used were the Precision Plus Protein™ Dual Color Standards (cat# 1610374) from Bio-Rad (Hercules, CA, USA). Densitometry readings of intensity signals were quantified with the help of the ImageJ program (Version 1.50i) (NIH; Bethesda, MD, USA).

### 2.12. Rac1 Pulldown Assay

GST-PAK1 pull-down assays to measure the level of GTP-bound Rac1 (Rac1-GTP) were performed as previously described [33]. HPAF/CD18 extracts were used as positive controls and the same extracts pre-incubated with GDP were used as negative controls.

### 2.13. Statistical Analysis

In Figure 2A, a Spearman’s rank correlation test was utilized to assess the association between Rac1 and YAP protein levels across a panel of eight pancreatic cell lines. The association was determined to be statistically significant with a Spearman’s correlation coefficient of 0.95238 and a *p*-value of 0.00026 (*n* = 8). In Figure 2C, to compare the three cell populations of each experiment, ANOVA was first used with a *p*-value of 0.05, followed by pairwise Student’s *t*-tests conducted according to the Bonferroni method. In both experiments, the percentage of senescent cells in the BRAF^V600E^-transfected cells was higher than in the other populations, and these differences were statistically significant (Student’s *t*-test; *p* < 0.001).

## 3. Results

### 3.1. YAP Levels in PC Cells Are Unaffected by the Inhibition of Oncogenic K-Ras

**Figure 1:**
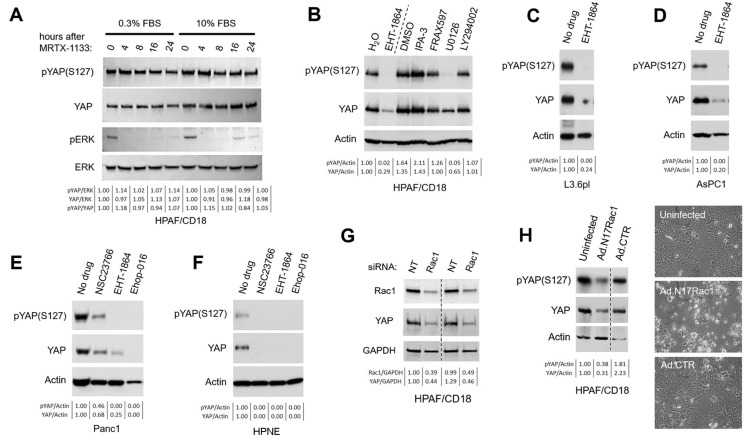
YAP levels are regulated by Rac1. (**A**) Impacts of oncogenic K-Ras^G12D^ inhibition on levels of YAP in PC cells. HPAF/CD18 cells, known to express oncogenic K-Ras^G12D^, were treated with 50 nM MRTX1133 for the indicated times. The experiment was performed in medium supplemented with either 10% FBS or 0.3% FBS. Levels of pERK, YAP, and S127-phosphorylated YAP were measured by immunoblotting. Total ERK was used as a loading control. (**B**) YAP levels in PC cells treated with inhibitors of oncogenic Ras effectors. HPAF/CD18 cells were treated for 16 h with inhibitors of the MAPK pathway (50 μM U0126), PI3K kinase (20 μM LY294002), Rac1 GTPase (50 μM EHT-1864), and PAK1-3 kinases (5 μM FRAX597; 20 μM IPA-3). Levels of YAP and S127-phosphorylated YAP were measured by immunoblotting. Actin was used as a loading control. (**C**–**F**) YAP levels in a panel of human pancreatic cell lines treated with different Rac1 inhibitors. The indicated cell lines were treated with EHT-1864 (50 μM), NSC23766 (100 μM), and/or Ehop-016 (20 μM). Sixteen hours later, levels of YAP and S127-phosphorylated YAP were measured. Actin was used as an internal standard. (**G**) The siRNA-mediated knockdown of Rac1 reduces YAP levels in PC cells. In duplicate, HPAF/CD18 cells were transfected with Rac1 siRNA (Rac1) or with a non-targeting siRNA (NT). Two days later, levels of Rac1 and YAP were measured. GAPDH was used as an internal standard. Densitometry readings for the intensity ratios of Rac1/GAPDH and YAP/GAPDH, with the value of the first NT-transfected replicate arbitrarily set to 1. The dotted line indicates a lane that was spliced out of the raw imaging data. As shown in the supplement, the two sides of the dotted line are from the same exposure of the same blot. (**H**) Expression of dominant negative Rac1^T17N^ mutant reduces YAP levels in PC cells. HPAF/CD18 cells were infected for 24 h with adenoviral particles carrying no insert (Ad.CTR; 50 pfu/cell) or expressing Rac1^T17N^ (Ad.N17Rac1; 50 pfu/cell). Forty-eight hours post-infection, levels of YAP were quantified using western blotting. Actin was used as an internal standard. The right panel shows representative images of the infected cells at 48 h post-infection (100× magnification). The dotted line indicates a lane that was spliced out of the raw imaging data. As shown in the supplement, the two sides of the dotted line are from the same exposure of the same blot. For all panels, the relevant densitometry readings for the indicated intensity ratios (pYAP/ERK, YAP/ERK, pYAP/YAP, pYAP/Actin, YAP/Actin, Rac1/GAPDH, and YAP/GAPDH) are shown below the lanes of each western blot.

We tested the effects of inhibiting the G12D mutant of KRAS in the PC cell line carrying these mutants, including both HPAF/CD18 and Panc1 cells. For this purpose, we have exposed these cells to the G12D-specific inhibitor of oncogenic K-Ras MRTX1133 [64]. In HPAF/CD18 cells, MRTX1133 led to the rapid inhibition of MAPK signaling, within 4 h of exposure, as indicated by the loss of pERK (Figure 1A). However, levels of neither YAP nor S127-phosphorylated YAP were affected by the inhibition of oncogenic Ras, even after more than 36 h of exposure to MRTX1133 (Figure 1A; 10% FBS). To make sure that Ras signaling was completely inhibited, including from the wild-type Ras molecules, exposure to MRTX1133 was combined with serum starvation (Figure 1A; 0.3% FBS). Even under these conditions, MRTX1133 did not cause YAP levels to decline or otherwise be altered. Similar results were observed in Panc1 cells (Figure S1). In this line also, MTRX1133 treatment had little impact on levels of YAP or S127-phosphorylated YAP. 

These results show that in PC cells, YAP levels are not directly regulated by the signaling activity of oncogenic K-Ras proteins.

### 3.2. YAP Levels Are Co-Regulated by the MAPK Pathway and Rac1 GTPase

The oncogenic K-Ras protein signals through at least three downstream effector pathways that play distinct roles in malignant transformation: MAPK, PI3K, and Rac1 pathways. To identify Ras effector pathways that regulate YAP levels in PC tumors expressing oncogenic K-Ras^G12D^, HPAF/CD18 cells were exposed to inhibitors of the MAPK pathway (U0126), PI3K pathway (LY294002) [65,83], and Rac1 GTPase (EHT-1864) [68,69]. Also tested were inhibitors of the PAK1-3 kinases, which are known to serve as effectors of Rac1, including IPA-3 [67,84,85] and FRAX597 [66,86]. After 16 h of exposure, cells were analyzed for changes in YAP levels and S127-phosphorylated YAP (Figure 1B). Levels of both proteins were significantly reduced by both the U0126 and EHT-1864, but not inhibitors of the PI3K or PAK1-3 kinases. In dose–response experiments, EHT-1864 reduced levels of YAP with EC_50_ values (14 ± 4 μM; Figure S2) consistent with the reported literature for this drug [68].

The MAPK pathway has previously been reported to regulate YAP function and stability, with several mechanisms already proposed [37,54,55,56,57]. In contrast, very little had been published on the regulation of YAP by Rac1, aside from one study implicating the β1-integrin/Rac1 pathway in the regulation of YAP [87]. For this reason, we purposely focused the rest of our work on the regulation of YAP by Rac1. In follow-up experiments, we exposed additional PC cell lines to EHT-1864 and other structurally unrelated Rac1 inhibitors, including Ehop-016 [70] and NSC23766 [71,72]. As in the HPAF/CD18 cells, EHT-1864 also reduced YAP levels in L3.6pl (Figure 1C), AsPC1 (Figure 1D), and Panc1 cells (Figure 1E). In Panc1 cells, YAP levels were also reduced by Ehop-016 and NSC23766. HPNE cells are human primary pancreatic ductal cells immortalized by us using telomerase [62,63]. The cells have an intact karyotype and normal growth properties, and express wild-type K-Ras, p53, and p16 [61,63]. In these normal cells, YAP levels were also reduced by the same three Rac1 inhibitors (Figure 1F).

To further validate Rac1 as an upstream regulator of YAP, HPAF/CD18 cells were transfected with Rac1 siRNA and were infected with an adenoviral vector expressing Rac1^T17N^, a dominant negative mutant of Rac1 [78,88]. In Figure 1G, the siRNA-mediated knockdown of Rac1 led to a 55% decrease in YAP levels compared to the control cells transfected with a non-targeting siRNA. Similar results were observed in HPAF/CD18 cells infected with adenoviruses expressing Rac1^T17N^. As Figure 1H indicates, levels of both total YAP and S127-phosphorylated YAP were reduced by 65% after cells were infected with the Rac1^T17N^ virus (Ad.N17Rac1) compared to the mock-infected cells or those infected with the control virus (Ad.CTR). The Rac1^T17N^ mutant also had an impact on cell morphology, causing the cells to contract and ball up (Figure 1H; right panel). Taken together, these results show that YAP levels drop in human pancreatic cells when Rac1 signaling is disrupted by pharmacological (EHT-1864, NSC23766, Ehop-016) or biological (Rac1^T17N^, Rac1 siRNA) inhibitors of Rac1 signaling.

### 3.3. YAP and Rac1 Are Co-Expressed Across a Panel of Pancreatic Cell Lines

**Figure 2:**
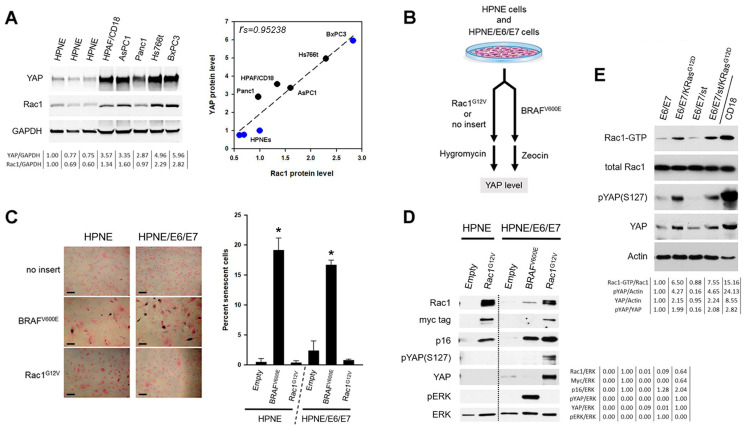
Activated Rac1 mutant cooperates with E6 and E7 to up-regulate YAP levels. (**A**) Levels of YAP and Rac1 in a panel of PC cell lines and HPNE cells. Levels of the two proteins were quantified by immunoblotting in the indicated cell lines. GAPDH was used as an internal standard. The graph on the right correlates the protein levels of YAP and Rac1. The Spearman’s correlation coefficient (r_s_) is shown. Cell lines with wild-type KRAS are labeled with a blue circle and those with oncogenic KRAS have the black circles. (**B**) Transduction of HPNE and HPNE/E6/E7 cells with activated forms of Rac1 and BRAF. HPNE cells (Puro^R^) and HPNE/E6/E7 cells (Puro^R^, Neo^R^) were infected with retroviral particles carrying no insert (pLXSH, Hygro^R^) or expressing oncogenic mutants of either BRAF (pMSCV-BRAF^V600E^, Zeocin^R^) or Rac1 (pLXSH-Myc-Rac1^G12V^, Hygro^R^). After 10 days of selection for viral integration, cells were examined for differences in YAP levels and markers of senescence. (**C**) Oncogenic BRAF triggers oncogene-induced senescence in HPNE cells. In triplicates, selected cells were plated at low density, histochemically stained to reveal SA-β-galactosidase activity, and then counter-stained with eosin. Representative images of the stained cells are shown on the left. Scale bars are 200 μm in length. The right graph shows the percentage of senescent cells in each population expressed as a mean +/− S.D. (*n* = 3). * Statistically different from the other two populations in a Student’s *t*-test with *p* < 0.001. (**D**) Activated Rac1 cooperates with E6/E7 to elevate YAP levels in PC cells. Selected cell populations were analyzed by immunoblotting with the indicated antibodies. GAPDH was used as an internal standard. The dotted line indicates a lane that was spliced out of the raw imaging data. As shown in the supplement, the two sides of the dotted line are from the same exposure of the same blot. (**E**) YAP levels are slightly elevated in HPNE derivatives expressing oncogenic K-Ras^G12D^. HPNE/E6/E7 and HPNE/E6/E7/st cells and the same expressing oncogenic K-Ras^G12D^ were analyzed for differences in levels of YAP and S127-phosphorylated YAP. Using a GST-PAK1 pulldown assay, levels of GTP-bound Rac1 (GTP-Rac1) were also measured, along with total Rac1 levels. Actin was used as an internal standard. For all panels, the relevant densitometry readings for the indicated intensity ratios (YAP/GAPDH, Rac1/GAPDH, Rac1/ERK, myc/ERK, p16/ERK, pYAP/ERK, YAP/ERK, pERK/ERK, pYAP/YAP, Rac1-GTP/Rac1, pYAP/Actin, and YAP/Actin) are shown below the lanes of each western blot.

The YAP [47,89] and Rac1 [31,33,90,91] proteins were separately reported to be up-regulated in pancreatic cancer (PC) specimens. Here, we measured levels of YAP and Rac1 together across a small panel of PC cell lines and three different lots of normal HPNE cells. As Figure 2A shows, a positive correlation between YAP and Rac1 levels was observed across the panel. In a Spearman’s rank test, the association was determined to be statistically significant with a Spearman’s correlation coefficient of 0.95238 and a *p*-value of 0.00026 (*n* = 8). The lowest levels of Rac1 and YAP were detected in three independent lots of normal HPNE cells, and the highest levels were observed in the HPAF/CD18, AsPC1, Hs766t, and BxPC3 cell lines. Intermediary levels of the two proteins were seen in Panc1 cells, known to have reduced tumorigenicity among PC cell lines [92]. There was no correlation between the mutational status of KRAS and the levels of either Rac1 or YAP (Figure 2A; black versus blue circles).

### 3.4. Oncogenic Rac1 Up-Regulates YAP in Normal HPNE Cells

We and others have used HPNE cells as a system to study and reconstitute the early stages of pancreatic cancer development, in particular, the activation of KRAS [60,61,62,63,93,94]. In normal cells, such as HPNE cells, oncogenic KRAS expression triggers oncogene-induced senescence, an irreversible cell cycle arrest mediated by the p16/RB and p53/p21 pathways. Overcoming this response to allow transformation by Ras can be achieved with viral oncoproteins that block RB and p53, such as the E6 and E7 proteins of HPV16 [60,61]. Oncogene-induced senescence can be triggered by oncogenic mutants of KRAS [95], HRAS [96], NRAS [97], and BRAF [98], among others. To determine if senescence is also induced by activated mutants of Rac1 and to investigate the regulation of YAP by Rac1, HPNE cells and the same expressing E6/E7 (HPNE/E6/E7 cells) [60,61] were infected with retroviruses expressing Rac1^G12V^, an activated mutant of Rac1 [28]. Viruses carrying no insert or expressing an activated mutant of BRAF (BRAF^V600E^) [98] were, respectively, used as negative control and positive control for oncogene-induced senescence (OIS). After selection for viral integration (Figure 2B), cell populations were compared for differences in YAP levels (Figure 2D) and markers of senescence (Figure 2 2C,D). As expected, HPNE cells infected with the BRAF oncogene experienced OIS, as noted by their induction of SA-β-galactosidase activity (Figure 2C), enlargement/flattening (Figure 2C), and failure to divide. Unexpectedly, this BRAF-induced senescence could not be blocked by the E6 and E7 proteins and was still induced in the HPNE/E6/E7 cells (Figure 2C). No evidence of increased SA-β-galactosidase activity was noted in either cell lines after infection with the empty virus or the Rac1^G12V^ virus. The selected cell populations were all subjected to western blot analysis, except for the BRAF^V600E^-infected HPNE cells which did not yield sufficient material. Overexpression of the myc-tagged Rac1^G12V^ protein was detected with antibodies against Rac1 or the myc tag (Figure 2D). Expression of BRAF^V600E^ was instead confirmed by its supra-physiological activation of the ERK1/2 kinases (pERK; Figure 2D). Levels of YAP and S127-phosphorylated YAP are low in HPNE cells and could barely be detected across the five samples, except for the HPNE/E6/E7 cells infected with the Rac1^G12V^ virus, where 11-fold higher levels of YAP were detected (Figure 2D). We also monitored p16, a marker of OIS [96]. As expected, p16 was induced by the expression of the BRAF oncogene (Figure 2D), in line with the induction of SA-β-galactosidase by the oncogene (Figure 2C). Similar to BRAF^V600E^, expression of the Rac1^G12V^ also resulted in the up-regulation of p16 (Figure 2D), albeit without the concomitant induction of SA-β-galactosidase (Figure 2C). In HPNE cells, expression of Rac1^G12V^ did reduce proliferation rates but without causing the cells to become quiescent (0.79 ± 0.03 versus 1.95 ± 0.02 doublings/day; *n* = 3). In HPNE/E6/E7 cells, expression of Rac1^G12V^ did the opposite and led to higher rates of proliferation (1.77 ± 0.04 versus 1.75 ± 0.04 doublings/day; *n* = 3). Thus, the activated Rac1 mutant can cooperate with the E6/E7 proteins to up-regulate YAP levels in HPNE cells, and it can do so without the induction of OIS.

In Figure 2E, we repeated these experiments with oncogenic K-Ras^G12D^ instead of the activated Rac1 mutant. For this purpose, we have measured the steady-state levels of YAP and S127-phosphorylated across a panel of HPNE cells expressing E6/E7 along with oncogenic K-Ras^G12D^ and/or the SV40 small t antigen. Among the four lines, only the HPNE/E6/E7/st/K-Ras^G12D^ are transformed and able to grow in soft and form tumors in mice [61]. Two pairs of HPNE cell derivatives were compared to reveal the impact of oncogenic KRAS on YAP levels. In the first pair, HPNE/E6/E7 cells were compared to the same but modified to express KrasG12D (HPNE/E6/E7 vs. HPNE/E6/E7/K-Ras^G12D^ cells). In the second pair, we instead compared the HPNE/E6/E7/st cells with the same but modified to express KrasG12D (HPNE/E6/E7/st vs. HPNE/E6/E7/st/K-Ras^G12D^ cells). A day prior to harvesting, the four lines cells were fed medium containing 0.3% fetal bovine serum to reduce endogenous wild-type Ras signaling so as to reveal the effects of constitutively activated K-Ras^G12D^ on YAP levels. In both pairs of cell lines, cells expressing K-Ras^G12D^ had total YAP levels that were approximately two-fold higher than their respective controls (Figure 2E). These higher levels of YAP were intermediary between those of the unmodified HPNE/E6/E7 cells and those of the HPAF/CD18 cells, also known to express the K-Ras^G12D^ protein. The SV40 small t antigen did not appear to affect YAP levels, given that its levels were the same between the E6/E7 and E6/E7/st cells, as well as between the E6/E7/K-Ras^G12D^ and E6/E7/st/K-Ras^G12D^ cells. Using a GST-PAK pulldown assay, we have measured the level of active GTP-bound Rac1 in the same four HPNE lines (Figure 2E; Rac1-GTP). In this assay, the GTP-bound Rac1 is selectively captured by the GST-PAK1 fusion proteins, so its level can be quantified using western blotting. In both the HPNE/E6/E7 and HPNE/E6/E7/st cells, the expression of oncogenic K-Ras^G12D^ led to six to eight-fold higher levels of active Rac1. These results show that in HPNE cells expressing E6/E7 or E6/E7/st, oncogenic K-Ras^G12D^ expression modestly increases the steady-state levels of YAP and S127-phosphorylated YAP. They also serve to validate Rac1 as a downstream effector of oncogenic K-Ras^G12D^.

### 3.5. YAP Degradation After Rac1 Inhibition Requires the SCF^βTrCP^ E3 Ubiquitin Ligase

**Figure 3:**
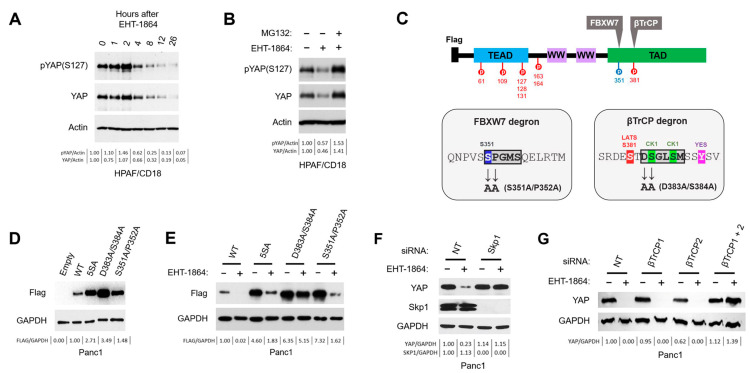
YAP degradation after Rac1 inhibition requires the SCF^βTrCP^ E3 ubiquitin ligase. (**A**) Time course of YAP decline after Rac1 inhibition. HPAF/CD18 cells were harvested at the indicated time point after the addition of EHT-1864 (50 μM). Levels of YAP and S127-phosphorylated YAP were quantified by immunoblotting. GAPDH was used as an internal standard. (**B**) MG132 blocks the degradation of YAP elicited by the inhibition of Rac1. After 12 h of exposure to EHT-1864 (50 μM; +) or vehicle control (−), HPAF/CD18 cells were exposed or not to MG132 (20 μg/mL) for 4 h prior to harvesting. (**C**) Schematic description of YAP structure showing the position and sequence of critical degrons and phosphorylation sites. Upper drawing shows the structure of YAP, including its heterodimerization domain (TEAD), WD40 domains (WW), transactivation domain (TAD), putative degrons (βTrCP1/2, FBXW7), and LATS1/2 phosphorylation sites (S61, S109, S127, S128, S131, S163, S164, and S381). Lower left panel: Sequence of the putative FBXW7 phosphodegron highlighting its consensus and its potentially required phosphoserine group (S351). Changes introduced by the S351A/P352A mutation are also shown. Lower right panel: Sequence of the βTrCP degron of YAP highlighting its consensus and its required CK1 (S384, S387) and LATS1/2 (S381) phosphorylation sites. Changes introduced by the D383A/S384A mutation are also shown. (**D**) Detection of the Flag-YAP proteins in retrovirally infected Panc1 cells. Panc1 cells infected with pLXSH viruses carrying no insert (Empty), Flag-tagged YAP (WT), or its various mutant versions (5SA, D383A/S384A, S351A/P352A) were analyzed for the presence of Flag-YAP. GAPDH was used as an internal control. (**E**) The βTrCP degron is needed for YAP degradation after Rac1 inhibition, but not the S381 LATS1/2 phosphorylation site. In duplicate, Panc1 cells expressing the different mutants of Flag-YAP were exposed to EHT-1864 (50 μM). Sixteen hours later, Flag-tagged proteins were quantified using western blotting. Again, GAPDH was used as an internal control. (**F**) The siRNA-mediated knockdown of Skp1 blocks YAP degradation after Rac1 inhibition. Panc1 cells were transfected with skp1 siRNA or with a non-targeting siRNA. Forty-eight hours later, transfected cells were treated with EHT-1864 (50 μM; +) or vehicle control (−) for 16 h prior to western blot analysis. (**G**) The siRNA-mediated knockdown of the βTrCP1/2 proteins blocks YAP degradation after Rac1 inhibition. Panc1 cells were transfected with a non-targeting siRNA or with siRNA against βTrCP1, βTrCP2, or both proteins. Forty-eight hours later, transfected cells were treated with EHT-1864 (50 μM; +) or vehicle control (−) for 16 h prior to western blot analysis. GAPDH was again used as an internal standard. For all panels, densitometry readings for the indicated intensity ratios (pYAP/GAPDH, YAP/GAPDH, Skp1/GAPDH, and FLAG/GAPDH) are shown below the lanes of each western blot.

YAP levels are principally regulated at the protein stability level by the ubiquitin-proteasome system (UPS) [43]. Figure 3A shows the time course of YAP protein decline in HPAF/CD18 cells treated with EHT-1864. YAP levels remain stable until two hours after EHT-1864 and then decline precipitously. To determine the involvement of UPS in this decline, cells were pre-treated with proteasome inhibitor MG132 prior to EHT-1864 exposure. As the results show, pre-treating HPAF/CD18 cells with MG132 could prevent the decline in YAP protein normally observed at 12 h after EHT-1864 (Figure 3B and Figure S3). These results show that the loss of YAP proteins observed after the inhibition of Rac1 is mediated by UPS and its 26S proteasome.

The YAP protein carries a phosphodegron recognized by the βTrCP1 and βTrCP2 proteins (Figure 3C; βTrCP degron) [43]. These proteins serve as substrate adaptors for an SCF^βTrCP^ E3 ubiquitin ligase (Skp1-Cul1-F-box) that targets YAP for polyubiquitination and proteolysis [43]. This degron contains a DSGMS motif that must be phosphorylated at its two serine residues to be recognized by βTrCP1/2 (Figure 3C; βTrCP degron; S384 and S387 in green). This phosphorylation is accomplished by casein kinase 1 (CK1) but requires the prior phosphorylation of serine S381 by the LATS1/2 kinases (Figure 3C; βTrCP degron; S381 in red) [43]. This prior event, which represents the rate-limiting step, is needed to create a priming site for CK1 [43]. Through bioinformatic analysis, we also found a ^351^SPGMS^355^ motif with similarities to the degron of another E3 ubiquitin ligase, SCF^FBXW7^ (Figure 3C; FBXW7 degron). Its substrate adaptor, FBXW7, reportedly binds to a (S/T)Pxx(S/T/E/H) motif when its first serine/threonine residue is phosphorylated (underlined; x is any residue) [99]. To define the roles played by the SCF^βTrCP^ and SCF^FBXW7^ ligases and the LATS1/2 kinases in the regulation of YAP by Rac1, PC cells were modified to express three different mutants of a Flag-tagged YAP protein. These mutants included the 5SA mutant of YAP designed to lack all of its LATS1/2 phosphorylation sites [44], among which was the S381 site that controls activation of the βTrCP degron. A second mutant, designed by us, was created to directly inactivate the βTrCP degron by changing its central DS motif to AA (D383A/S384A; Figure 3C), a change that blocks its recognition by the βTrCP1/2 proteins [43]. Finally, to inactivate the putative FBXW7 degron, its most conserved SP positions were changed to AA (S351A/P352A; Figure 3C). These three mutants were then compared to the wild-type Flag-YAP protein for differences in their respective responses to Rac1 inhibition.

Panc1 cells were infected with retroviruses expressing the different Flag-tagged YAP proteins. After selection for viral integrations, cells were analyzed using western blot to confirm the expression of the Flag-YAP proteins. Consistent with the notion that they carry stabilizing mutations, the three YAP mutant proteins (5SA, D383A/S384A, and S351A/P352A) were expressed at higher levels compared to the wild-type YAP control (Figure 3D). To assess the response of each Flag-YAP protein to Rac1 inhibition, Panc1 cells expressing each were treated with EHT-1864 for 16 h. DMSO vehicle was used as a vehicle control. As Figure 3E shows, EHT-1864 led to a marked reduction in wild-type Flag-YAP, by more than 10-fold. Similar declines were observed for the 5SA and S351A/P352A mutants but not for the D383A/S384A mutant (Figure 3E). Instead, levels of the D383A/S384A mutant were only minimally affected by the inhibition of Rac1. These results show that the integrity of the βTrCP degron is required for YAP degradation after Rac1 inhibition but not the S381 phosphorylation site, which the 5SA mutant lacks. This finding was unexpected because the activation of this degron also reportedly requires the phosphorylation of S381 by the LATS1/2 kinases [43].

To further confirm the involvement of the SCF^βTrCP1/2^ ligase in the regulation of YAP by Rac1, components of this ligase were depleted in Panc1 cells with siRNA against Skp1, βTrCP1, and/or βTrCP2. In Figure 3F, Panc1 cells were transfected with Skp1 siRNA (Skp1) or non-targeting siRNA (NT). Two days later, cells were then exposed to EHT-1864 or else the DMSO vehicle, with samples collected 24 h later. As Figure 3F shows, the decline in YAP protein elicited by the Rac1 inhibitor could entirely be blocked by the complete knockdown of Skp1. Next, we investigate the contribution of the βTrCP1 and βTrCP2 proteins. Panc1 cells were instead transfected with siRNA against βTrCP1, βTrCP2, or both proteins. A non-targeting siRNA was used as control. Two days post-transfection, cells were treated with EHT-1864 or else the DMSO vehicle. As Figure 3G shows, the simultaneous silencing of βTrCP1 and βTrCP2 could completely block the decline in YAP protein induced by EHT-1864, but not the silencing of one βTrCP protein alone or the other. These results confirm that the SCF^βTrCP1/2^ E3 ubiquitin ligase is indeed required for YAP degradation after Rac1 inhibition.

### 3.6. YAP Degradation After Rac1 Inhibition Occurs Independently of the LATS1/2 Kinases

**Figure 4:**
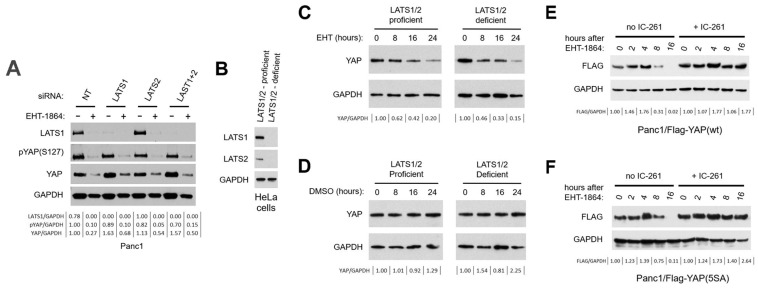
YAP degradation after Rac1 inhibition is LATS1/2-independent but requires CK1. (**A**) The silencing of LATS1/2 fails to prevent YAP degradation after Rac1 inhibition. Panc1 cells were transfected with a non-targeting siRNA or with siRNA against LATS1, LATS2, or both proteins. Forty-eight hours later, transfected cells were treated with EHT-1864 (50 μM; +) or the vehicle control (−) for 16 h prior to western blot analysis. GAPDH was again used as an internal standard. (**B**) Detection of LATS1 and LATS2 in the LATS1/2-proficient and -deficient HeLa cells. Cells were probed with the indicated antibodies. (**C**,**D**) LATS1/2 are not needed for YAP degradation after Rac1 inhibition. LATS1/2-proficient and -deficient cells HeLa cells were exposed to 50 μM EHT-1864 (**C**) or the DMSO vehicle (**D**) for 16 h, after which YAP levels were measured. (**E**,**F**) Panc1 cells expressing the Flag-YAP protein (**E**) or its 5SA mutant (**F**) were exposed or not to CK1 inhibitor IC-261 (10 μM), after which YAP levels were measured. GAPDH was used as an internal standard. For all panels, densitometry readings for the indicated intensity ratios (pYAP/GAPDH, YAP/GAPDH, LATS1/GAPDH, and FLAG/GAPDH) are shown below the lanes of each western blot.

Next, we re-examined the role played by the LATS1/2 kinases in the regulation of YAP by Rac1. In the first series of experiments, Panc1 cells were transfected with siRNA against LATS1, LATS2, or both kinases. A non-targeting siRNA was used as control (NT). Two days post-transfection, cells were again treated with EHT-1864 or else the DMSO vehicle. As Figure 4A shows, LATS1 was efficiently depleted by its siRNA, whereas LATS2 could not be detected in Panc1 cells with the antibody used. Unexpectedly, like YAP, LATS1 itself was down-regulated after the inhibition of Rac1 (Figure 4A), as well as in HPAF/CD18 cells treated with EHT-1864 (Figure S4). Significantly, the depletion of one LATS isoform or the other did not prevent the degradation of YAP induced by EHT-1864, nor did the simultaneous depletion of both isoforms (Figure 4A). While they suggested a lack of involvement of the LATS1/2 kinases in the regulation of YAP by Rac1, a caveat with these experiments was our inability to detect the LATS2 protein to verify its complete depletion. To overcome this pitfall, in a second series of experiments, we used Hela cells engineered to lack all of their LATS1 and LATS2 gene copies. Both of these kinases were detected in the parental HeLa cells, but both were missing from the CRISPR/Cas9-modified HeLa cells (Figure 4B). Next, the LATS1/2-proficient and LATS1/2-deficient cells were exposed to either EHT-1864 (Figure 4C) or else the DMSO vehicle (Figure 4D), after which YAP levels were monitored. In control cells exposed to DMSO, YAP levels remained invariable in both the modified and unmodified HeLa cells (Figure 4D). However, in cells exposed to EHT-1864, YAP levels declined rapidly and this reduction was observed in both the LATS1/2-proficient and LATS1/2-deficient cells (Figure 4C). Therefore, in Hela cells also, neither of the LATS1/2 kinases was required for YAP degradation after the inhibition of Rac1.

### 3.7. The Activity of CK1 Is Needed for YAP Degradation After Rac1 Inhibition

Under conditions of active Hippo signaling, the LATS1/2 kinases phosphorylate YAP at serine S381 and this event creates a priming site needed for the S384- and S387-phosphorylation of YAP by CK1, the final step leading to YAP recognition by SCF^βTrCP^ followed by its degradation [43]. Under conditions of Rac1 inhibition, the SCF^βTrCP^ ligase is implicated in the degradation of YAP (Figure 3C–G), but the LATS1/2 kinases are not involved (Figure 4A–D). This finding raises the question as to whether the S384- and S387-phosphorylation of YAP by CK1 is still also needed for the SCF^βTrCP^-mediated degradation of YAP seen after Rac1 inhibition. To investigate the role of CK1 in the regulation of YAP by Rac1, Panc1 cells expressing Flag-YAP proteins were exposed to IC-261, an inhibitor of CK1δ and CK1ε [75]. In Figure 4E, Panc1 cells expressing wild-type Flag-YAP were pre-treated with IC-261 prior to the addition of EHT-1864. In cells pre-treated with the DMSO vehicle (No IC-261), the Flag-YAP protein declined rapidly once cells were exposed to EHT-1864. In contrast, in those pre-treated with IC-261, there was no decline in Flag-YAP protein observed after the addition of EHT-1864. Identical results were observed with Panc1 cells modified to express the 5SA mutant of Flag-YAP (Figure 4F). These results show that the activity of CK1 is still required for the degradation of YAP after Rac1 inhibition, even when the LATS1/2 phosphorylation sites have all been mutagenized, as in the 5SA mutant.

## 4. Discussion

The YAP/TAZ transcriptional coactivators are a central player in mechanotransduction. Their nuclear versus cytoplasmic location is influenced by cell shape/geometry, cell tension, cell stretching, extracellular matrix (ECM) stiffness, and cell–cell contacts [100,101]. How these two factors respond to diverse mechanical clues is complex and appears to involve both Hippo-dependent and Hippo-independent pathways. YAP/TAZ nuclear localization is directly impacted by the state of the actin cytoskeleton and its regulator [100,101,102]. The Ras and Rac1 are GTPases that have in common that they alter the actin cytoskeleton to promote cell migration, more directly so in the case of Rac1 [14,27,103]. During cell migration, GTP-bound Rac1 associates with the WAVE regulatory complex to drive Arp2/3 complex-mediated actin polymerization and lamellipodia formation [26,104]. Oncogenic Ras proteins promote cell migration and invasion, which are in parts mediated by Rac1 acting downstream of Ras [103]. In this article, we have compared Rac1 and K-Ras in their ability to regulate YAP levels in PC cells, using inhibitors of both (Figure 1), as well as activated mutants of both proteins (Figure 2). We used MRTX1133 to inhibit the oncogenic K-Ras protein in PDAC cells and saw no significant changes in YAP levels (Figure 1A and Figure S1). These results were in stark contrast with those obtained with the Rac1 inhibitors, which all led to declines in YAP levels (Figure 1B–H). These declines were observed in a wide range of cell lines (HPAF/CD18, AsPC1, L3.6pl, Panc1, HPNE, and HeLa) and with three different Rac1 inhibitors: EHT-1864, NSC23766, and Ehop-016. NSC23766 and Ehop-016 are structurally related and work by blocking the interaction of Rac1 with its GEFs [70,71,72,105]. EHT-1864 causes the loss of bound nucleotide, thereby preventing the binding of Rac1 to its GEF [68,69]. YAP levels were also reduced by Rac1^T17N^, a biological inhibitor of Rac1 [78]. This mutant acts by squelching GEFs away from Rac1 and related Rho GTPases by sequestering these GEFs onto Rac1^T17N^ complexes that fail to signal. While these pharmacological and biological inhibitors are imperfect tools with their separate off-target effects [106,107,108], they all have in common that they block the accumulation of active Rac1 [70,71,72,78,105]. Most significantly, YAP levels were also reduced in PC cells by the siRNA-mediated silencing of Rac1 (Figure 1G) or by the dominant negative mutant of Rac1, Rac1^T17N^ (Figure 1H). While we cannot exclude the involvement of other Rac1-related Rho GTPases, these observations reveal Rac1 as a key regulator of YAP levels in PC cells, more so than oncogenic K-Ras. A similar regulation of YAP by K-Ras and Rac1 was also observed in HPNE cells expressing activated mutants of the two proteins (Figure 2). Again, YAP levels were more significantly up-regulated by activated Rac1 (Rac1^G12V^) than by oncogenic K-Ras (K-Ras^G12D^). In the HPNE/E6/E7 cells, for example, total YAP was only modestly increased by K-Ras^G12D^ (~two-fold) but was increased by more than 11-fold by the activated Rac1 mutant (Rac1^G12V^). These results are in line with the results of Figure 1 revealing Rac1 as a more direct regulator of YAP levels than oncogenic K-Ras.

**Figure 5:**
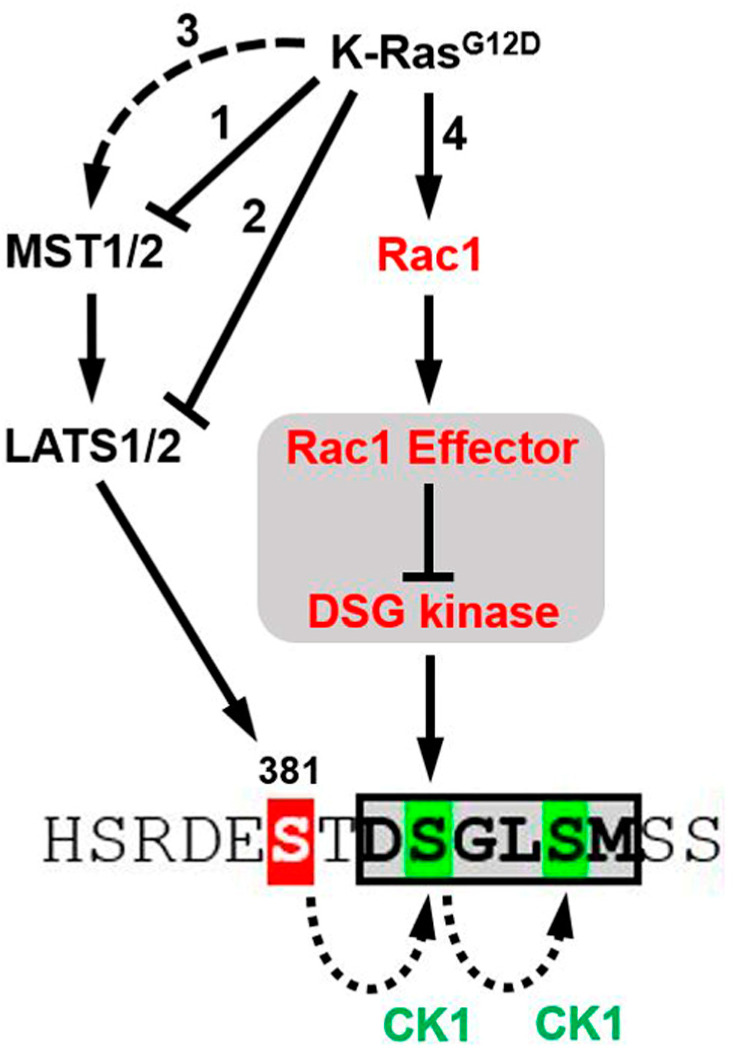
A model for the regulation of YAP stability by Rac1 and Ras. Previous studies have revealed complex interactions between the Hippo and Ras pathways, including both positive and negative regulations of MST1/2 and LATS1/2 by the Ras oncogenes. Downstream of Ras, YAP can be activated by the AKT phosphorylation and inhibition of MST2 (Arrow #1) [54,55] or the MAPK-mediated phosphorylation of Ajuba, resulting in LATS1/2 inhibition (Arrow #2) [37]. However, oncogenic Ras can also cause the RASSF1A-dependent activation of the MST2-LATS1 complex, leading to YAP phosphorylation and inhibition (Arrow #3) [54,55,58]. These interactions are expected to impact the S381-phosphorylation of YAP, both positively and negatively. This S381 phosphorylation creates a priming site that allows the S384/S387 phosphorylation of YAP (in green) by casein kinase 1 (CK1). This phosphorylation activates a βTrCP degron (boxed) that promotes the polyubiquitination of YAP by the SCF^βTrCP^ ligase and its degradation by the 26S proteasome. In this article, we describe a novel Rac1-regulated pathway that controls the stability of YAP (Arrow #4). This pathway operated outside of the Hippo pathway and did not require the LATS1/2 kinases or the phosphorylation of S381 to regulate YAP stability. Like the Hippo pathway, this other pathway regulated YAP stability in manners that were still dependent on both the integrity of the βTrCP degron and the kinase activity of CK1. We propose that this new pathway activates the βTrCP degron by the direct phosphorylation of S384 by a so-called “DSG” kinase. At the actin cytoskeleton (gray shaded area), the activity of this “DSG” kinase would normally be inhibited by Rac1 and its downstream effectors. Upon Rac1 inhibition, this kinase would rapidly phosphorylate S384, resulting in the CK1 phosphorylation of S387 and full activation of the degron. The existence of a so-called “DSG” kinase has been proposed before to explain the βTrCP-dependent proteolysis of Claspin [118,119].

There may be at least two reasons for this relative inability of oncogenic K-Ras to up-regulate YAP as much as the activated Rac1 mutant can. First and foremost, YAP responds to changes in the actin cytoskeleton [100,101,102] and Rac1 is a direct regulator of this cytoskeleton [26,104], whereas Ras relies on Rac1 and other Rho GTPases to impact its function [28,29,103]. Second, Ras has already been reported to have both positive and negative influences on YAP function (Figure 5) [37,54,55,56,57]. In particular, the Hippo/YAP pathway appears to be impacted in opposite ways by oncogenic Ras mutants and wild-type Ras signaling. YAP, for example, is activated by the EGF stimulation of wild-type Ras through both the AKT phosphorylation and inhibition of MST2 (Figure 5; #1) [54,55] and the phosphorylation of Ajuba resulting in LATS1/2 inhibition (Figure 5; #2) [37]. In stark contrast, the chronic expression of oncogenic Ras instead causes the RASSF1A-dependent activation of the MST2-LATS1 complex leading to YAP phosphorylation and inhibition (Figure 5; #3) [54,55,58]. RASSF1A possesses a Ras-binding domain (RBD) through which it serves as an effector of oncogenic K-Ras, one that promotes Ras-induced apoptosis [109,110]. Oncogenic K-Ras can also trigger oncogene-induced senescence (OIS), a form of senescence triggered in normal cells by oncogenic forms of Ras [95,96,97] and BRAF [98] (Figure 2C). Once engaged, the senescence program itself could also potentially inhibit YAP function, as suggested by mounting evidence [111,112,113,114,115,116,117]. However, unlike the oncogenic Ras proteins, the activated mutant of Rac1 did not trigger OIS (Figure 2C) and Rac1 is not a known activator of RASSF-type proteins [110]. Compared to oncogenic K-Ras, Rac1 is a more direct regulator of the actin cytoskeleton and Rac1 is less prone to inducing suppressive feedback mechanisms that can also block YAP (Figure 5; #3). Finally, in further support of the regulation of YAP by Rac1, a correlation between Rac1 and YAP levels was also observed across a panel of PC cell lines and HPNE cells (Figure 2A).

In Figure 3 and Figure 4, we dissected the mechanism involved in the down-regulation of YAP levels by EHT-1864. The results show that the process was dependent on the 26S proteasome (Figure 3A,B and Figure S3), the SCF^βTrCP^ E3 ubiquitin ligase (Figure 3F,G), the βTrCP degron of YAP (Figure 3C–E), and the kinase activity of CK1 (Figure 4E,F). Under conditions of elevated Hippo signaling, YAP degradation is normally initiated by the phosphorylation of S381 by LATS1/2 (XXX). This phosphorylation creates a priming site for CK1, which can then phosphorylate S384 and S387 of the βTrCP degron, activating the process (Figure 5). Unexpectedly, however, YAP degradation after Rac1 inhibition did not require the LATS1/2 kinases and could still be observed in the 5SA YAP mutants that carried the S381A mutation. One possibility for activation of the degron in Rac1-inhibited PC cells would be that S384 is being phosphorylated by a novel kinase (Figure 5). This phosphorylation would suffice to create a priming site for the subsequent phosphorylation of S387 by CK1, resulting in degron activation. In support of this model, the S384A mutation was sufficient to protect the Flag-YAP protein from degradation induced by the Rac1 inhibitor (Figure S5). In other substrates of SCF^βTrCP^, such as Claspin, the βTrCP degron is activated by the direct phosphorylation of its “DSG” motif [118,119]. In Figure 5, we propose that such a “DSG” kinase is responsible for the S384-phosphorylation of YAP when Rac1 is inhibited. Work is being undertaken to identify the kinase involved.

Studies implicating Rho GTPases as direct regulators of YAP function have been reported before [87,100,120,121]. In mesenchymal stem cells, YAP/TAZ function was regulated by the stiffness of the extracellular matrix and the shape of the cells [100]. In these cells, YAP/TAZ function was inhibited by drugs designed to block F-actin polymerization, actomyosin tension, or the activity of RhoA. Similar to what we report here for Rac1, this regulation was LATS1/2-independent and was accompanied by the degradation of TAZ. Could the same LATS1/2-independent mechanism be used in PC cells to regulate YAP by Rac1? Rac1 and RhoA share many of the same downstream effectors (e.g., PAK1-3 kinases) and Rac1 is hyperactivated in Ras-transformed PC cells, which could make it a more dominant regulator of their common effectors, including perhaps some involved in regulating YAP/TAZ. In a separate study, YAP/TAZ were instead controlled by yet another member of the Rho family, the Cdc42 GTPase [120]. In mouse models, the kidney-specific knockout of YAP and Cdc42 phenocopied each other, blocking nephron induction and morphogenesis. In the Cdc42 knockout, YAP and TAZ were excluded from the nucleus, and the transcription of YAP/TAZ-regulated genes was reduced. However, total YAP levels did not appear to be affected. Finally, a β-integrin/Rac1 pathway that regulates YAP has been described in osteoblasts. However, this regulation was mediated by the Hippo pathway and LATS1/2 kinases, as well as the PAK1-3 kinases acting downstream of Rac1 [87]. A different Rac1-regulated mechanism controls the levels of YAP in PC cells, given that it operates independently of the LATS1/2 (Figure 4A–D) or PAK1-3 (Figure 1B) kinases.

The Rac1 GTPase has been implicated in the regulation of the actin cytoskeleton [25,26], endocytosis/macropinocytosis [122,123], the cell cycle [124], and DNA damage response [33]. Similar to YAP [46], Rac1 is required for the transformation of primary cells by the Ras oncogenes [28,29]. Like YAP [47], it is also needed for the development of Ras-driven PC in the mouse [30,31]. Acting downstream of oncogenic K-Ras, the new Rac1/YAP axis described here could contribute to PC cell dissemination and metastasis by simultaneously promoting cell migration (Rac1) and making these cells more tolerant to inadequate cell–cell and cell–matrix interactions (YAP). The impact and the role of Rac1/YAP signaling could conceivably change during the development and progression of PC, depending on the presence of other genetic or epigenetic alterations. In Figure 2B–D, YAP was induced by activated Rac1, but only in the HPNE cells expressing E6/E7. Rac1/YAP signaling therefore appears to be restricted by the activities of p53 and/or p16/RB pathway. If so, then this pathway may be more critically important in the 50% and 70% of PC tumors that respectively lack functional p53 or p16 [2,125,126]. Likewise, RASSF1A expression is frequently repressed in PC specimens through promoter methylation [127]. In these tumors, YAP function would no longer be attenuated by the RASSF1A-mediated activation of MST1/2 (Figure 5; #3), thereby allowing oncogenic K-Ras to unimpededly activate YAP through its other effectors (Figure 5; #1 and #2), including Rac1 (Figure 5; #4). In these tumors, Rac1/YAP signaling could play a crucial role in boosting YAP levels and promoting invasion and metastasis. Future studies will be needed to better understand the role played by Rac1/YAP signaling in PC development and the response of these tumors to both conventional chemotherapies and inhibitors of oncogenic K-Ras proteins.

## 5. Conclusions

In pancreatic cells, we investigated the regulation of YAP by oncogenic K-Ras and its downstream effectors. Although YAP levels were only modestly influenced by the activity of oncogenic K-Ras, YAP stability was more directly controlled by the activity of Rac1, a known effector of Ras. This regulation of YAP by Rac1 was mediated by the SCF^βTrCP^ E3 ubiquitin ligase but in a manner that was independent of the LATS1/2 kinases and Hippo pathway. This novel Rac1/YAP axis that links a regulator of cell shape (i.e., Rac1) to a transducer of mechanical force (i.e., YAP) is likely to play an important role in the development of PC and other Ras-driven malignancies. The pathway could also potentially be involved in the development of therapeutic resistance to Ras inhibitors, which we know can be promoted by overexpressed YAP.

## Data Availability

The original contributions presented in the study are included in the article/Supplementary Material, further inquiries can be directed to the corresponding author.

## Supplementary Materials

The following supporting information can be downloaded at: https://www.mdpi.com/article/10.3390/cancers16213605/s1, Figure S1: YAP levels are unaffected by the inhibition of oncogenic K-Ras^G12D^ in PC cells; Figure S2: YAP levels are reduced by EHT-1864 in a dose-dependent manner; Figure S3: MG132 blocks the degradation of Flag-YAP proteins induced by the Rac1 inhibitor; Figure S4: LATS1 declines in PC cells after the inhibition of Rac1; Figure S5: The S384A mutation blocks YAP degradation elicited by the inhibition of Rac1; Scans of all individual blots used for generating the western blot figures.
