# Supplementary material for: Rac1 GTPase Regulates the βTrCP-Mediated Proteolysis of YAP Independently of the LATS1/2 Kinases
Source: Cancers (Basel). 2024 Oct 25;16(21):3605. doi: 10.3390/cancers16213605 (PMC11545309; doi:10.3390/cancers16213605)

SUPPLEMENT

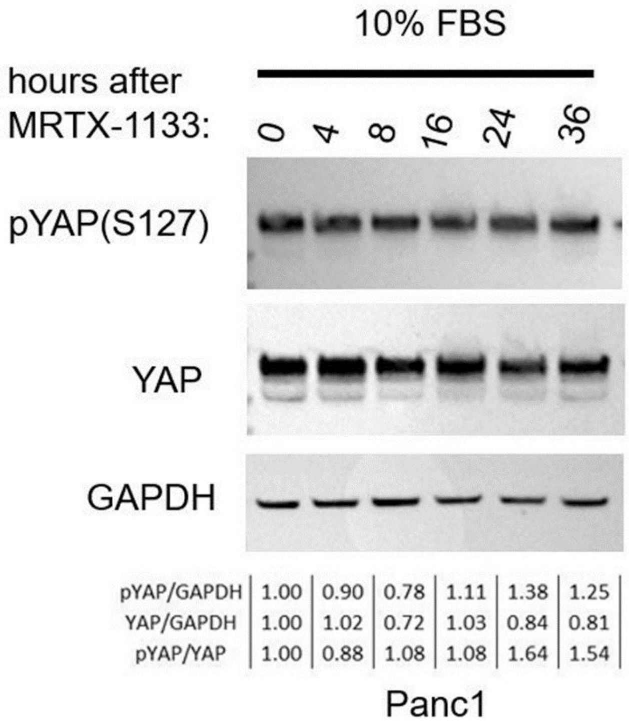

pYAP/GAPDH	1.00	0.90	0.78	1.11	1.38	1.25
YAP/GAPDH	1.00	1.02	0.72	1.03	0.84	0.81
pYAP/YAP	1.00	0.88	1.08	1.08	1.64	1.54

Figure S1: YAP levels are unaffected by the inhibition of oncogenic K-Ras^{G12D} in PC cells. Panc1 cells were exposed to MRTX1133 (50nM) for the indicated times. Levels of YAP and S127-phosphorylated YAP were measured by immunoblotting. GAPDH was used as an internal standard. Densitometry readings for the intensity ratio of pYAP/GAPDH, YAP/GAPDH, and pYAP/YAP are shown below, with the time 0 values arbitrarily set to 1.

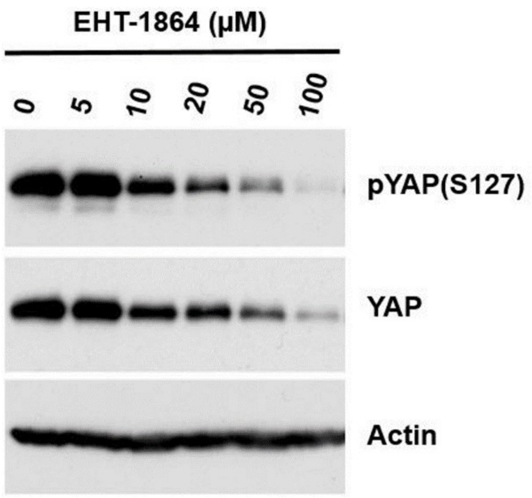

pYAP/GAPDH	1.00	1.02	0.59	0.36	0.20	0.04
YAP/GAPDH	1.00	0.94	0.63	0.56	0.35	0.14
pYAP/YAP	1.00	1.08	0.95	0.65	0.57	0.30

HPAF/CD18

Figure S2: YAP levels are reduced by EHT-1864 in a dose-dependent manner. HPAF/CD18 cells were exposed to different concentrations of EHT-1864 (0-100 µM). After 16 hours, levels of YAP and S127-phosphorylated YAP were measured by immunoblotting. Actin was used as an internal standard. Densitometry readings for the intensity ratio of pYAP/Actin, YAP/Actin, and pYAP/YAP are shown below, with the untreated control set to 1.

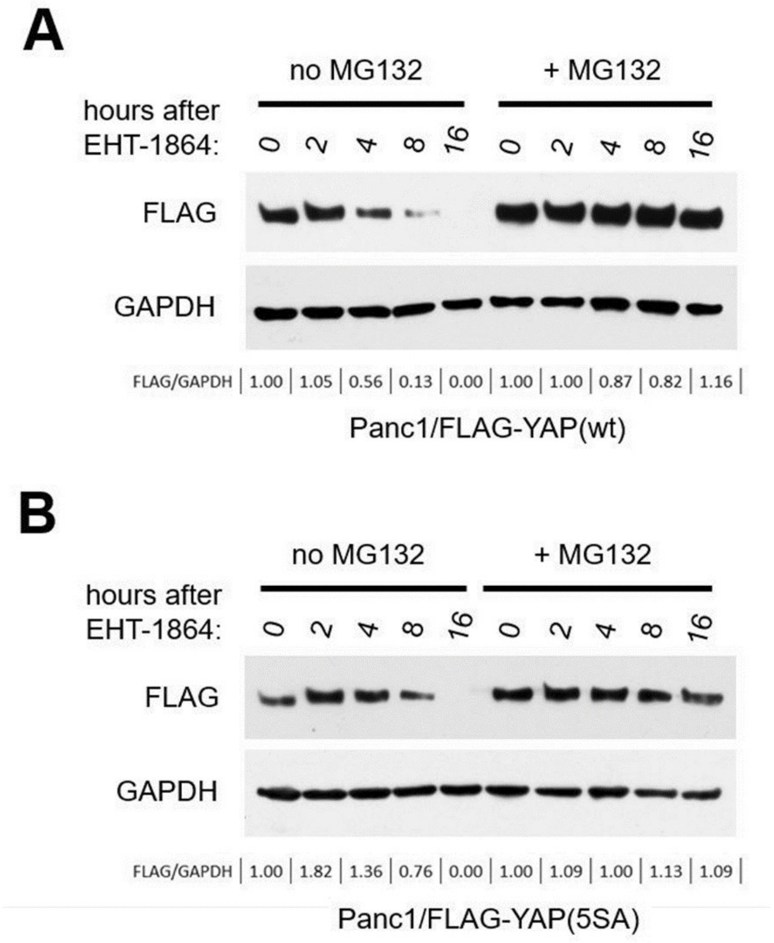

Figure S3: MG132 blocks the degradation of Flag-YAP proteins induced by the Rac1 inhibitor. Panc1 cells expressing the wild-type Flag-YAP protein **(A)** or its 5SA mutant **(B)** were first exposed to either MG132 (20 µg/ml) or DMSO vehicle (no MG132). After two hours of exposure, EHT-1864 (50 µM) was added, and cells were collected at the indicated time points. The Flag-tagged YAP proteins (wt, 5SA) were detected by immunoblotting using an anti-Flag antibody. GAPDH was used as a loading control. Densitometry readings for the intensity ratio of FLAG/GAPDH are shown below, with the time 0 values set to 1.

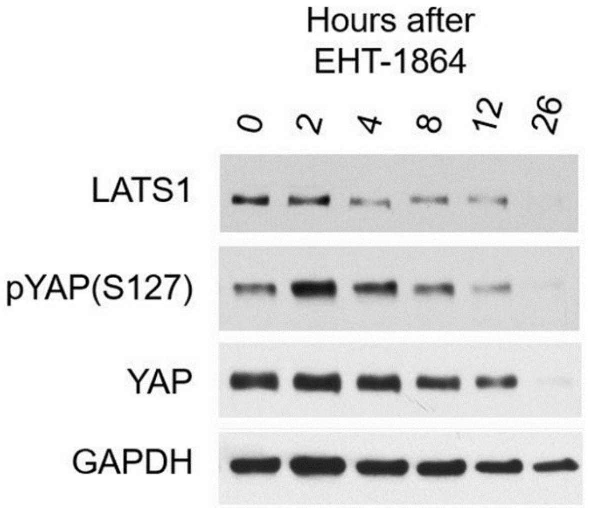

LATS1/GAPDH	1.00	0.79	0.40	0.41	0.40	0.00
pYAP/GAPDH	1.00	2.04	1.61	1.02	0.45	0.04
YAP/GAPDH	1.00	0.89	0.92	0.73	0.70	0.03
pYAP/YAP	1.00	2.28	1.75	1.41	0.64	1.45

HPAF/CD18

Figure S4: LATS1 declines in PC cells after the inhibition of Rac1. Levels of LATS1, YAP and S127-phosphorylated YAP were measured in HPAF/CD18 cells at the indicated time points after the addition of EHT-1864 (50 μM). GAPDH was used as an internal standard. Densitometry readings for the intensity ratio of LATS1/GAPDH, pYAP/GAPDH, YAP/GAPDH, and pYAP/YAP are shown below, with the time 0 values set to 1.

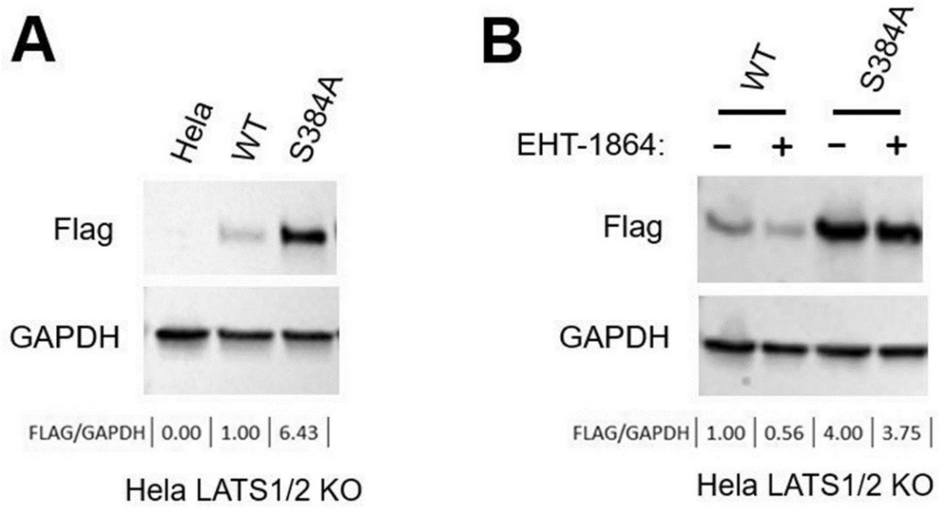

Figure S5: The S384A mutation blocks YAP degradation elicited by the inhibition of Rac1. (A) Levels of the Flag-YAP proteins in the infected LATS1/2-deficient HeLa cells. LATS1/2-deficient HeLa cells were transduced with retrovirally vectors expressing the wild-type Flag-YAP protein (WT) and its 384A mutant (S384A). After selection for retroviral integration (hygromycin 200 µg/ml), cells were analyzed by immunoblotting for the presence of the Flag-tagged YAP proteins using an anti-Flag antibody. The parental LATS1/2-deficient HeLa cells were also tested as an uninfected control. GAPDH was used as a loading control. **(B)** Impacts of Rac1 inhibition on the wild type and mutant Flag-YAP proteins. The two selected cell populations were exposed to either EHT-1864 (50 µM; +) or the DMSO vehicle (-). Sixteen hours later, levels of the Flag-YAP proteins were measured using the anti-Flag antibody. GAPDH was used as a loading control. For both panels, densitometry readings of the intensity ratio of FLAG/GAPDH are shown below, with the untreated cells expressing wild type Flag-YAP arbitrarily set to 1.

INDIVIDUAL FIGURES

Figure 1

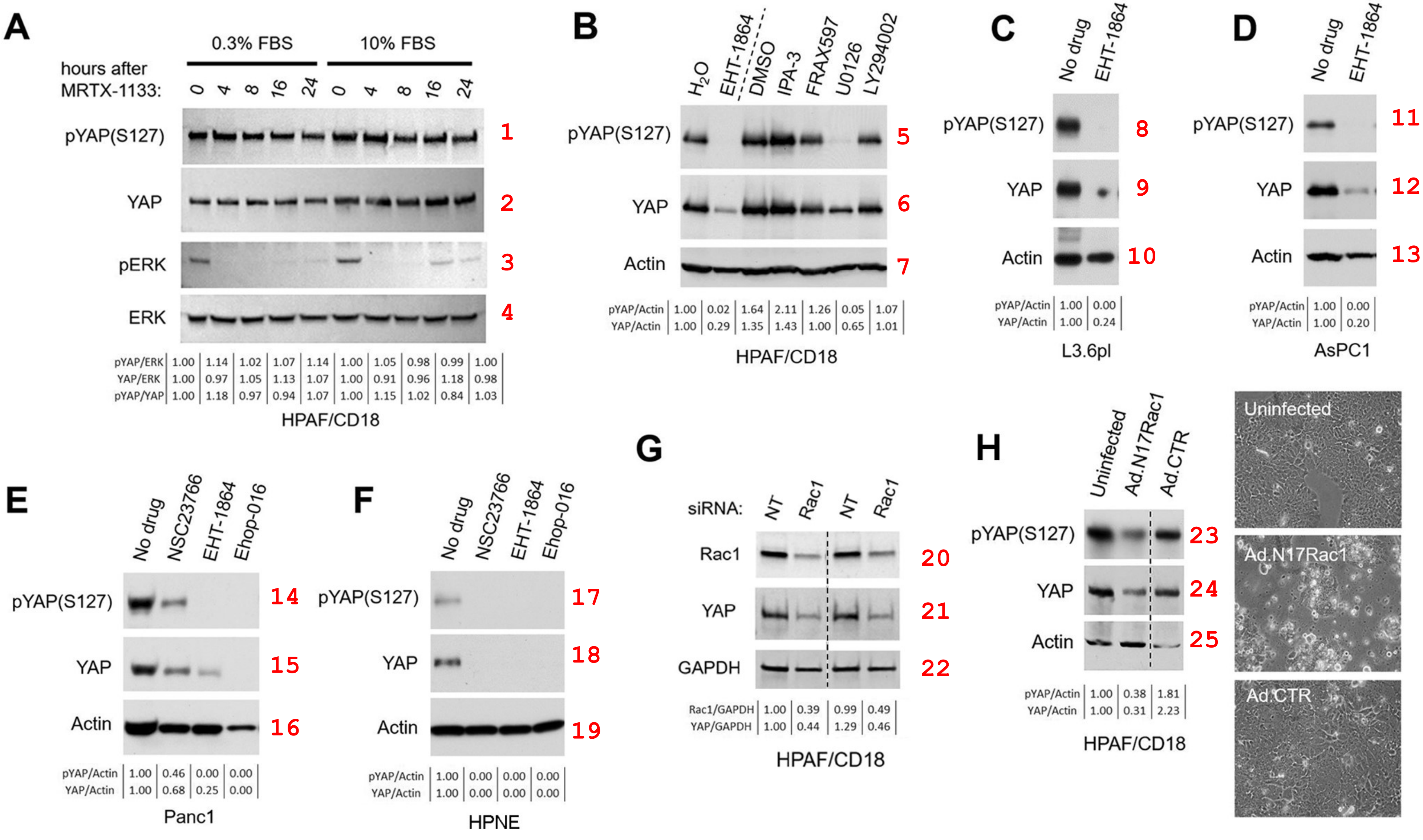
A
0.3% FBS
10% FBS
hours after MRTX-1133:
0 4 8 16 24 0 4 8 16 24
pYAP(S127)
YAP
pERK
ERK
1
2
3
4
pYAP/ERK 1.00 1.14 1.02 1.07 1.14 1.00 1.05 0.98 0.99 1.00
YAP/ERK 1.00 0.97 1.05 1.13 1.07 1.00 0.91 0.96 1.18 0.98
pYAP/YAP 1.00 1.18 0.97 0.94 1.07 1.00 1.15 1.02 0.84 1.03
HPAF/CD18
B
H2O
EHT-1864
DMSO
IPA-3
FRAX597
U0126
LY294002
pYAP(S127)
YAP
Actin
5
6
7
pYAP/Actin 1.00 0.02 1.64 2.11 1.26 0.05 1.07
YAP/Actin 1.00 0.29 1.35 1.43 1.00 0.65 1.01
HPAF/CD18
C
No drug
EHT-1864
pYAP(S127)
YAP
Actin
8
9
10
pYAP/Actin 1.00 0.00
YAP/Actin 1.00 0.24
L3.6pl
D
No drug
EHT-1864
pYAP(S127)
YAP
Actin
11
12
13
pYAP/Actin 1.00 0.00
YAP/Actin 1.00 0.20
AsPC1
E
No drug
NSC23766
EHT-1864
Ehop-016
pYAP(S127)
YAP
Actin
14
15
16
pYAP/Actin 1.00 0.46 0.00 0.00
YAP/Actin 1.00 0.68 0.25 0.00
Panc1
F
No drug
NSC23766
EHT-1864
Ehop-016
pYAP(S127)
YAP
Actin
17
18
19
pYAP/Actin 1.00 0.00 0.00 0.00
YAP/Actin 1.00 0.00 0.00 0.00
HPNE
G
siRNA:
NT
Rac1
NT
Rac1
Rac1
YAP
GAPDH
20
21
22
Rac1/GAPDH 1.00 0.39 0.99 0.49
YAP/GAPDH 1.00 0.44 1.29 0.46
HPAF/CD18
H
Uninfected
Ad.N17Rac1
Ad.CTR
pYAP(S127)
YAP
Actin
23
24
25
pYAP/Actin 1.00 0.38 1.81
YAP/Actin 1.00 0.31 2.23
HPAF/CD18
Uninfected
Ad.N17Rac1
Ad.CTR

Figure 2

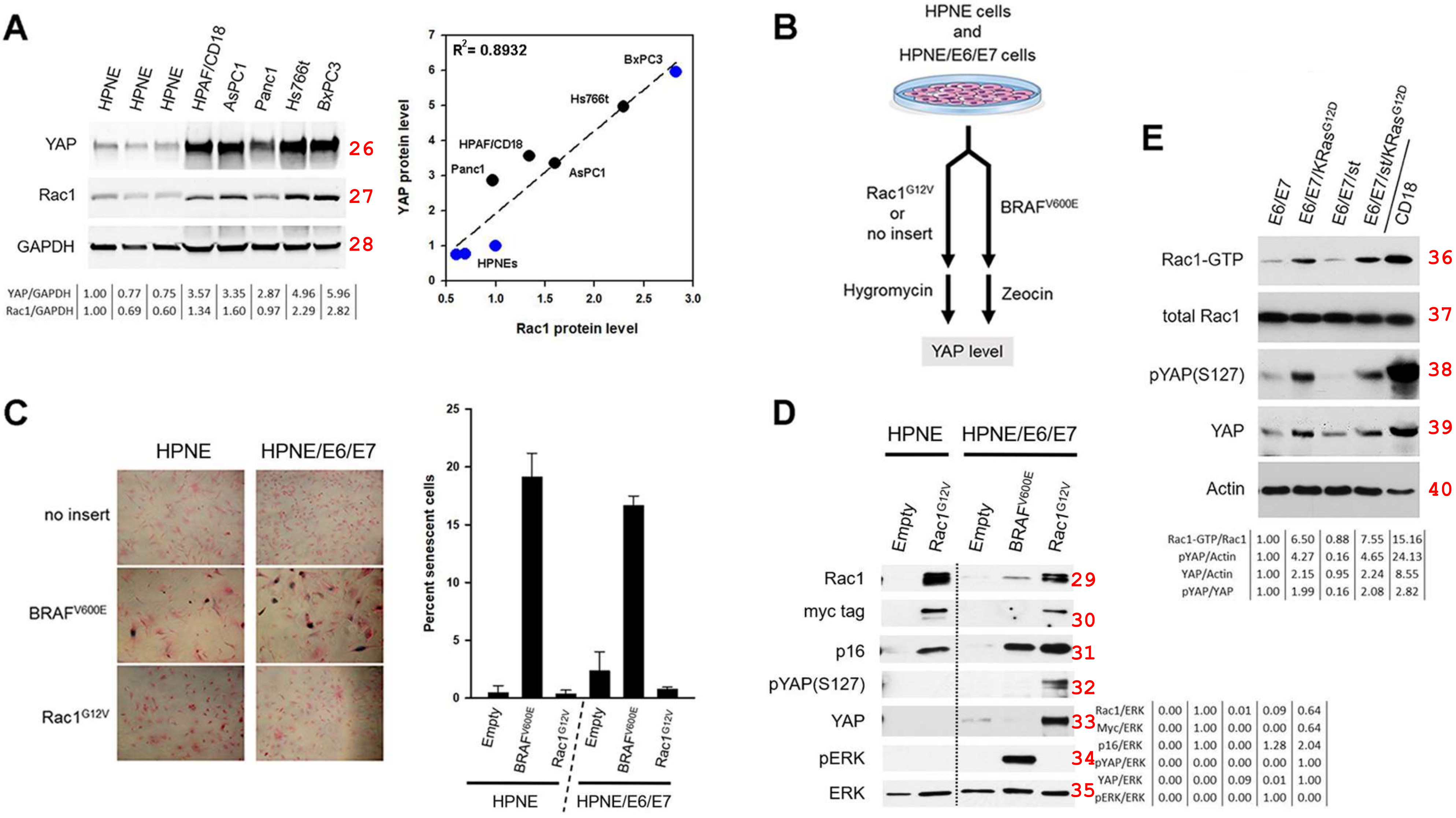
A
HPNE
HPNE
HPNE
HPAF/CD18
AsPC1
Panc1
Hs766t
BxPC3
YAP
Rac1
GAPDH
26
27
28
YAP/GAPDH 1.00 0.77 0.75 3.57 3.35 2.87 4.96 5.96
Rac1/GAPDH 1.00 0.69 0.60 1.34 1.60 0.97 2.29 2.82
R2= 0.8932
BxPC3
Hs766t
HPAF/CD18
Panc1
AsPC1
HPNEs
YAP protein level
Rac1 protein level
B
HPNE cells
and
HPNE/E6/E7 cells
Rac1G12V
or
no insert
BRAFV600E
Hygromycin
Zeocin
YAP level
C
HPNE
HPNE/E6/E7
no insert
BRAFV600E
Rac1G12V
Percent senescent cells
Empty
BRAFV600E
Rac1G12V
Empty
BRAFV600E
Rac1G12V
HPNE
HPNE/E6/E7
D
HPNE
HPNE/E6/E7
Empty
Rac1G12V
Empty
BRAFV600E
Rac1G12V
Rac1
myc tag
p16
pYAP(S127)
YAP
pERK
ERK
29
30
31
32
33
34
35
Rac1/ERK 0.00 1.00 0.01 0.09 0.64
Myc/ERK 0.00 1.00 0.00 0.00 0.64
p16/ERK 0.00 1.00 0.00 1.28 2.04
pYAP/ERK 0.00 0.00 0.00 0.00 1.00
YAP/ERK 0.00 0.00 0.09 0.01 1.00
pERK/ERK 0.00 0.00 0.00 1.00 0.00
E
E6/E7
E6/E7/KRasG12D
E6/E7/st
E6/E7/st/KRasG12D
CD18
Rac1-GTP
total Rac1
pYAP(S127)
YAP
Actin
36
37
38
39
40
Rac1-GTP/Rac1 1.00 6.50 0.88 7.55 15.16
pYAP/Actin 1.00 4.27 0.16 4.65 24.13
YAP/Actin 1.00 2.15 0.95 2.24 8.55
pYAP/YAP 1.00 1.99 0.16 2.08 2.82

Figure 3

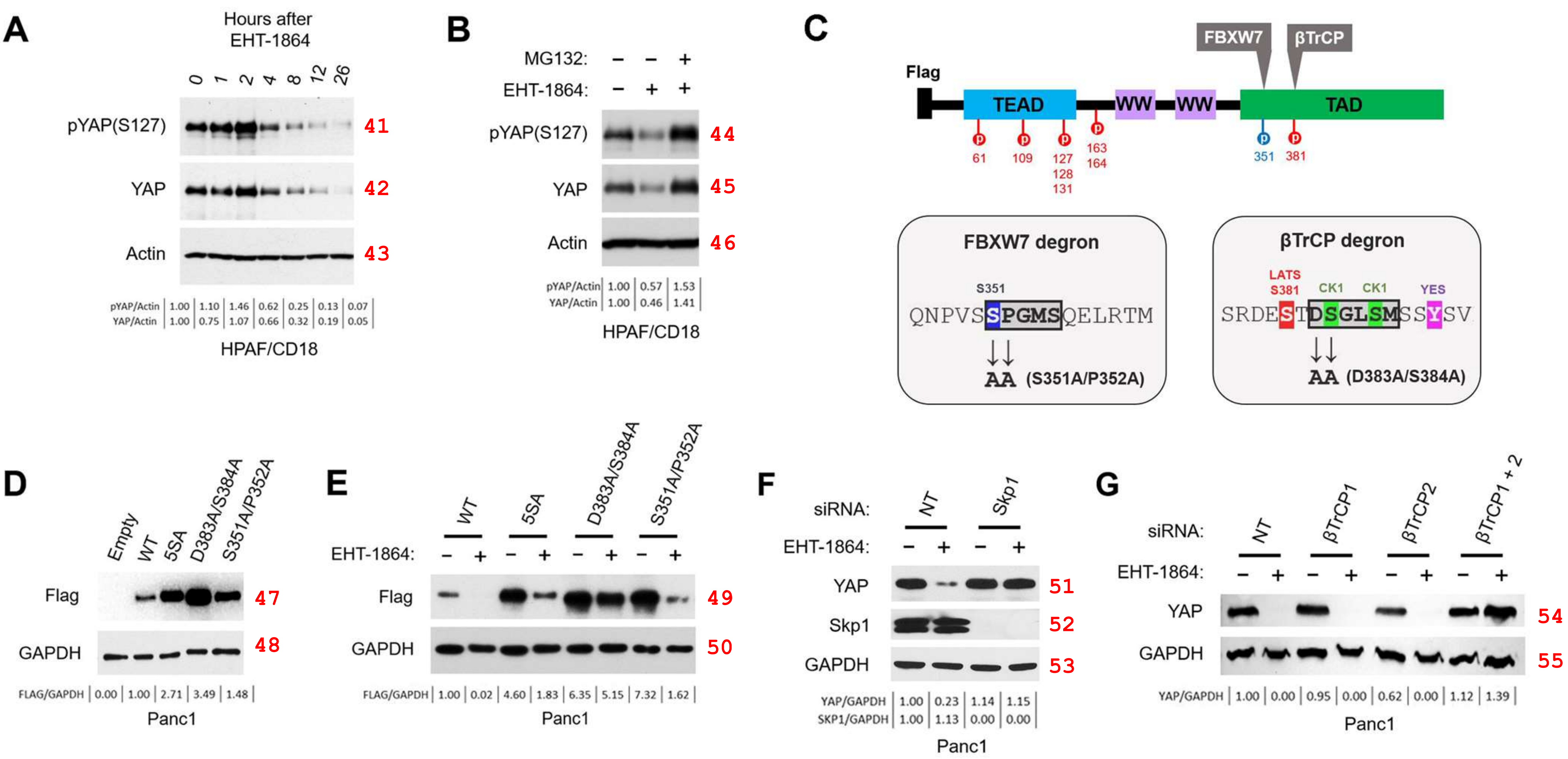
A
Hours after EHT-1864
0 1 2 4 8 12 26
pYAP(S127)
YAP
Actin
41
42
43
pYAP/Actin 1.00 1.10 1.46 0.62 0.25 0.13 0.07
YAP/Actin 1.00 0.75 1.07 0.66 0.32 0.19 0.05
HPAF/CD18
B
MG132: − − +
EHT-1864: − + +
pYAP(S127)
YAP
Actin
44
45
46
pYAP/Actin 1.00 0.57 1.53
YAP/Actin 1.00 0.46 1.41
HPAF/CD18
C
FBXW7
βTrCP
Flag
TEAD
WW
WW
TAD
61 109 127 128 131 163 164
351 381
FBXW7 degron
S351
QNPVSSPGMSQELRTM
AA (S351A/P352A)
βTrCP degron
LATS S381
CK1
CK1
YES
SRDESTDSGLSMSSYSV
AA (D383A/S384A)
D
Empty
WT
5SA
D383A/S384A
S351A/P352A
Flag
GAPDH
47
48
FLAG/GAPDH 0.00 1.00 2.71 3.49 1.48
Panc1
E
WT
5SA
D383A/S384A
S351A/P352A
EHT-1864: − + − + − + − +
Flag
GAPDH
49
50
FLAG/GAPDH 1.00 0.02 4.60 1.83 6.35 5.15 7.32 1.62
Panc1
F
siRNA: NT Skp1
EHT-1864: − + − +
YAP
Skp1
GAPDH
51
52
53
YAP/GAPDH 1.00 0.23 1.14 1.15
SKP1/GAPDH 1.00 1.13 0.00 0.00
Panc1
G
siRNA: NT βTrCP1 βTrCP2 βTrCP1 + 2
EHT-1864: − + − + − + − +
YAP
GAPDH
54
55
YAP/GAPDH 1.00 0.00 0.95 0.00 0.62 0.00 1.12 1.39
Panc1

Figure 4

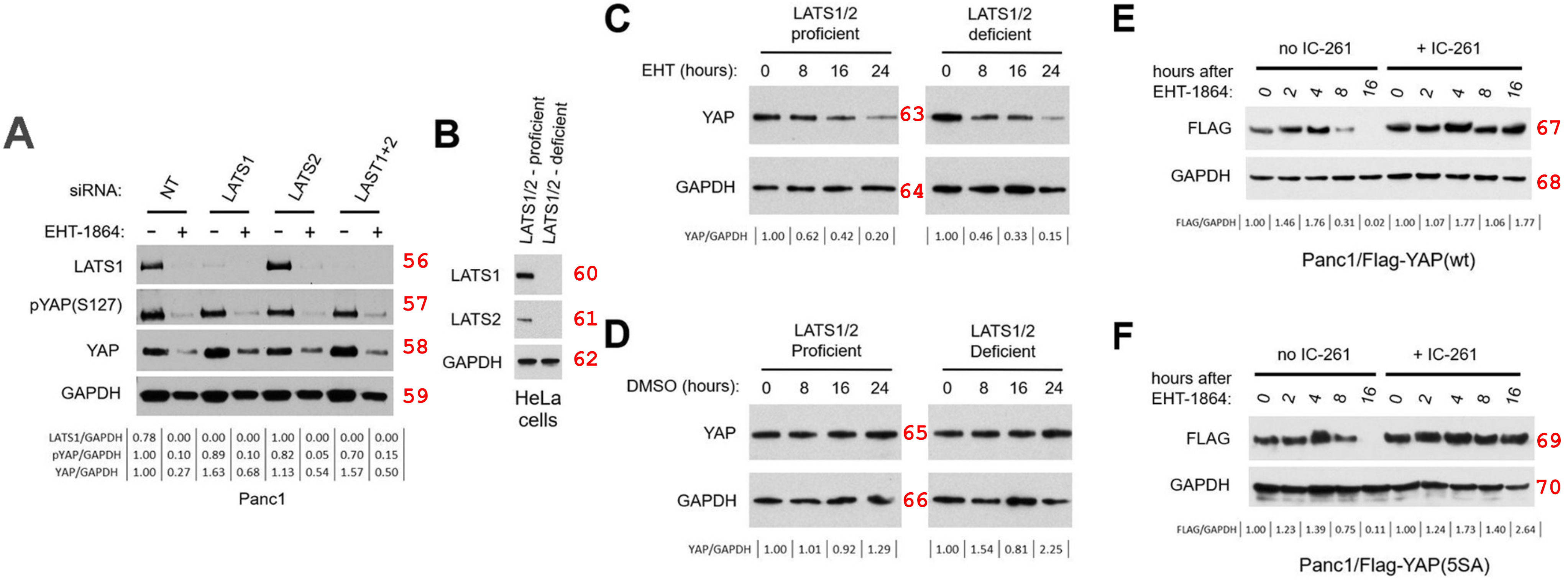
A
siRNA:
NT
LATS1
LATS2
LAST1+2
EHT-1864:
LATS1
pYAP(S127)
YAP
GAPDH
56
57
58
59
LATS1/GAPDH 0.78 0.00 0.00 0.00 1.00 0.00 0.00 0.00
pYAP/GAPDH 1.00 0.10 0.89 0.10 0.82 0.05 0.70 0.15
YAP/GAPDH 1.00 0.27 1.63 0.68 1.13 0.54 1.57 0.50
Panc1
B
LATS1/2 - proficient
LATS1/2 - deficient
LATS1
LATS2
GAPDH
60
61
62
HeLa cells
C
LATS1/2 proficient
LATS1/2 deficient
EHT (hours): 0 8 16 24 0 8 16 24
YAP
GAPDH
63
64
YAP/GAPDH 1.00 0.62 0.42 0.20 1.00 0.46 0.33 0.15
D
LATS1/2 Proficient
LATS1/2 Deficient
DMSO (hours): 0 8 16 24 0 8 16 24
YAP
GAPDH
65
66
YAP/GAPDH 1.00 1.01 0.92 1.29 1.00 1.54 0.81 2.25
E
no IC-261
+ IC-261
hours after EHT-1864: 0 2 4 8 16 0 2 4 8 16
FLAG
GAPDH
67
68
FLAG/GAPDH 1.00 1.46 1.76 0.31 0.02 1.00 1.07 1.77 1.06 1.77
Panc1/Flag-YAP(wt)
F
no IC-261
+ IC-261
hours after EHT-1864: 0 2 4 8 16 0 2 4 8 16
FLAG
GAPDH
69
70
FLAG/GAPDH 1.00 1.23 1.39 0.75 0.11 1.00 1.24 1.73 1.40 2.64
Panc1/Flag-YAP(5SA)

Figure 5

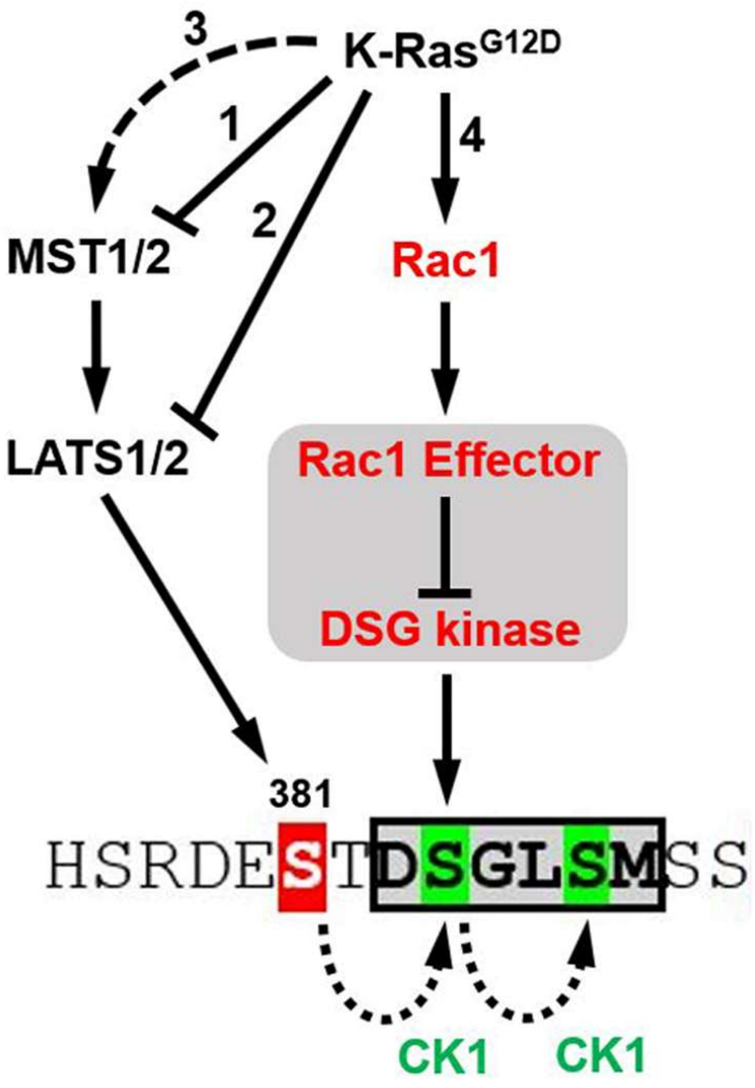

K-Ras^{G12D}
3
1
2
4
MST1/2
LATS1/2
Rac1
Rac1 Effector
DSG kinase
381
HSRDESTDSGLSMSS
CK1
CK1

Figure S1

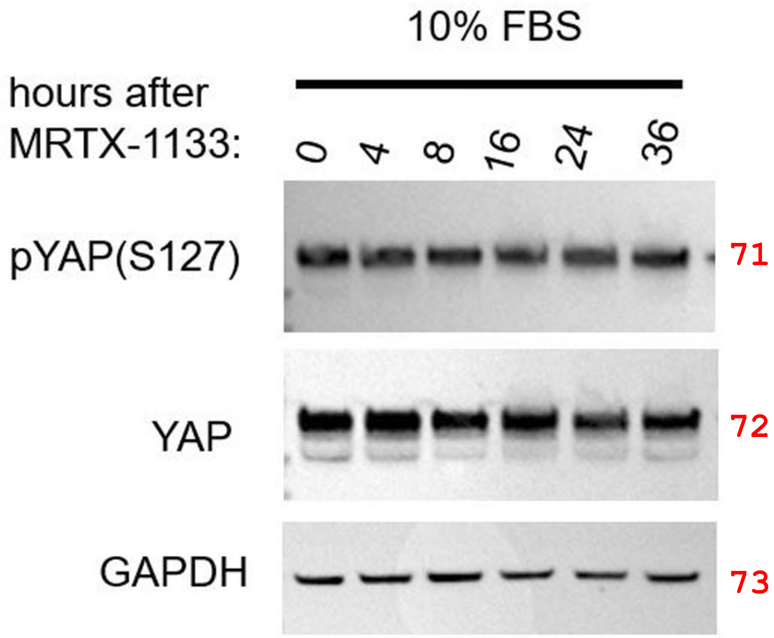

pYAP/GAPDH	1.00	0.90	0.78	1.11	1.38	1.25
YAP/GAPDH	1.00	1.02	0.72	1.03	0.84	0.81
pYAP/YAP	1.00	0.88	1.08	1.08	1.64	1.54

Panc1

Figure S2

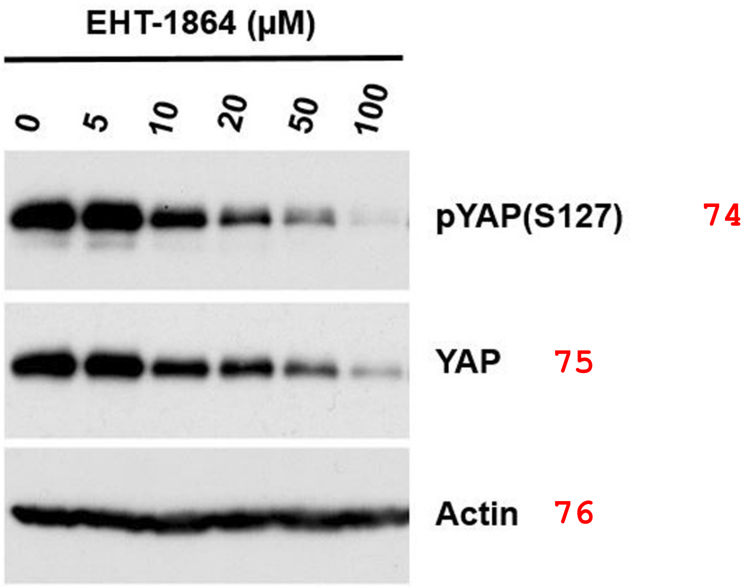

pYAP/GAPDH	1.00	1.02	0.59	0.36	0.20	0.04
YAP/GAPDH	1.00	0.94	0.63	0.56	0.35	0.14
pYAP/YAP	1.00	1.08	0.95	0.65	0.57	0.30

HPAF/CD18

Figure S3

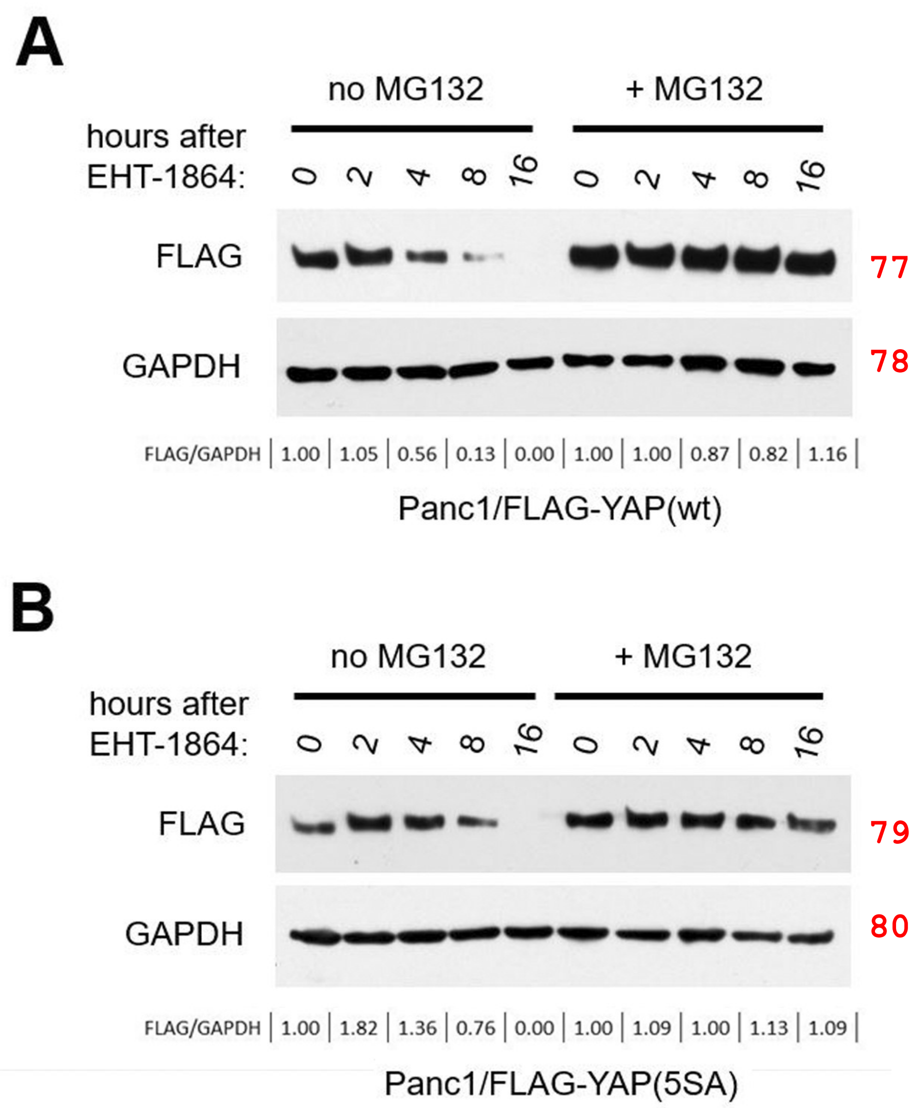
A
no MG132
+ MG132
hours after
EHT-1864:
0
2
4
8
16
0
2
4
8
16
FLAG
77
GAPDH
78
FLAG/GAPDH | 1.00 | 1.05 | 0.56 | 0.13 | 0.00 | 1.00 | 1.00 | 0.87 | 0.82 | 1.16 |
Panc1/FLAG-YAP(wt)
B
no MG132
+ MG132
hours after
EHT-1864:
0
2
4
8
16
0
2
4
8
16
FLAG
79
GAPDH
80
FLAG/GAPDH | 1.00 | 1.82 | 1.36 | 0.76 | 0.00 | 1.00 | 1.09 | 1.00 | 1.13 | 1.09 |
Panc1/FLAG-YAP(5SA)

Figure S4

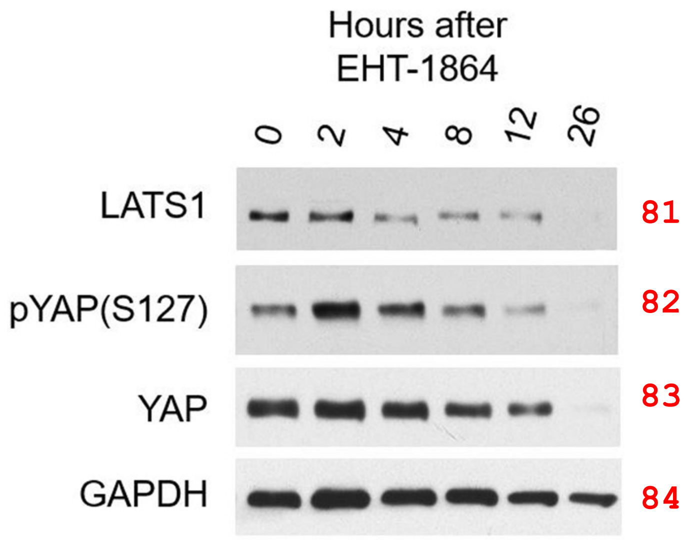

LATS1/GAPDH	1.00	0.79	0.40	0.41	0.40	0.00
pYAP/GAPDH	1.00	2.04	1.61	1.02	0.45	0.04
YAP/GAPDH	1.00	0.89	0.92	0.73	0.70	0.03
pYAP/YAP	1.00	2.28	1.75	1.41	0.64	1.45

HPAF/CD18

Figure S5

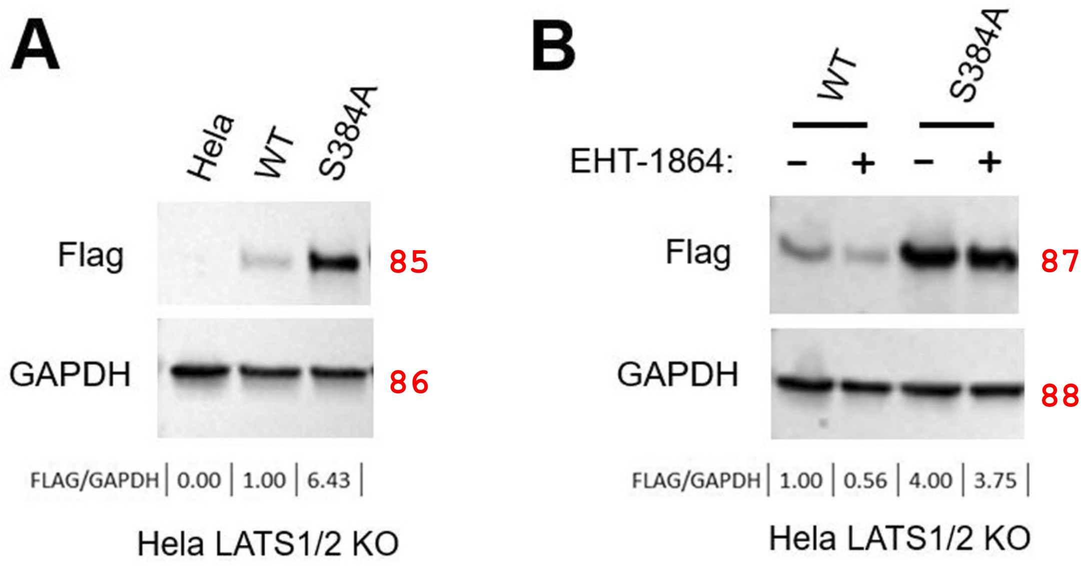
A
Hela
WT
S384A
Flag
85
GAPDH
86
FLAG/GAPDH | 0.00 | 1.00 | 6.43 |
Hela LATS1/2 KO
B
WT
S384A
EHT-1864: – + – +
Flag
87
GAPDH
88
FLAG/GAPDH | 1.00 | 0.56 | 4.00 | 3.75 |
Hela LATS1/2 KO

INDIVIDUAL BLOTS

Figure 1A: Blots 1-4

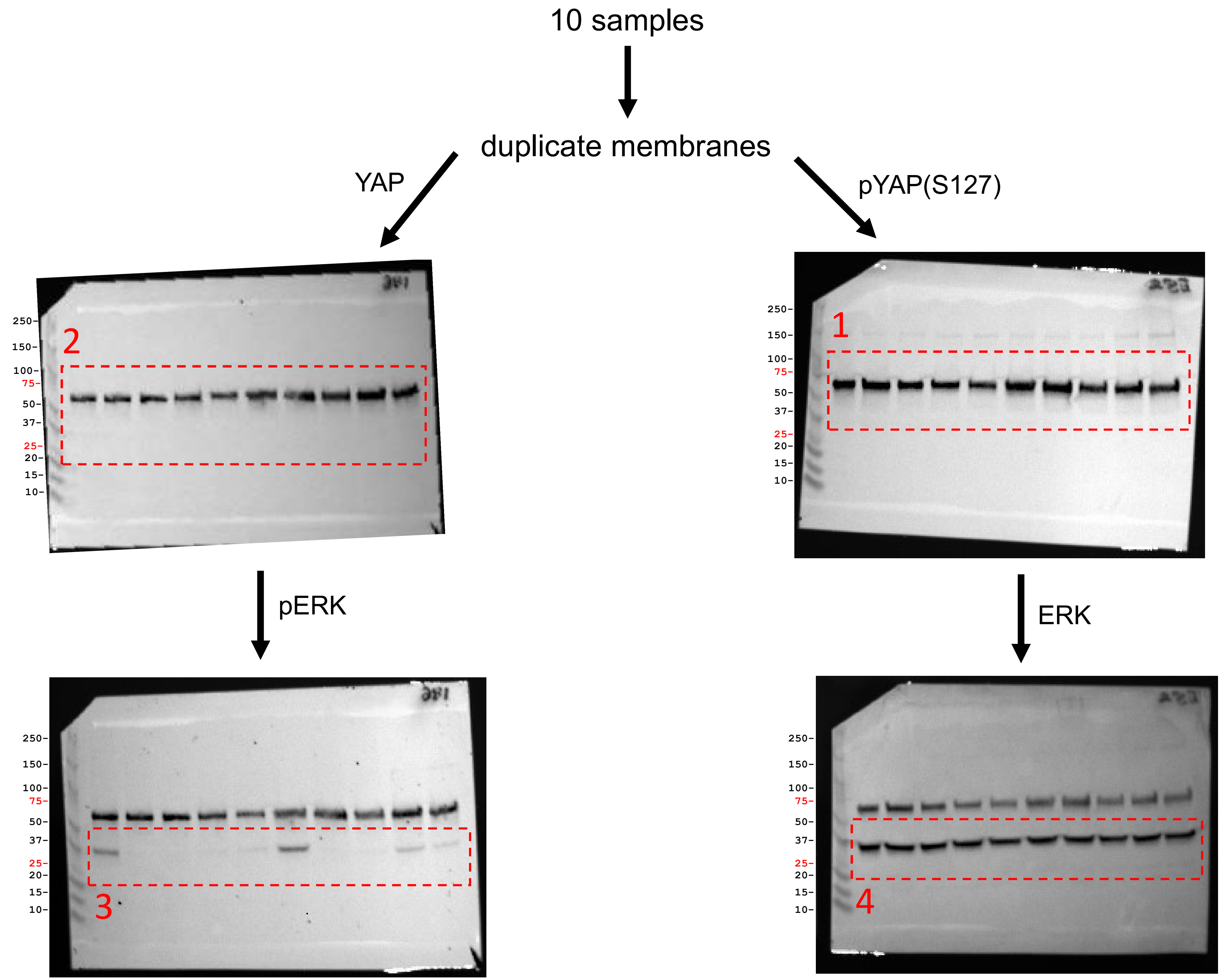

Figure 1B: Blots 5-7

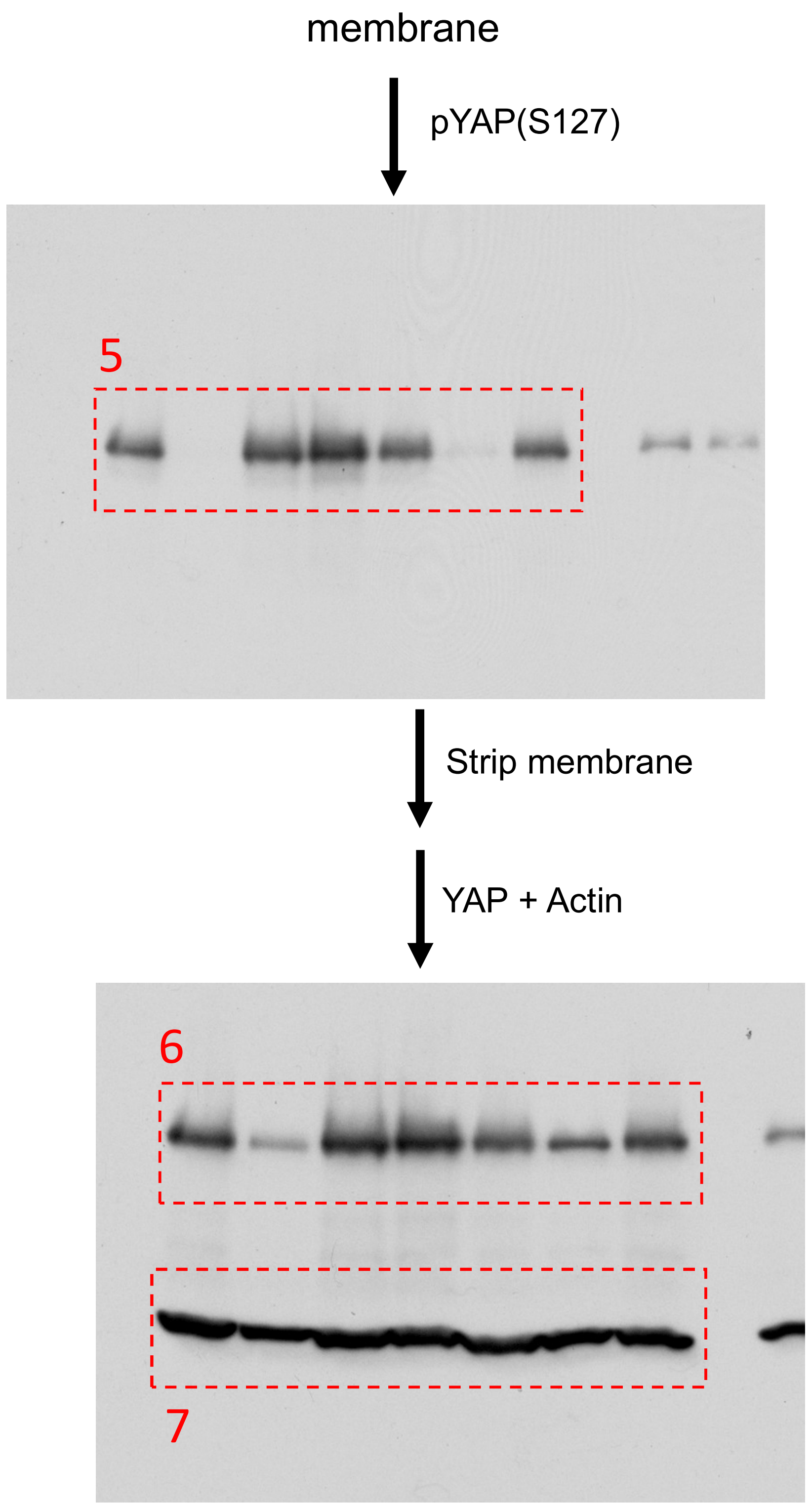

Figure 1C: Blots 8-10

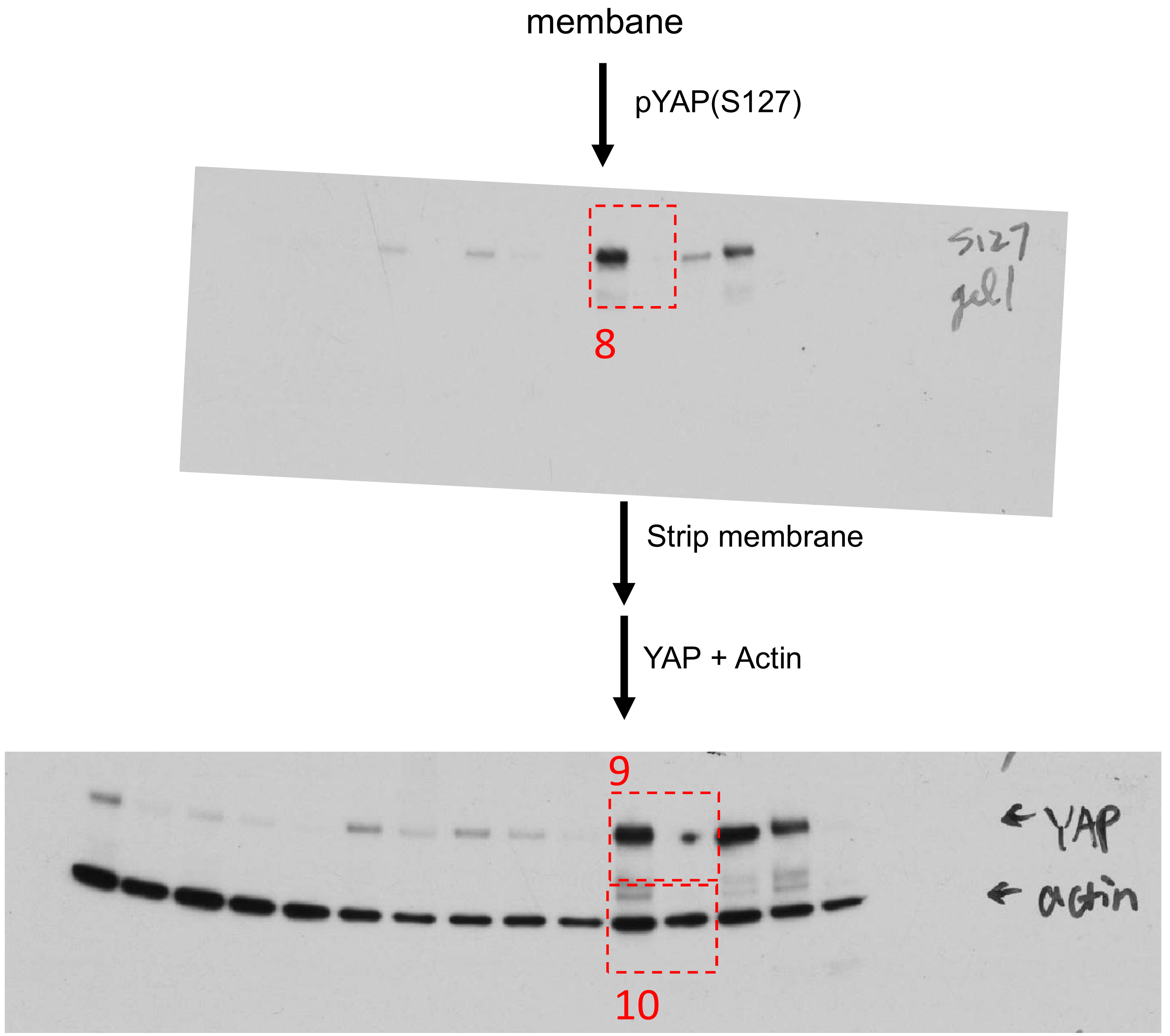

Figure 1D: Blots 11-13

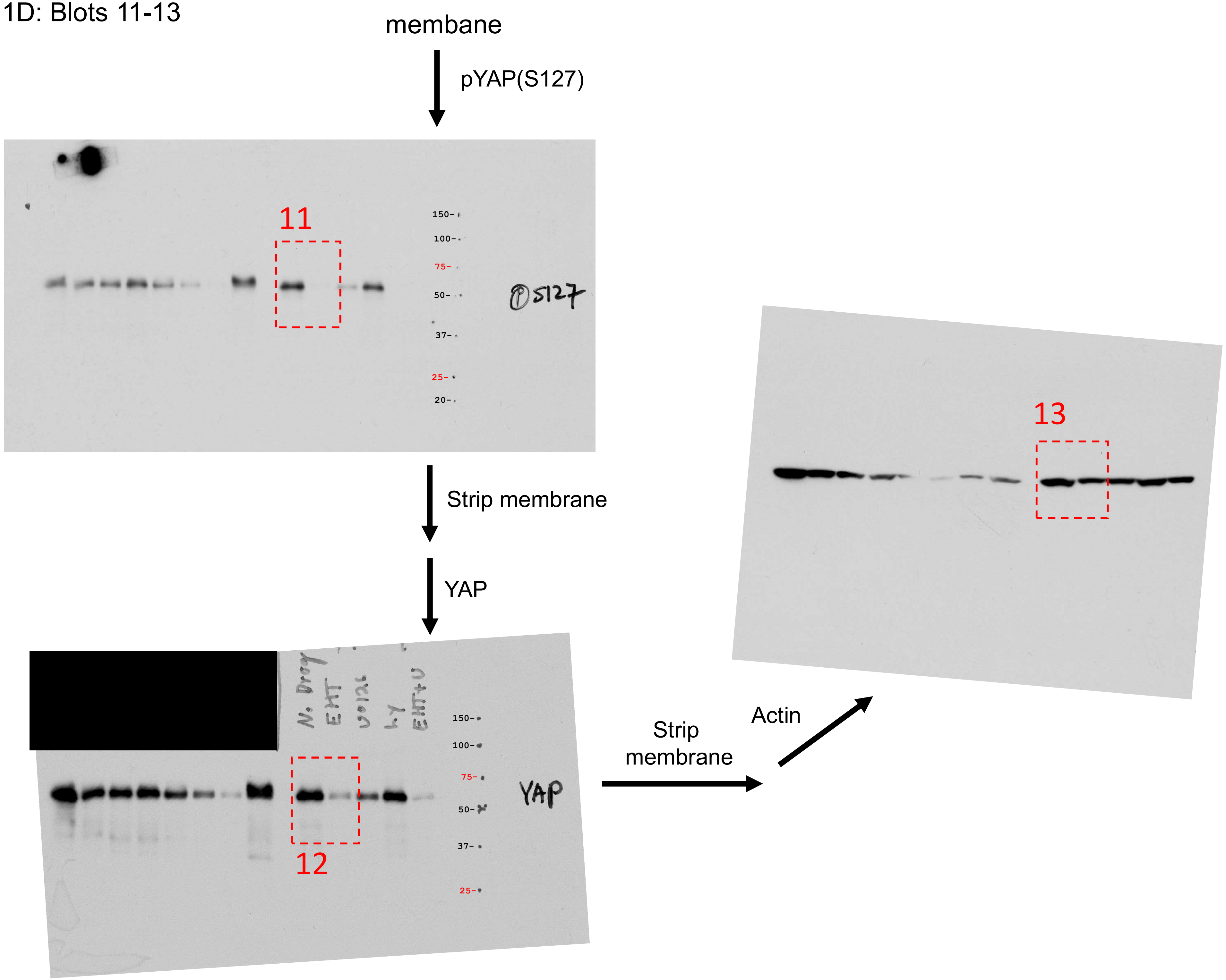

Figure 1E: Blots 14-16

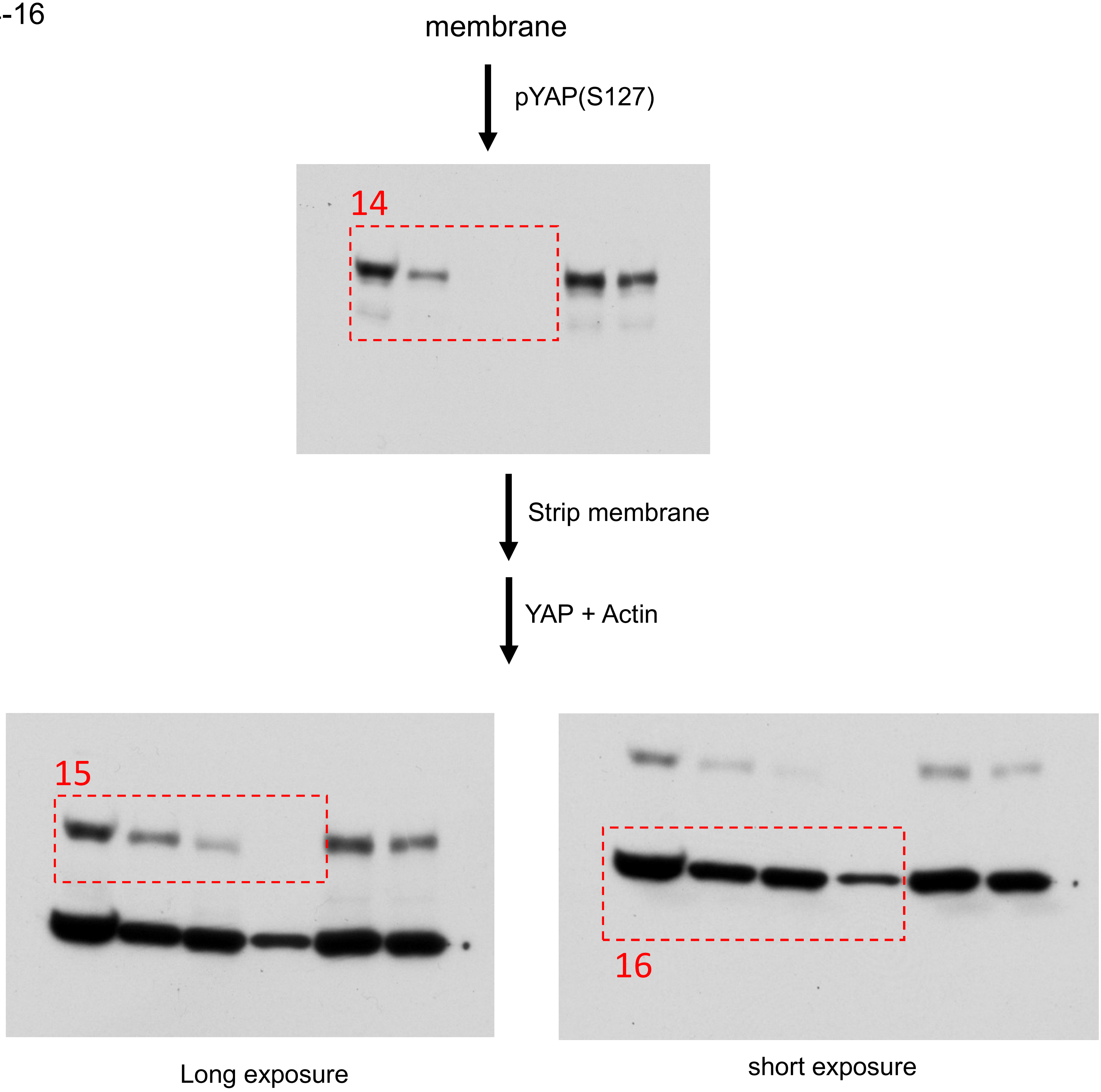

Figure 1F: Blots 17-19

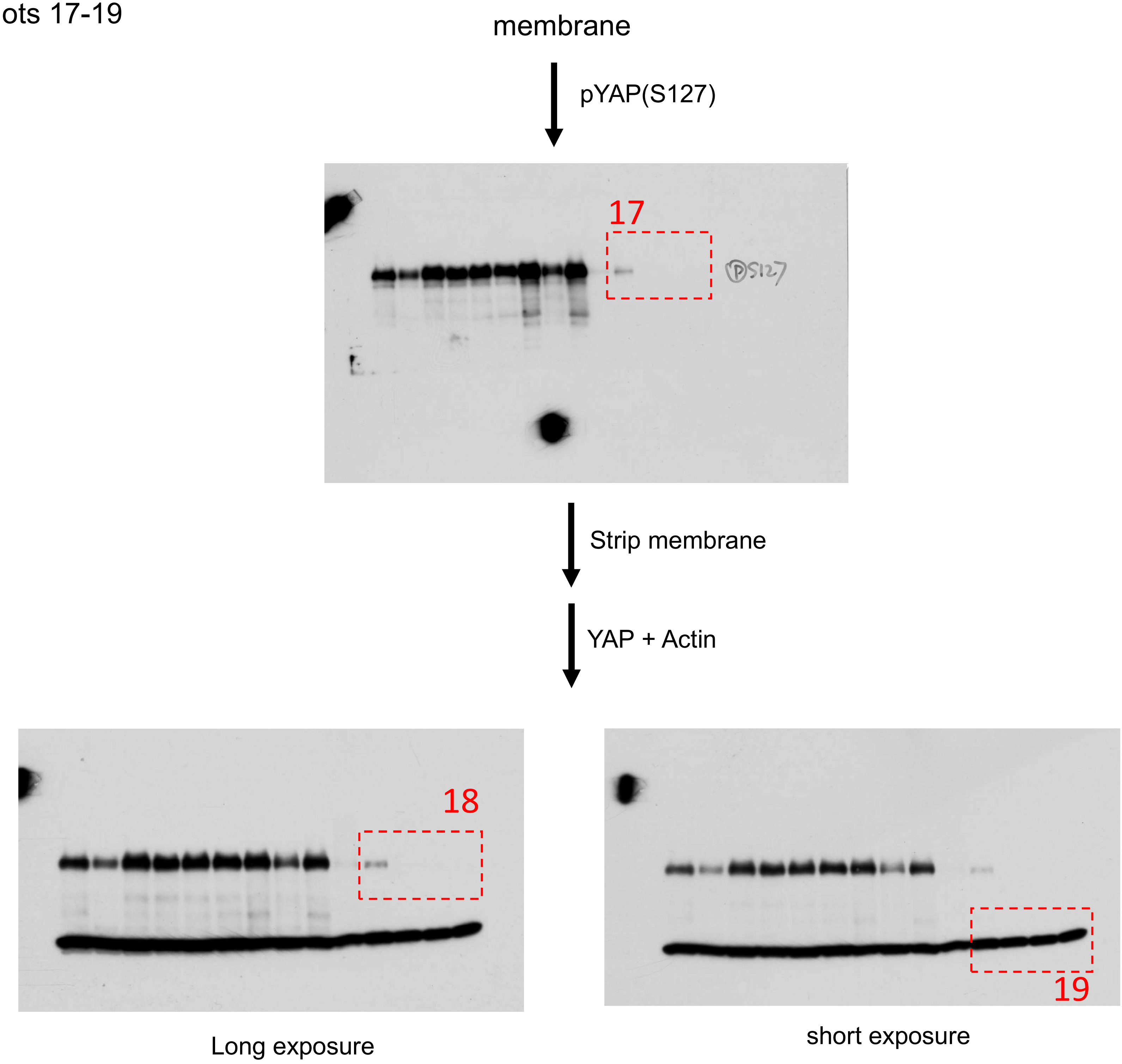

Figure 1G: Blots 20-22

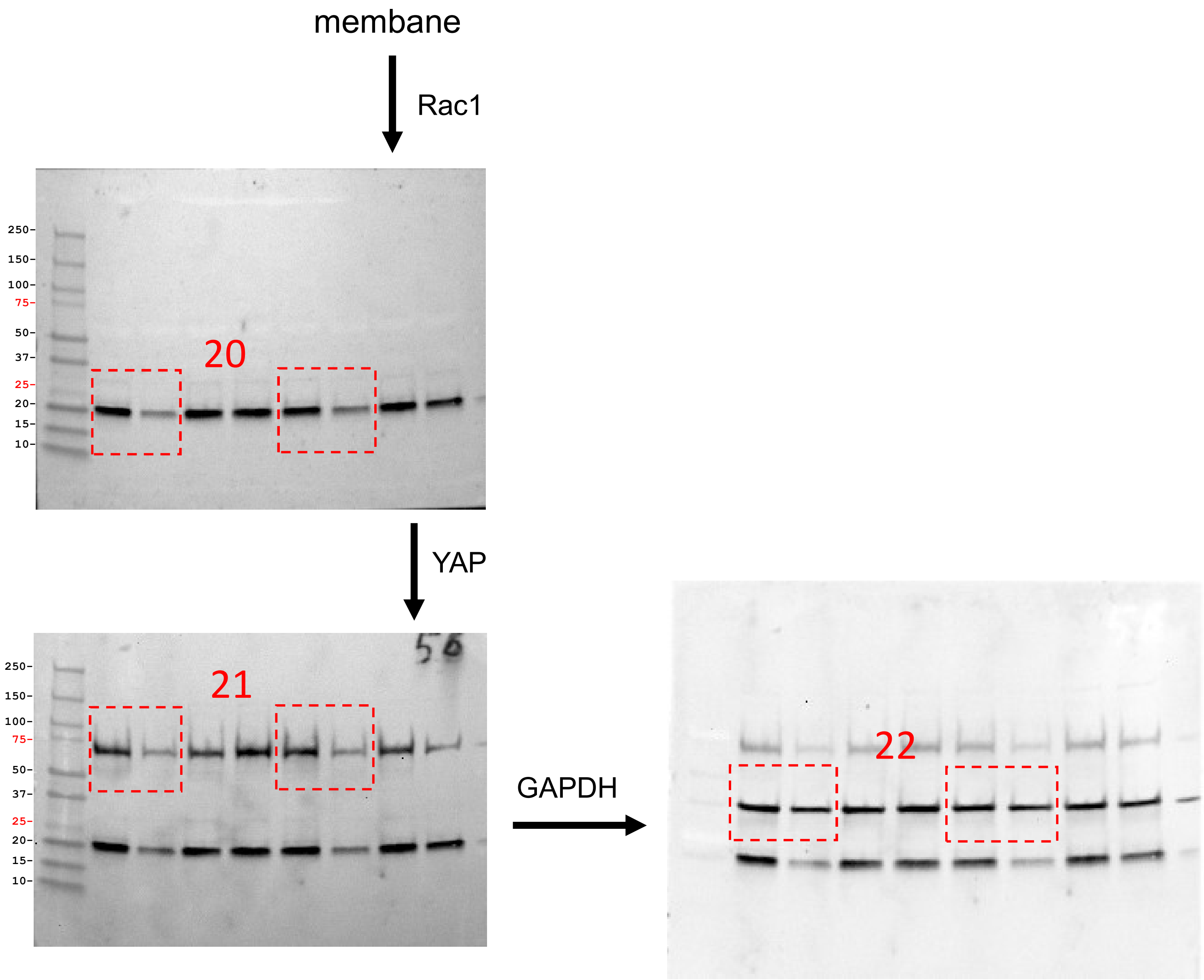

Figure 1H: Blots 23-25

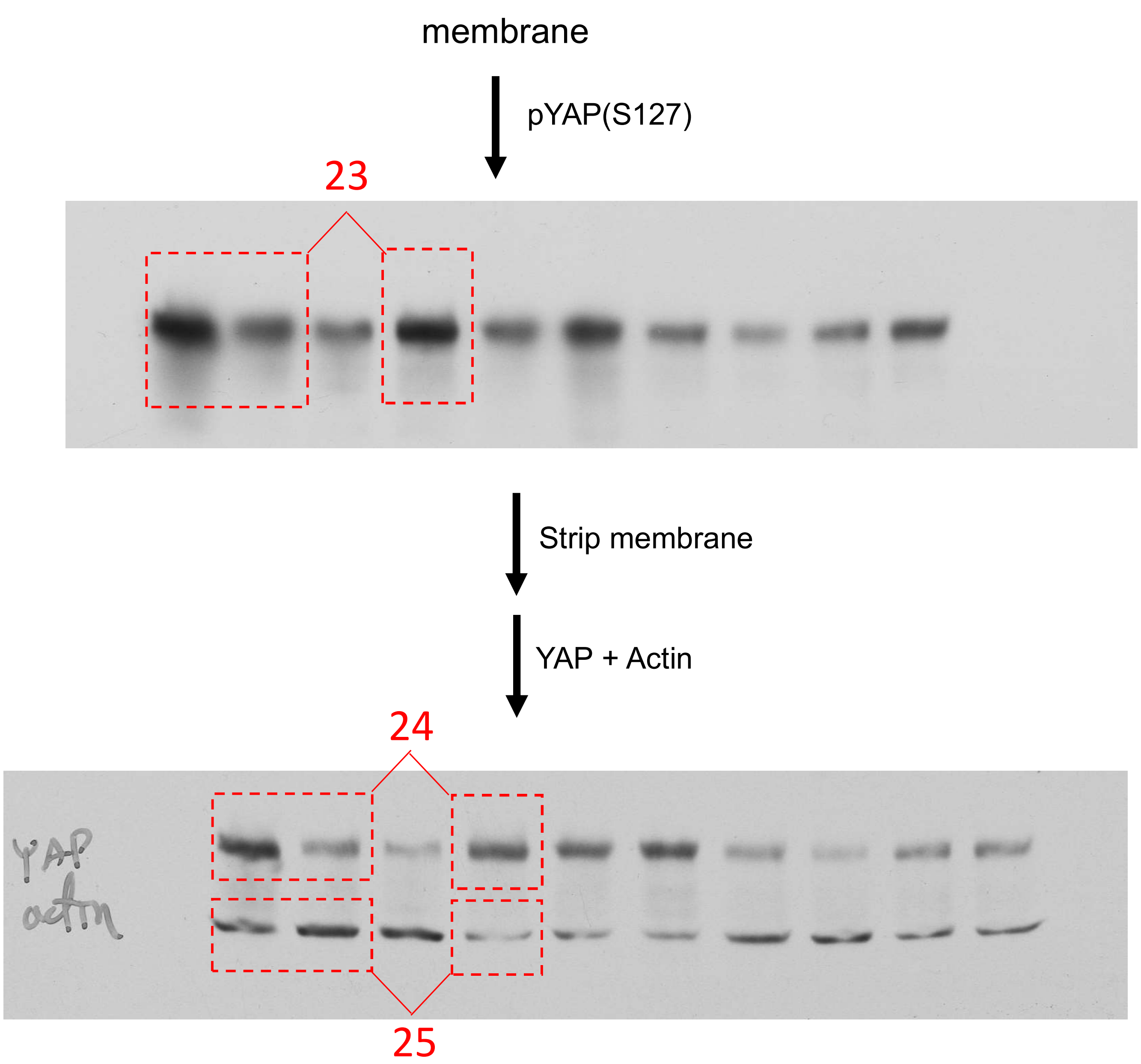

Figure 2A: Blots 26-28

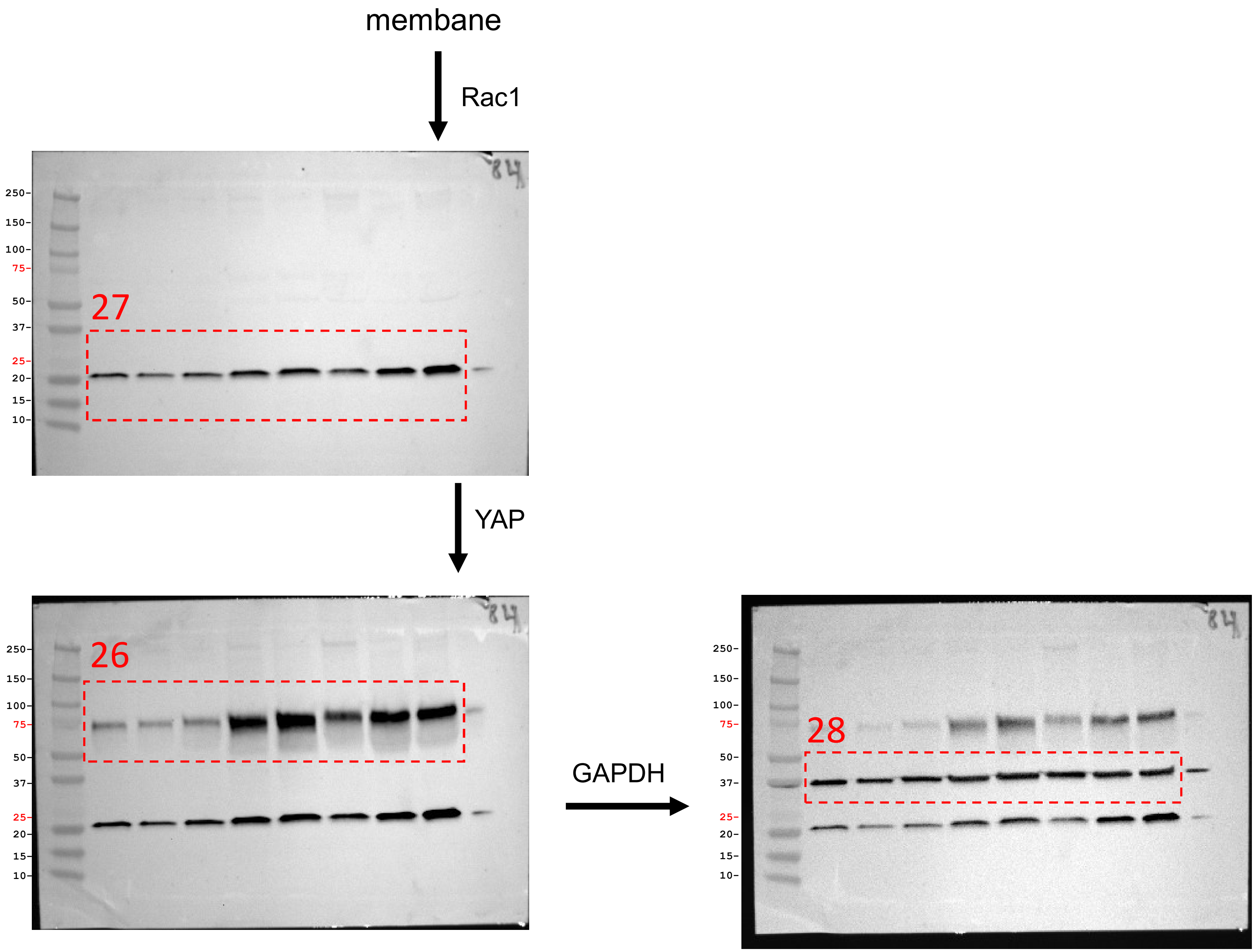

Figure 2D: Blots 29-35

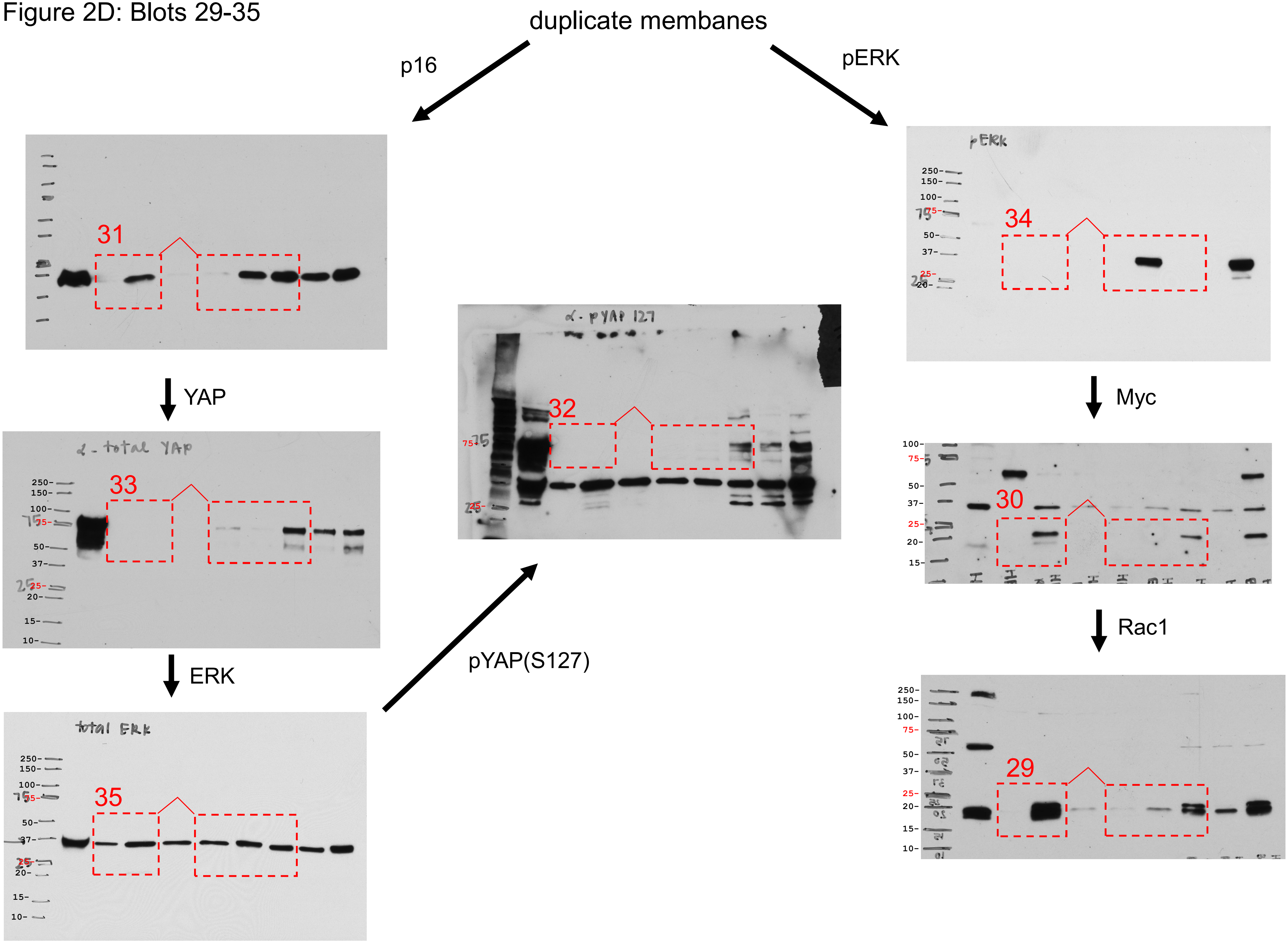

Figure 2E: Blots 36-37

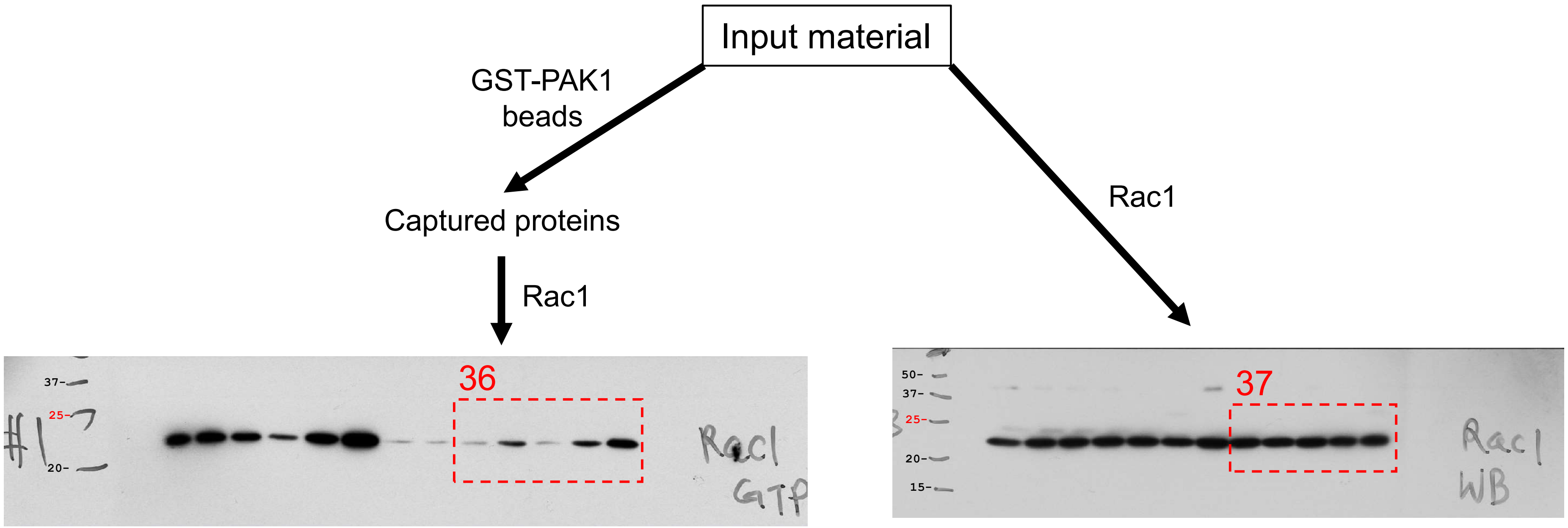

Figure 2E: Blots 38-40

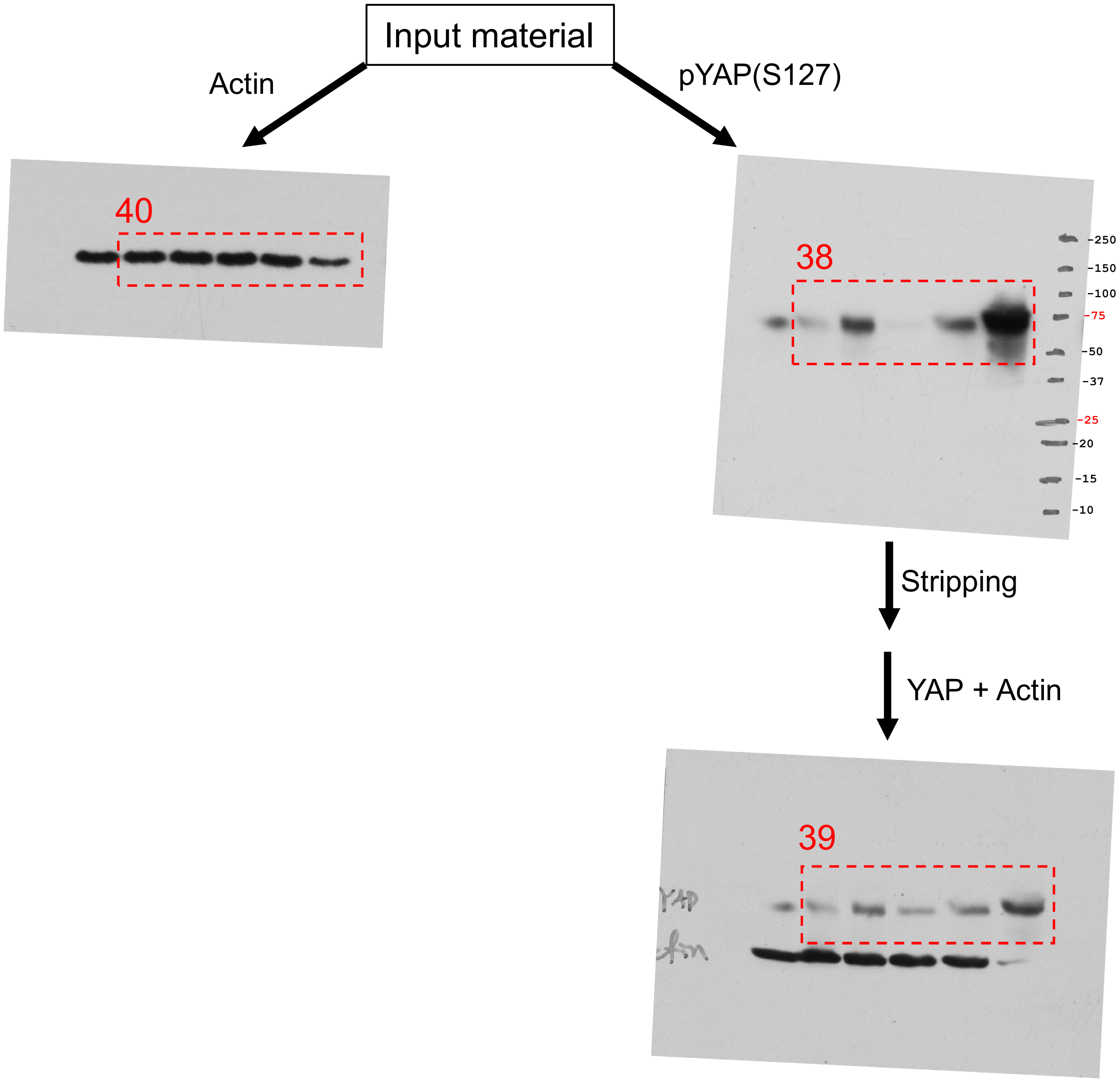

Figure 3A: Blots 41-43

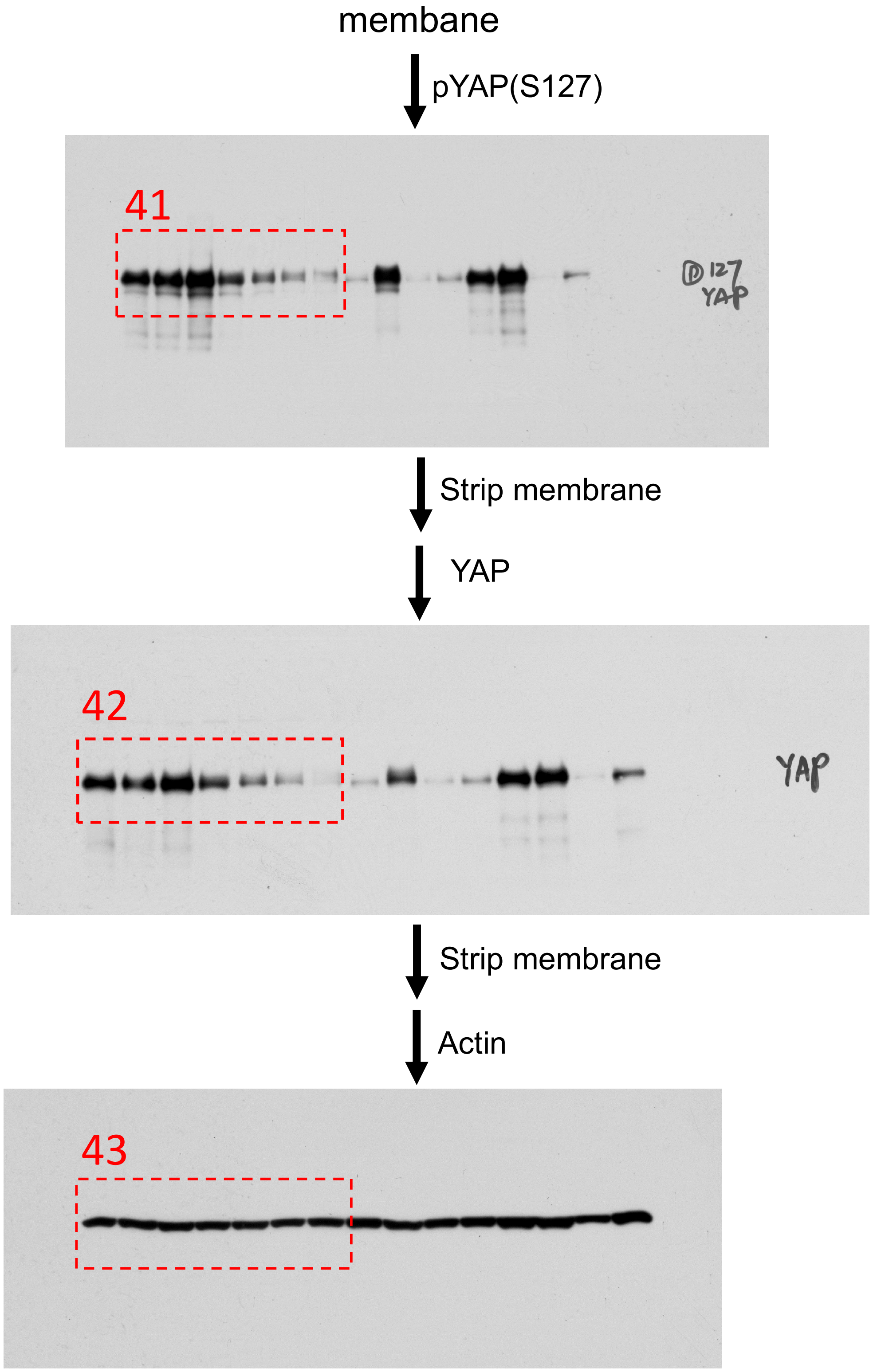

Figure 3B: Blots 44-46

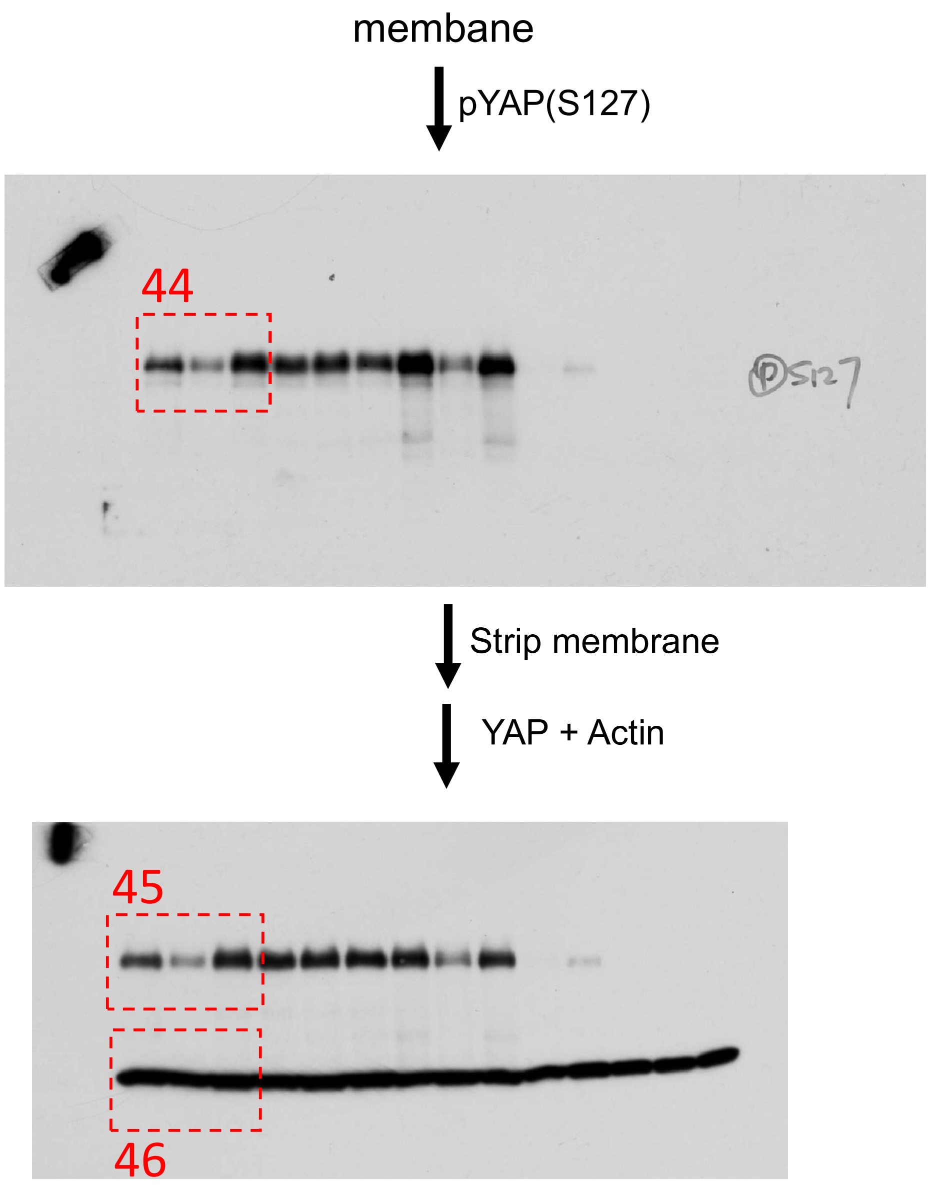

Figure 3D: Blots 47-48

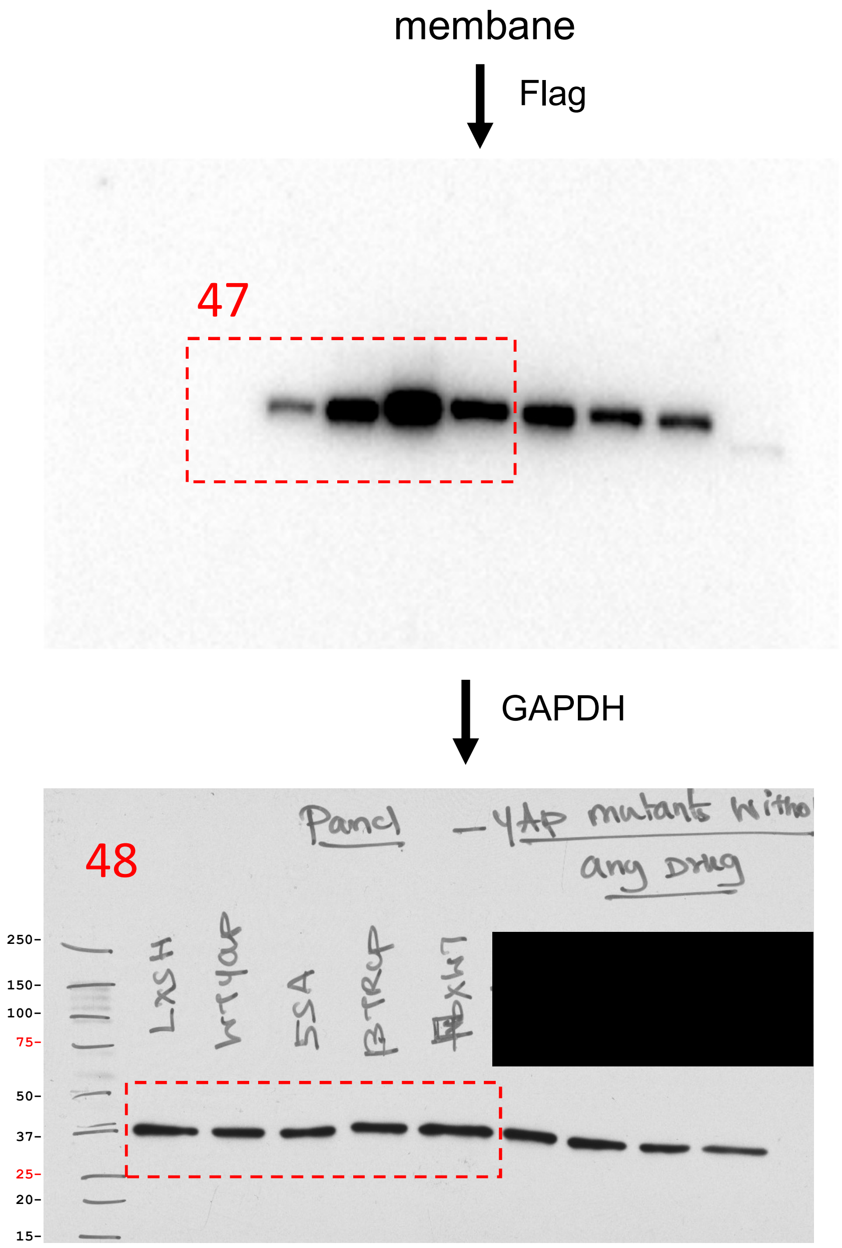

Figure 3E: Blots 49-50

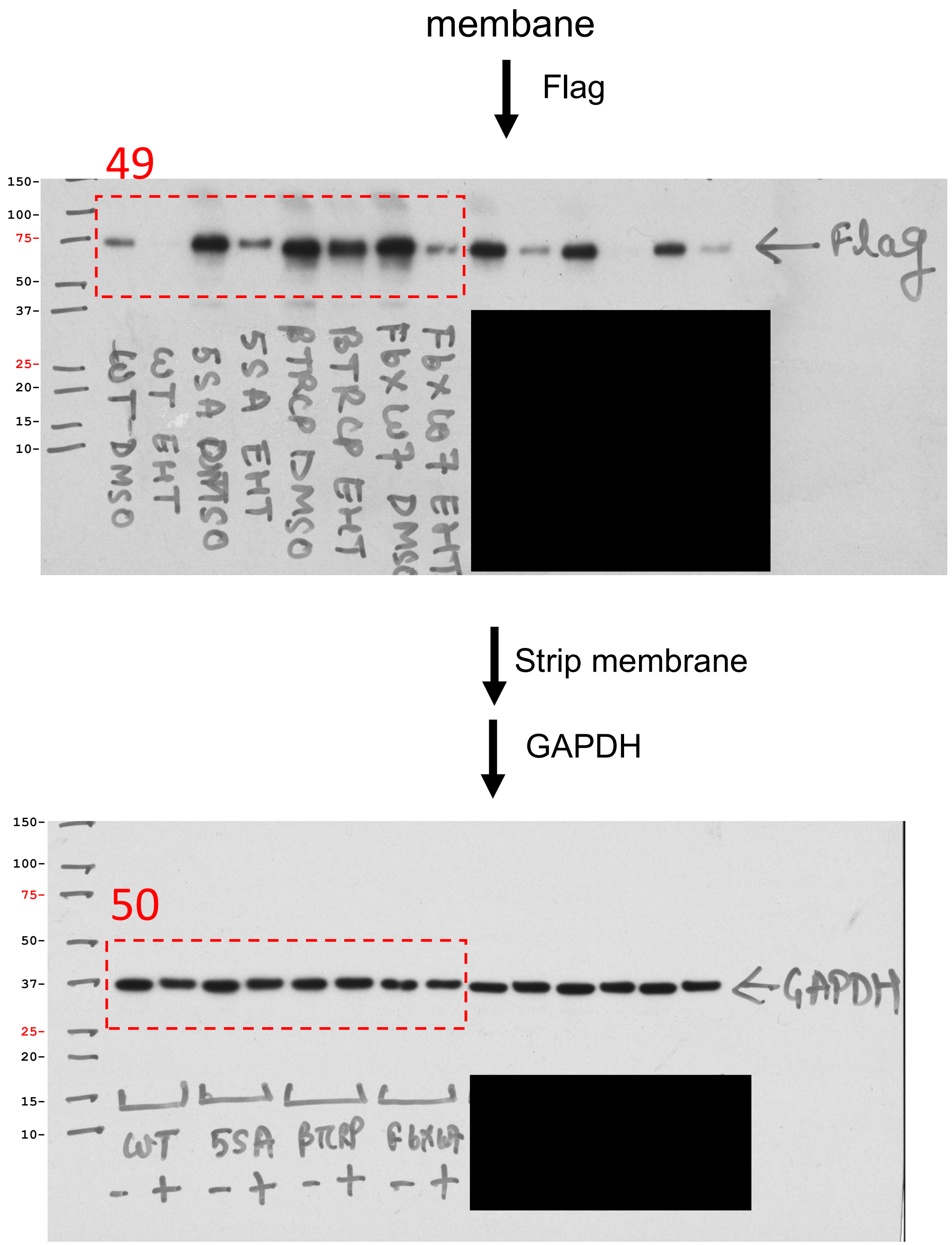

Figure 3F: Blots 51-53

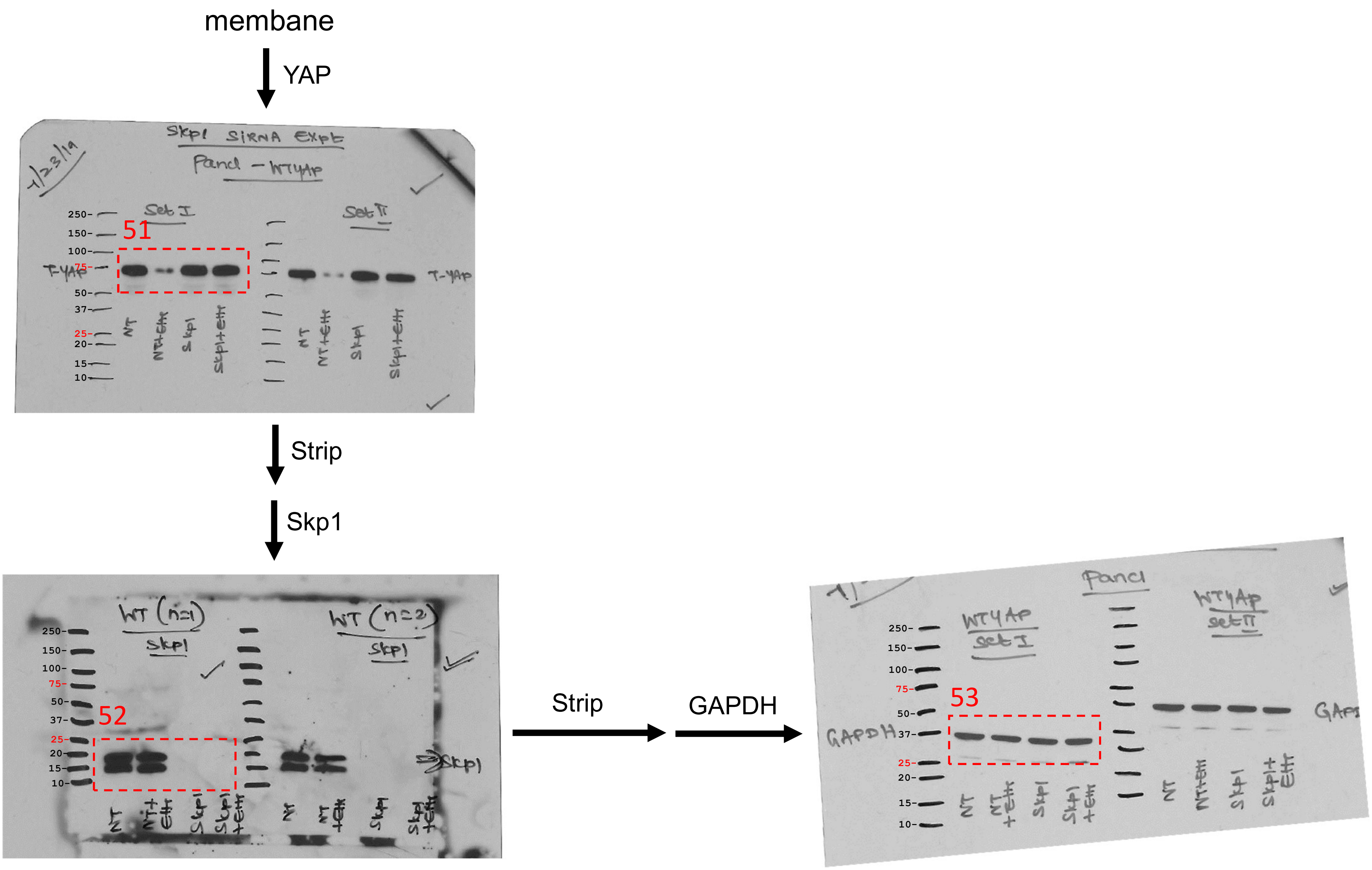

Figure 3G: Blots 54-55

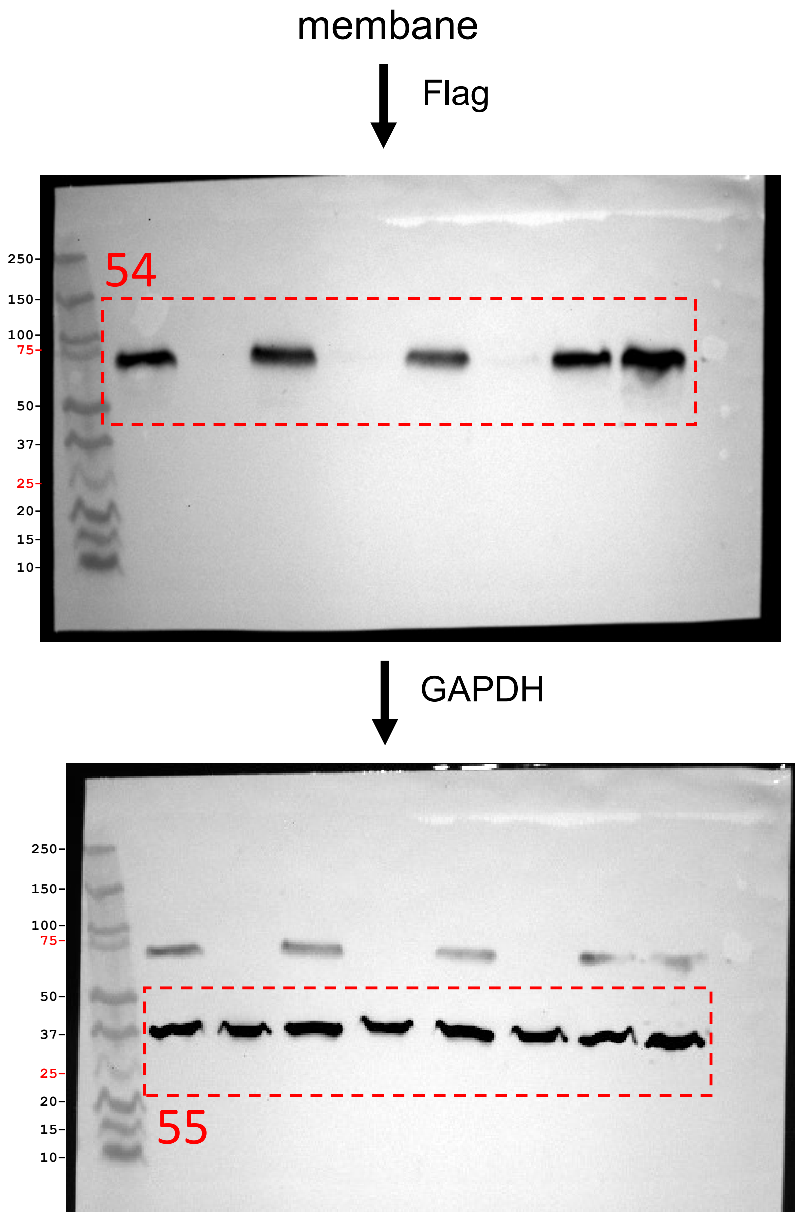

Figure 4A: Blots 56-59

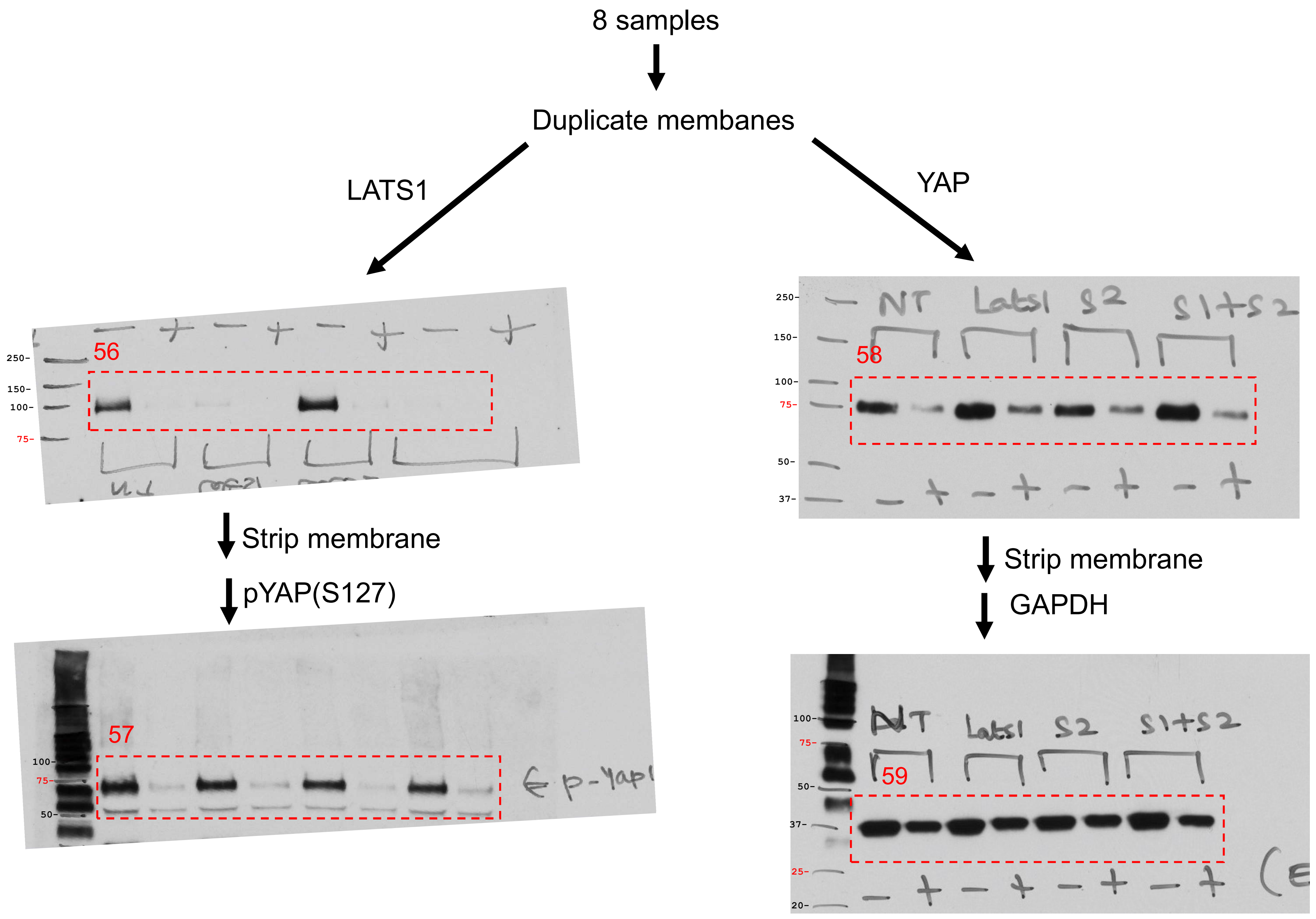

Figure 4B: Blots 60-62

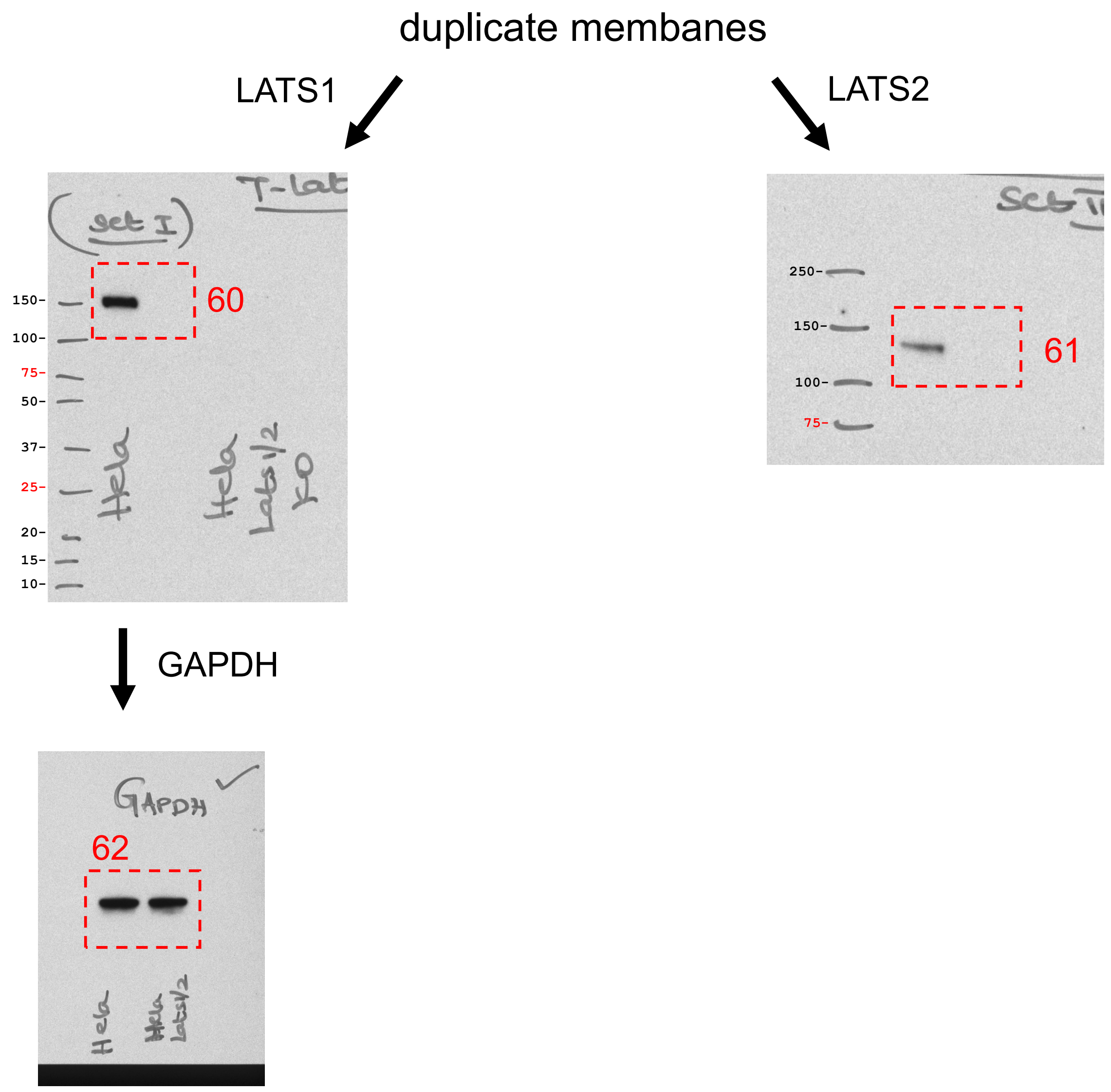

Figures 4C-D: Blots 63-66

membane

YAP

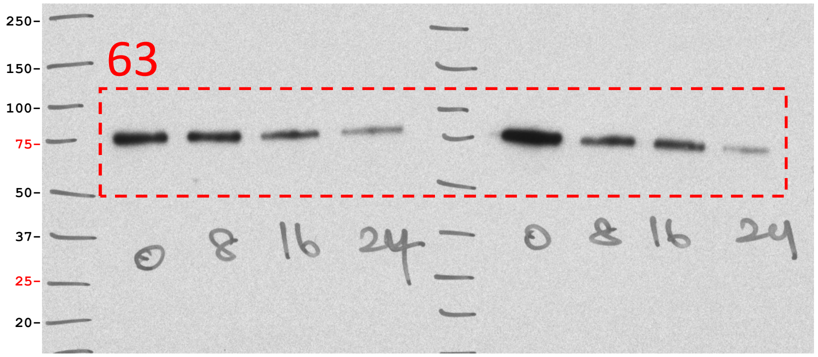

GAPDH

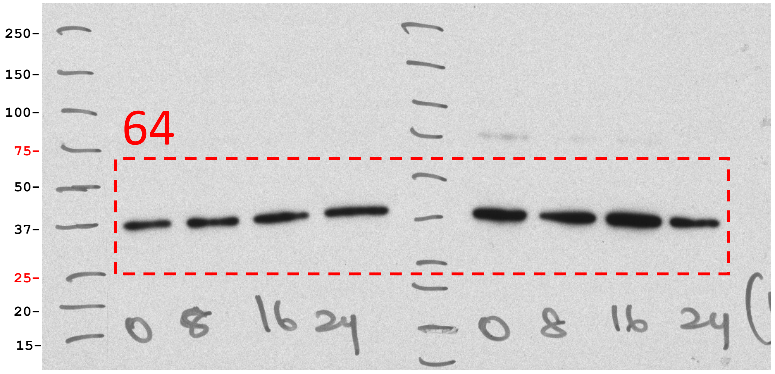

membane

YAP

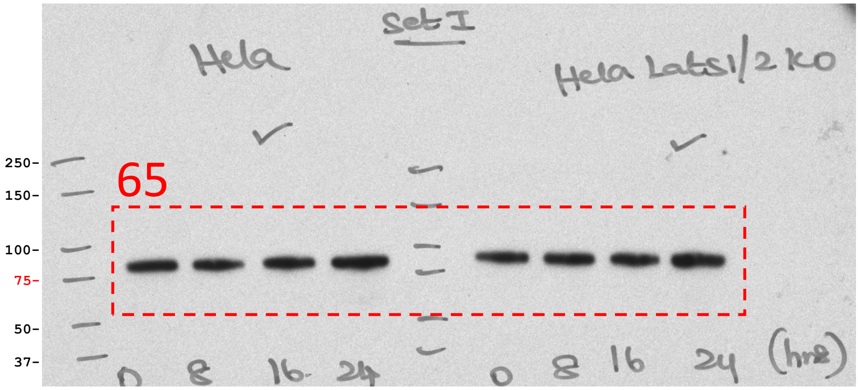

GAPDH

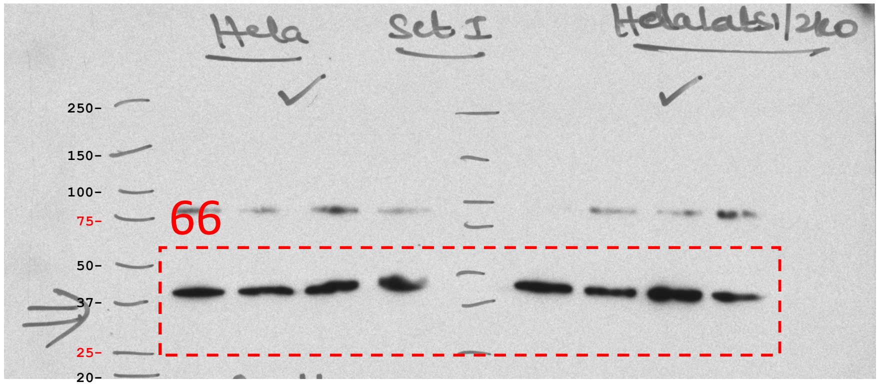

Figures 4E-F: Blots 67-70

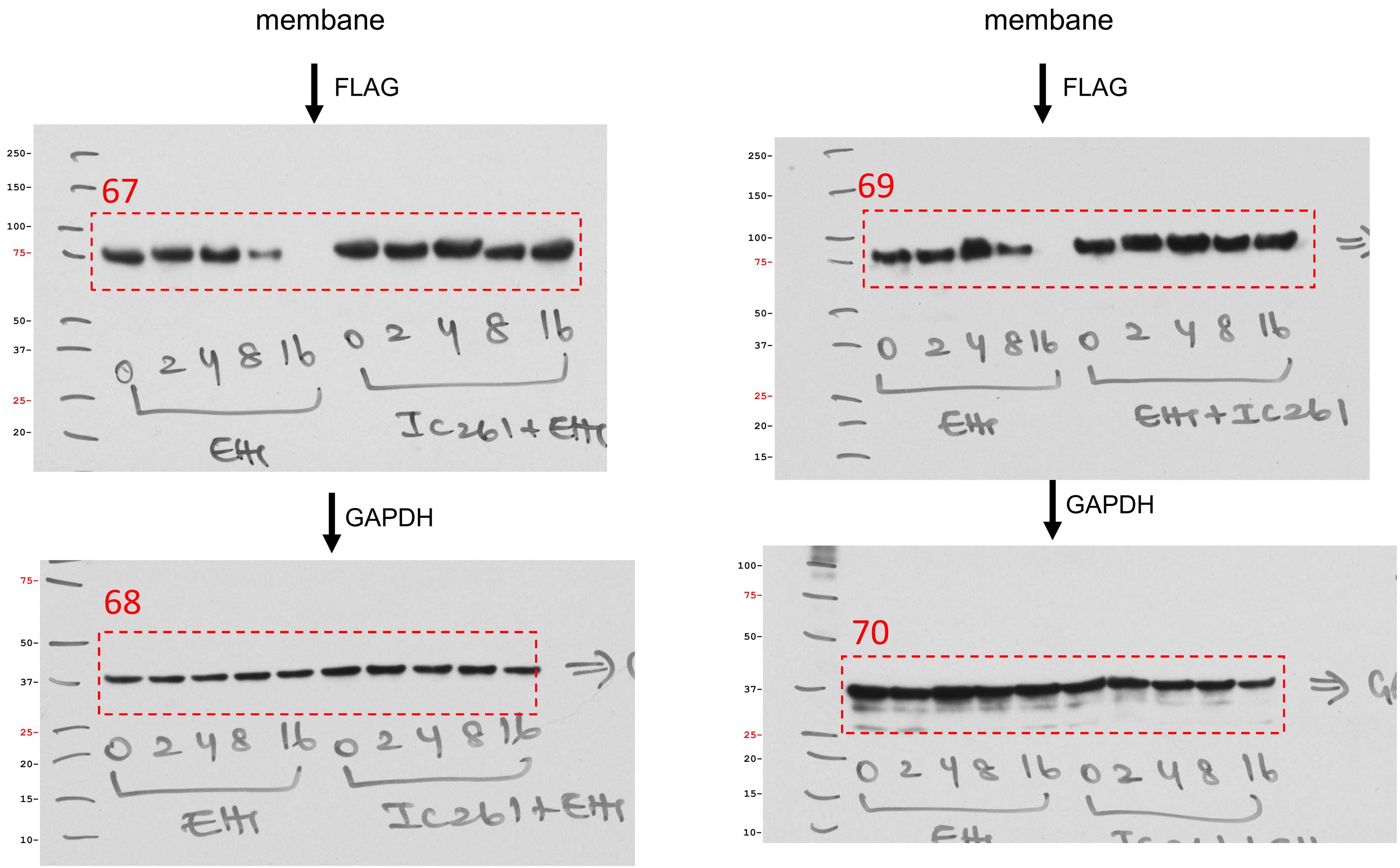

Figure S1: Blots 71-73

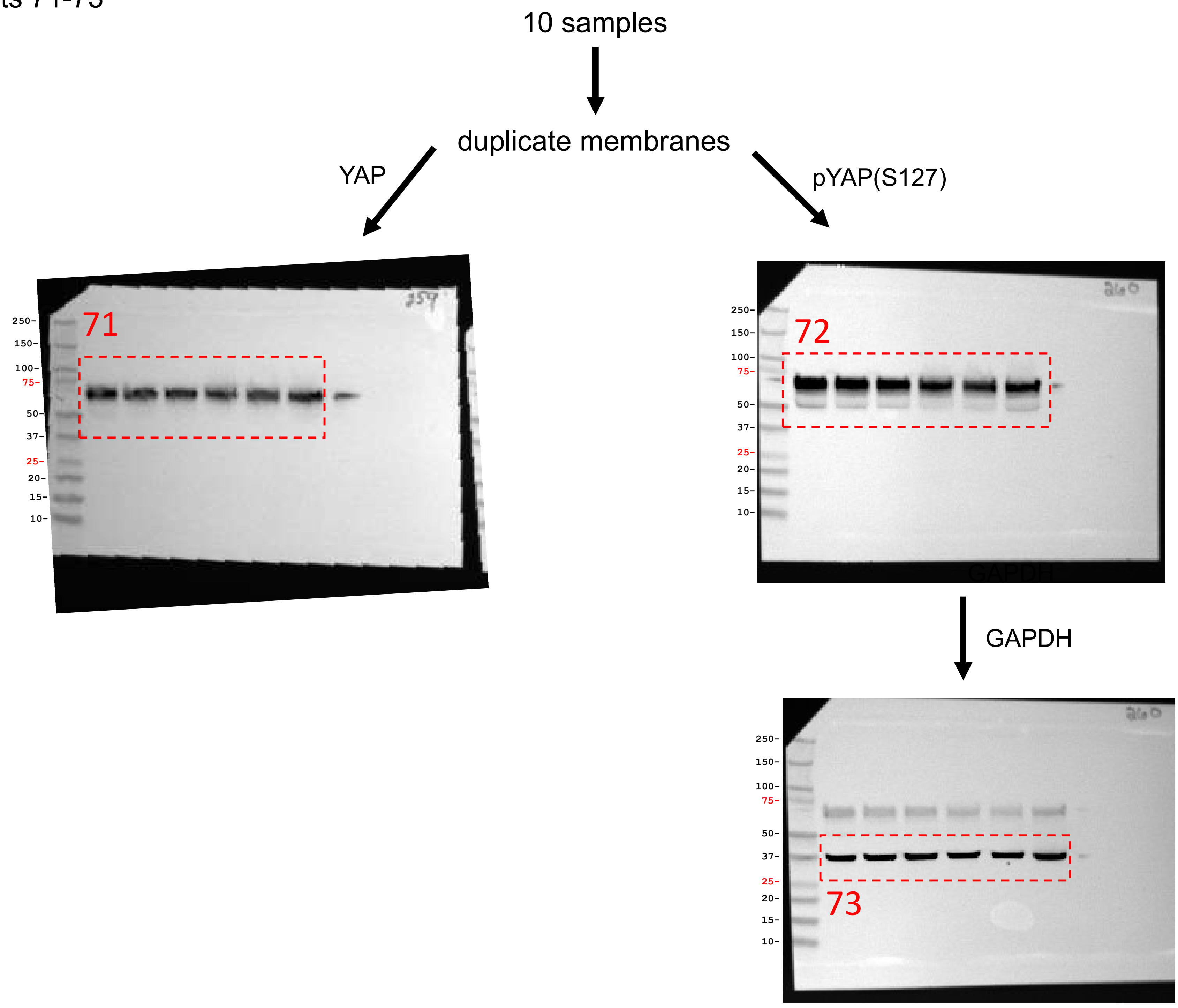

Figure S2: Blots 74-76

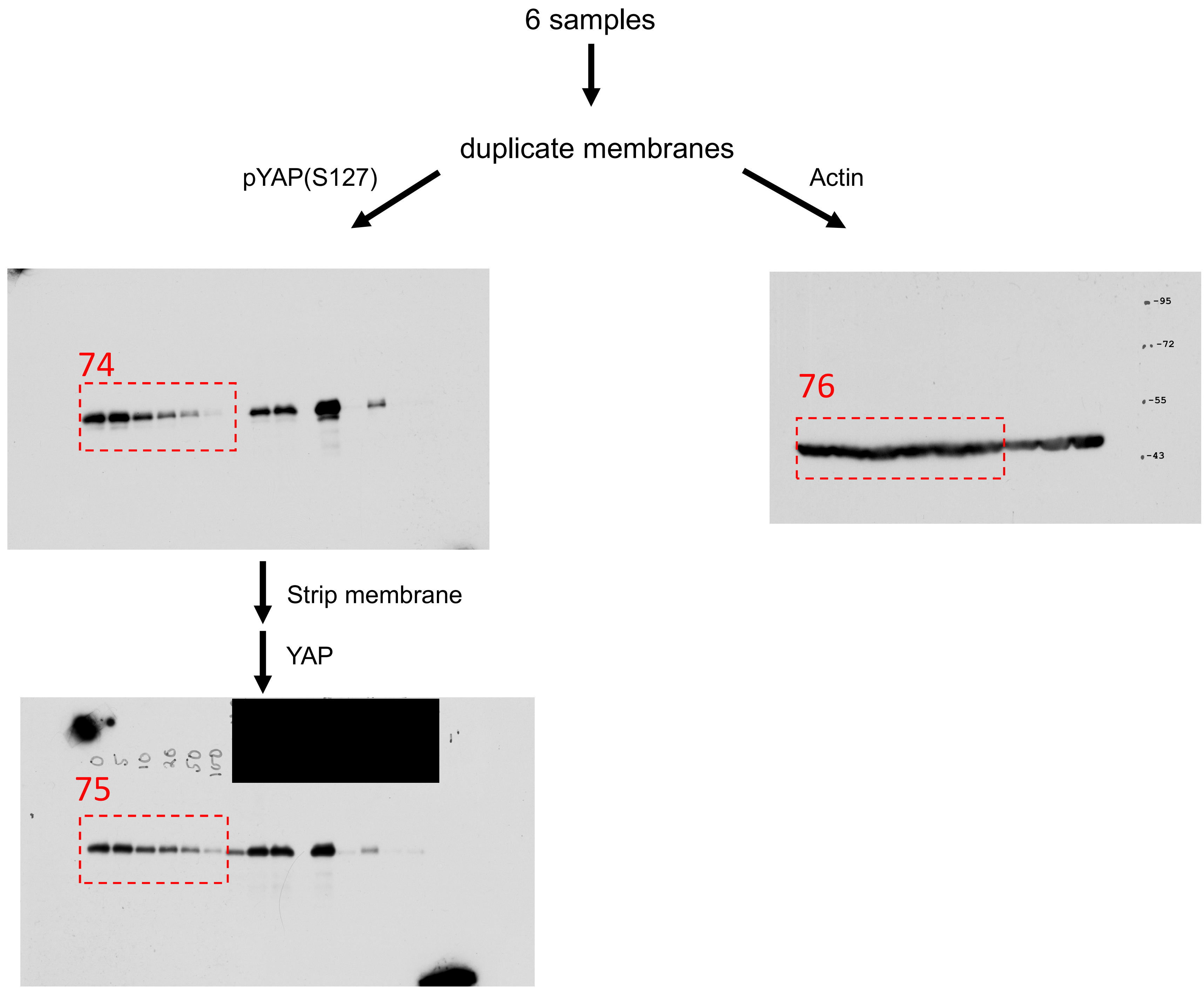

Figure S3: Blots 77-80

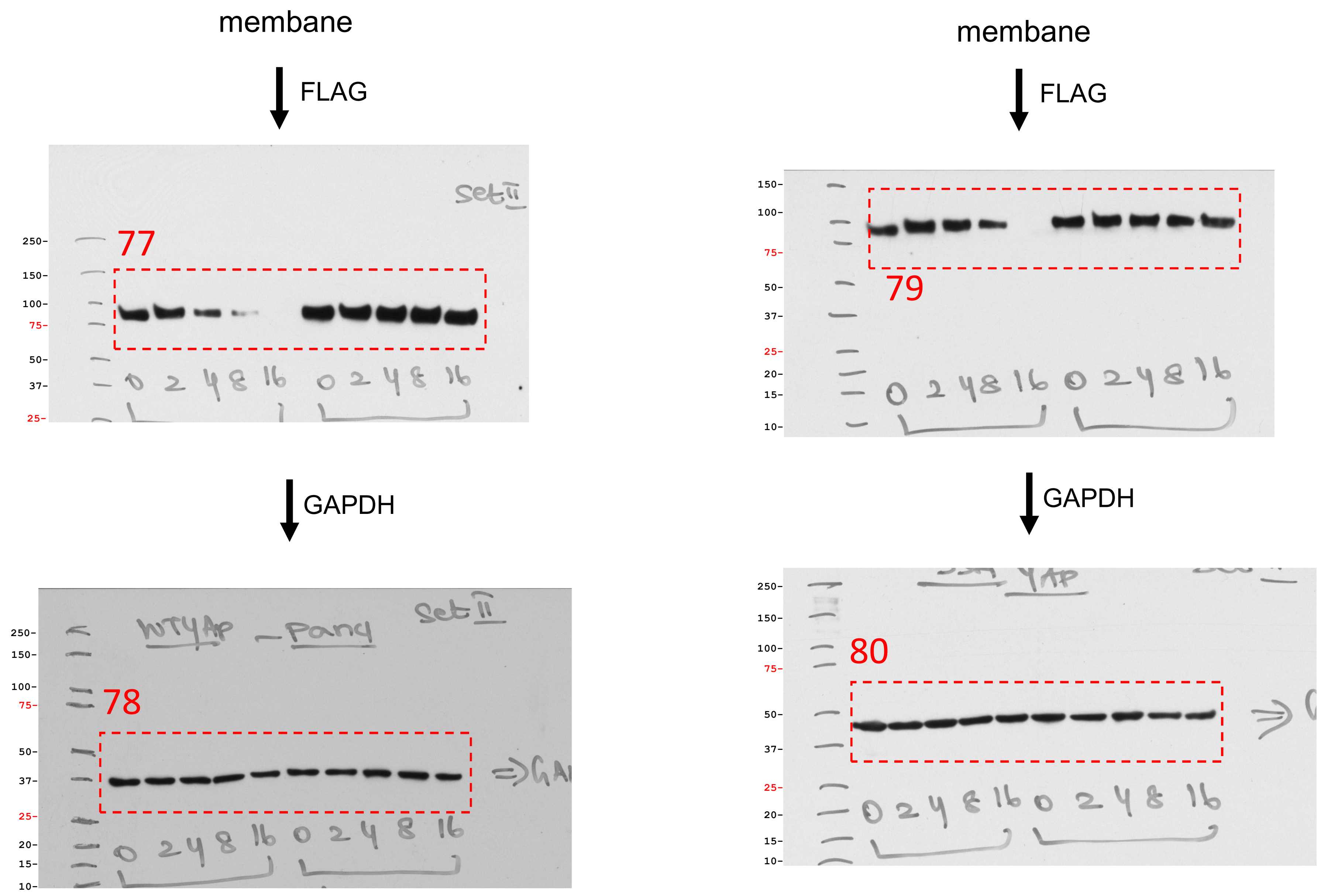

Figure S4: Blots 81-84

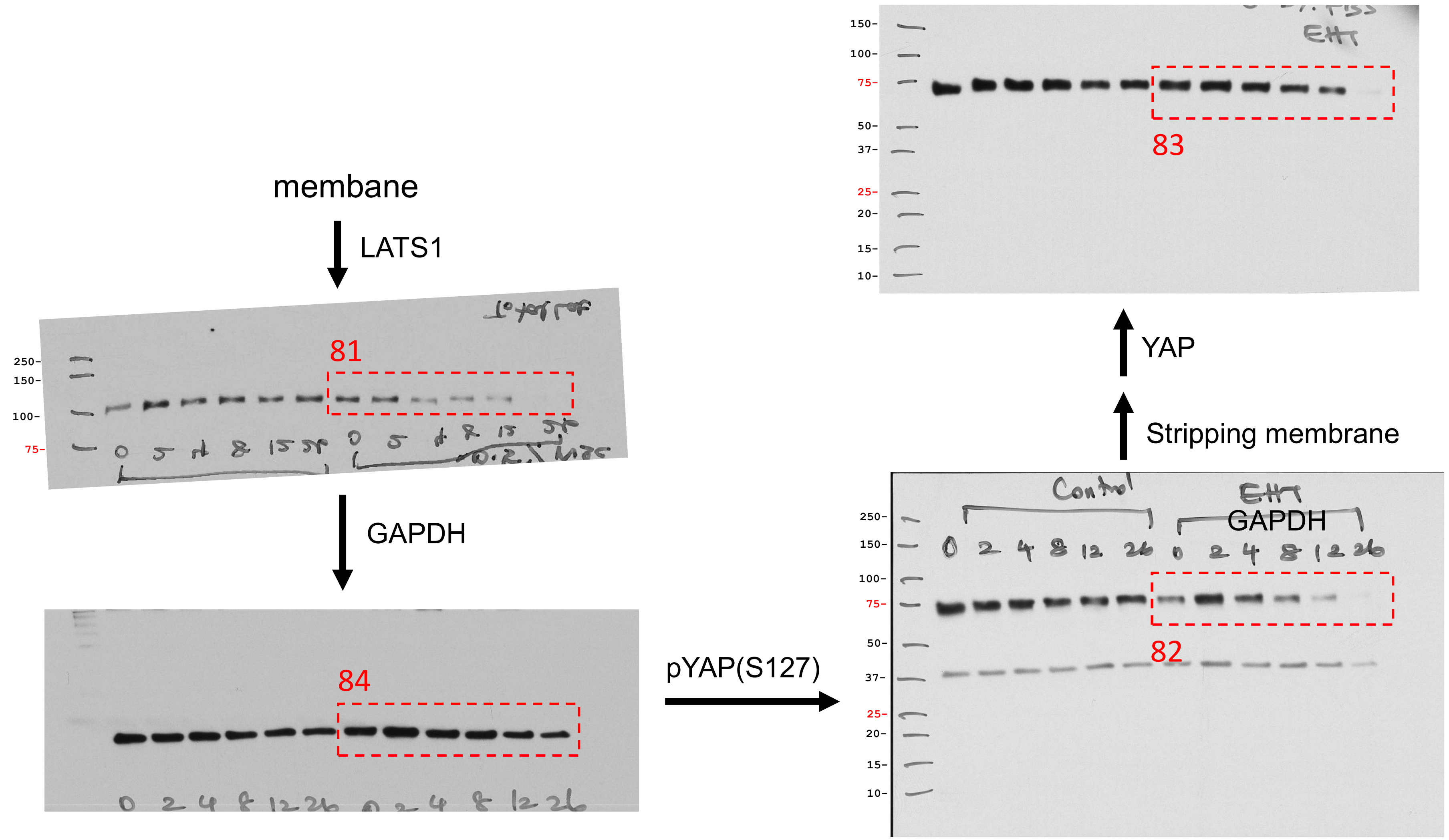

Figure S5: Blots 85-88

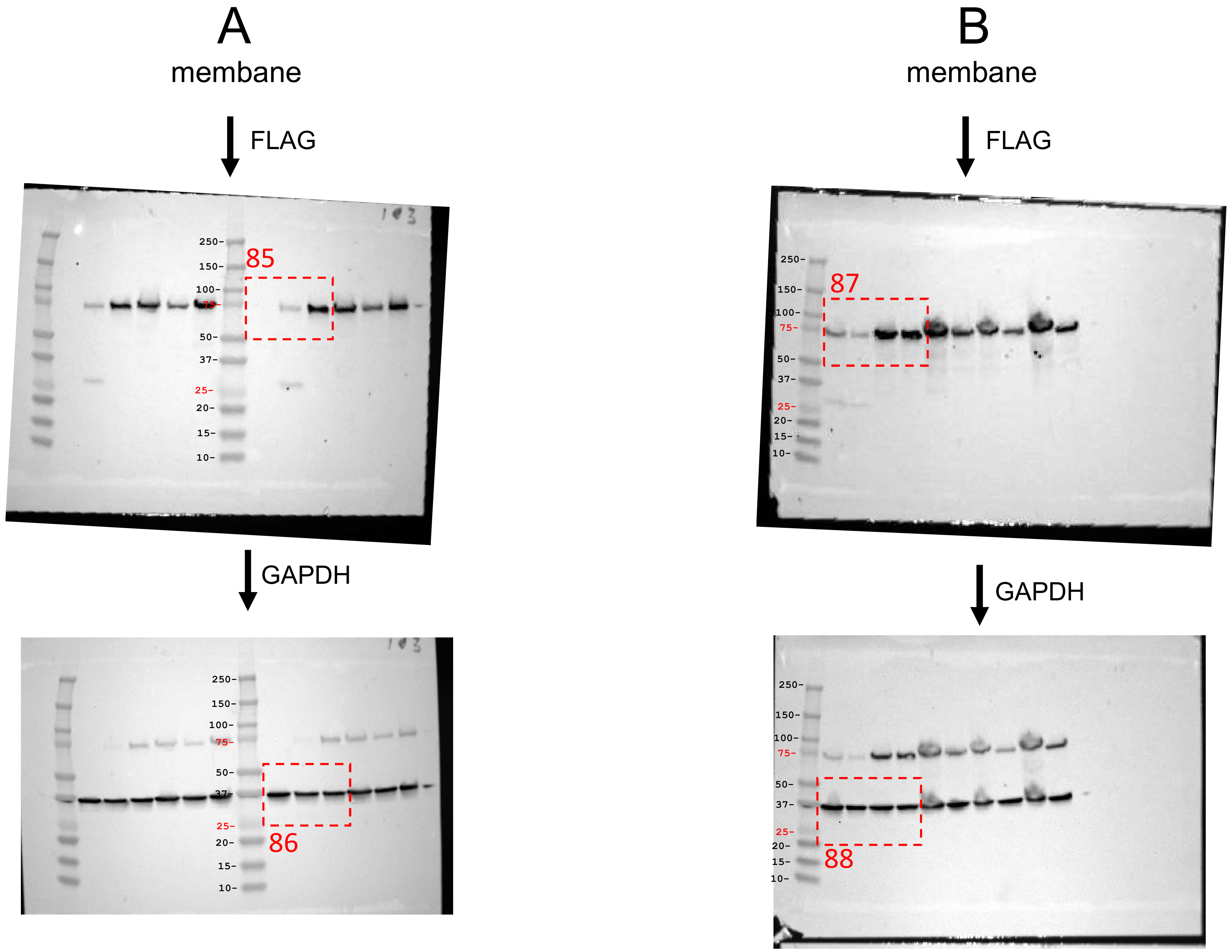

Supplement: Supplementary file 1 [file cancers-16-03605-s001.zip › cancers-3247926-supplementary.pdf]
